# Trauma research in the Nordic countries, 1995–2018 – a systematic review

**DOI:** 10.1186/s13049-020-0703-6

**Published:** 2020-03-12

**Authors:** Elisabeth Jeppesen, Valdemar Vea Iversen, Ingrid Schrøder Hansen, Eirik Reierth, Torben Wisborg

**Affiliations:** 1 Norwegian Trauma Registry, Division of Orthopedic Surgery, Oslo University Hospital, Oslo, Norway; 2 Faculty of Health Science, University of Stavanger, Stavanger, Norway; 3 Anesthesia and Critical Care Research Group, Faculty of Health Sciences, University of Tromsø – the Arctic University of Norway, Tromsø, Norway; 4 Department of Surgery, Østfold Hospital Trust, Grålum, Norway; 5 University library, University of Tromsø, the Arctic University of Norway, Tromsø, Norway; 6 Norwegian National Advisory Unit on Trauma, Division of Emergencies and Critical Care, Oslo University Hospital, Oslo, Norway; 7 Hammerfest Hospital, Department of Anaesthesiology and Intensive Care, Finnmark Health Trust, Hammerfest, Norway

**Keywords:** Nordic countries, Trauma research, Trauma system, Systematic review

## Abstract

**Background:**

Trauma is a major cause of mortality and reduced quality of life. Most trauma-related research originates from trauma centres, and there are limited available data regarding the treatment of trauma patients throughout the Nordic countries. These countries differ from economically similar countries due to their cold climate, mix of rural and urban areas, and the long distances separating many residents from a trauma centre. Research funders and the general public expect trauma research to focus on all links in the treatment chain. Here we conducted a systematic review to assess the amount of trauma-related research from the Nordic countries between January 1995 and April 2018, and the distribution of this research among different countries and different parts of the trauma treatment chain.

**Methods:**

A systematic literature search was conducted in Medline, Embase, the Cochrane Library, Web of Science, and Scopus. We included studies concerning the trauma population from Nordic countries, and published between January 1995 and April 2018. Two independent reviewers screened titles and abstracts, and performed data extraction from full-text articles.

**Results:**

The literature search yielded 5117 titles and abstracts, of which 844 full-text articles were included in our analysis. During this period, the annual number of publications increased. Publications were equally distributed among Norway, Sweden, and Denmark in terms of numbers; however, Norway had more publications relative to inhabitants. There were fewer overall publications from Finland and Iceland. We identified mostly cohort studies and very few randomized controlled trials. Studies focused on the level of care were predominantly epidemiological studies. Research at the pre-hospital level was three-fold more frequent than research on other elements of the trauma treatment chain.

**Conclusion:**

The rate of publications in the field of trauma care in the Nordic countries has increased over recent years. However, several parts of the trauma treatment chain are still unexplored and most of the available studies are observational studies with low research evidence.

## Highlights

This systematic review identified the amount and origin of trauma-related research from the Nordic countries between 1995 and 2018.

The annual number of publications increased over time, and Norwegian researchers had the most publications relative to inhabitants.

Research was lacking within several parts of the trauma chain, and most studies were observational with low research evidence.

## Background

Trauma is a major cause of mortality and reduced quality of life, especially in younger age groups [1]. Each year, trauma is responsible for 73 deaths/100,000 inhabitants globally and 29 deaths/100,000 inhabitants in the Nordic countries [1] (age-standardized mortality rates). The majority of trauma-related deaths occur in the prehospital setting [2, 3].

New trauma systems, treatment modalities, and treatment guidelines are continuously being developed. To reduce the burden of avoidable death, it is essential to have well-prepared systems with adequate distributions of resources, knowledge, and personnel [4–8]. Improvement in trauma care requires detailed knowledge of the epidemiology of trauma, patient demographics, interventions, clinical outcomes, and the patient’s journey throughout the complete treatment chain [9].

Research funders, the government, and the public expect research to be beneficial to society. With regards to trauma-related research, this implies a reasonable distribution of research focused on the different levels of trauma care, and on the links in the trauma treatment chain.

The Nordic countries differ from other countries at a similar economical level due to their cold climate, and mix of rural and urban areas [10]. The annual number of serious trauma cases is generally low, with individual hospitals handling only small numbers of seriously injured patients each year [10]. Few hospitals are defined as trauma centres [11], and many residents live a long geographical distance from a trauma centre [10]. Trauma surgery is not a recognized medical specialty. Surgeons specialized in gastroenterology performs most trauma and emergent surgery, but are supplied by several specialties, e.g. thoracic, urologic, orthopaedic and neurosurgical when appropriate. The Nordic countries are rather homogeneous, and the trauma systems are to a large extent similar. Therefore, an equal share of publications between countries would be expected.

In the present study, we aimed to systematically review the trauma research published in the Nordic countries between January 1995 and April 2018. The primary objective was to investigate the amount of trauma research published over the last 20 years. The secondary objectives were to assess the methodology used in these studies, and the distribution of research articles among different countries and different parts of the trauma treatment chain.

## Methods

We applied an integrative review method to ensure a systematic search strategy, a rigorous screening process, and inclusion of all available evidence from a variety of sources. The protocol was designed in adherence to the Preferred Reporting Items for Systematic Reviews and Meta-Analyses (PRISMA) statement and guidelines [12].

### Eligibility criteria

The search was limited to publications from between January 1995 and April 2018. We screened all human studies, published in English or Nordic language, and having at least one author from one of the Nordic countries.

### Search strategy

**Fig. 1:**
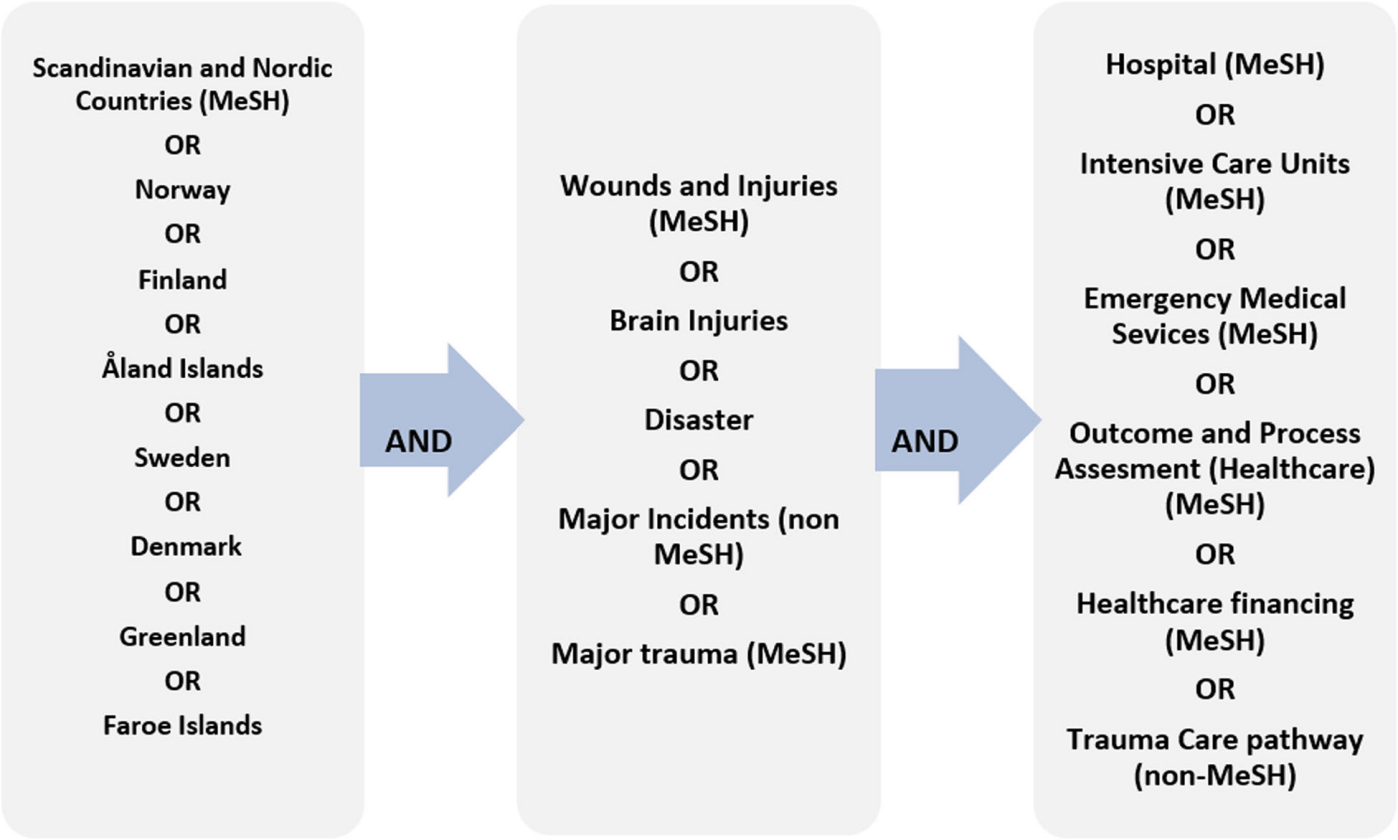
MeSH terms and text words

We performed a systematic search in the following databases: Medline, Embase, the Cochrane Library, Web of Science, and SveMed+. We used the controlled vocabulary of MeSH and the Emtree index, as well as truncated free-text searches in the search fields of Title, Abstract, and Keyword Heading (Fig. 1).

### Study selection

We screened titles and abstracts using the web-based analysis tool Covidence [13]. All identified studies were entered into the Endnote software X5, and duplications were removed. Two reviewers screened studies based on titles and abstract, and had no disagreements during this process. In a second round, two reviewers assessed full-text articles based on predetermined eligibility criteria. Any disagreement between the reviewers was discussed and resolved through discussions and consensus.

Selected studies concerned injuries that resulted in hospital admission or death, including epidemiology and treatment, in the Nordic countries. We included studies involving all levels of trauma care, in all parts of the trauma chain, in all age groups and systems, and relating to system development. We excluded studies of iatrogenic injuries or less severe injuries (injuries not resulting in hospitalization), and studies performed by Nordic researchers concerning trauma in non-Nordic countries.

For every included reference, we recorded the following five main variables: year published, country of origin, part of the treatment chain, type of injury, and study design. These variables were analysed using SPSS Statistics, version 24 (IBM Corp., Armonk, NY).

## Results

**Fig. 2:**
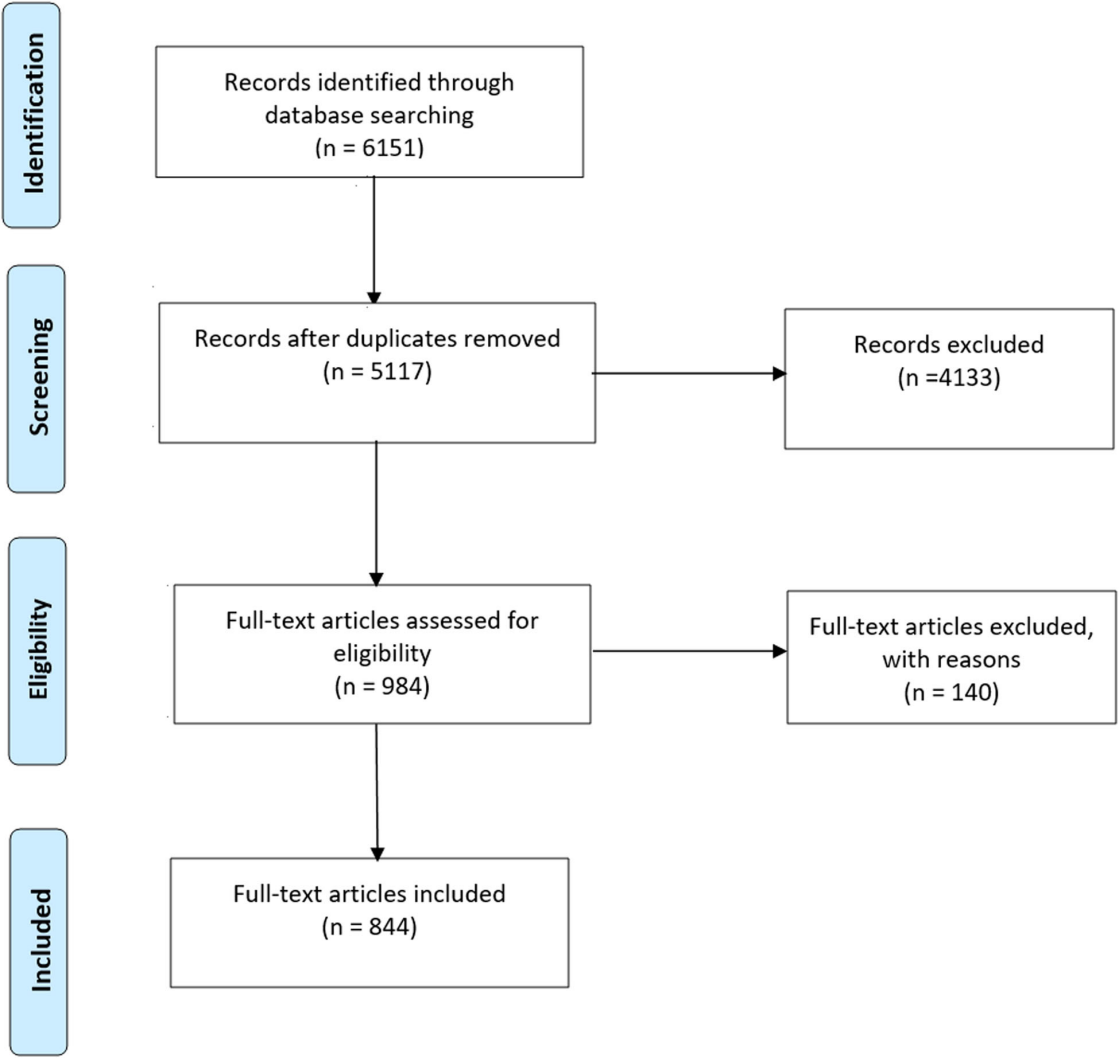
PRISMA flow diagram for selection of included and excluded studies

The search yielded a total of 6151 references at the title and abstract level. After removal of duplicates, 5117 papers were screened to determine whether they met the inclusion or exclusion criteria. A total of 984 publications were read in full text, of which 844 were included in this review. Figure 2 summarises the search process.

**Fig. 3:**
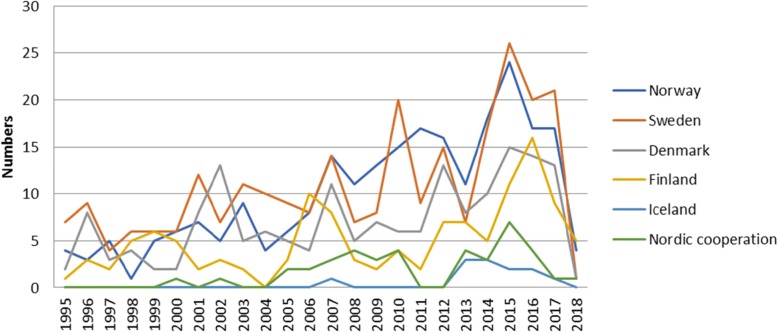
Distribution of included studies, sorted by year

**Fig. 4:**
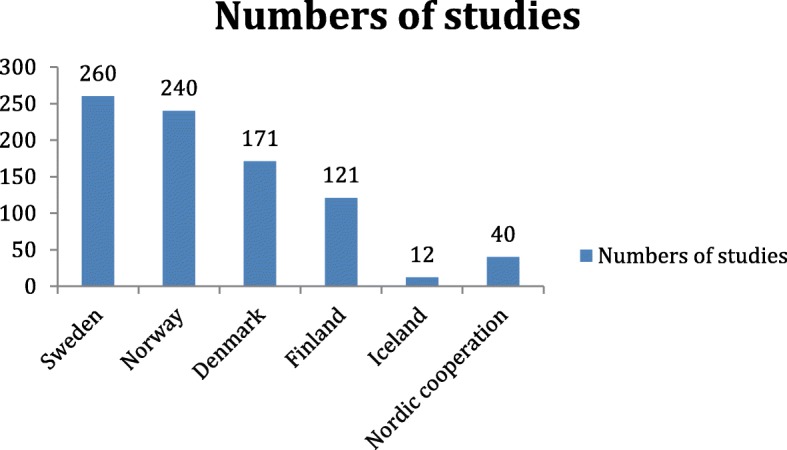
Distribution of included studies, sorted by Nordic countries

The number of annual publications in the Nordic countries increased throughout the study period. Figure 3 shows the distribution of studies per year. The numerical distribution of publications was nearly equal between Norway, Sweden, and Denmark, while fewer studies were published from Finland and Iceland. The numbers of papers relative to each country’s population (as per 2005) were as follows: Norway, 52/million inhabitants; Sweden, 29/million inhabitants; Denmark, 32/million inhabitants; Finland, 40/million inhabitants; and Iceland, 41/million inhabitants. Figure 4 shows the distribution of studies per country. The majority of published studies examining the trauma population were performed in Norway and Sweden.

The included publications predominantly described cohort studies (61%), while randomised controlled trials (RCTs) accounted for only 2%. The level of evidence was low, since the majority of studies were retrospective cohort studies (Table 1).

**Table 1:** Study design of included studies from Nordic trauma research, 2005–2018

Study design	Number of studies, N (%)
Meta-analysis	1 (0.1)
Randomised double-blinded controlled trials	3 (0.4)
Randomised controlled trials	13 (1.6)
Cohort	505 (61.2)
Case-control	35 (4.2)
Case series	259 (31.4)
Case report	10 (1.2)
Total	825 (100)

The majority of included studies focused on “epidemiology” (365 studies, 43.2%). Most studies examining a stage of treatment were cohorts and case series. RCTs were mostly performed to investigate the treatment options in early rehabilitation after trauma. Pre-hospital research accounted for 136 (16.1%) of all included studies, which was three-fold more than the studies performed in other stages in the trauma chain. The other stages were described in approximately 5% of the studies. Even fewer studies focused on trauma systems and trauma registers, accounting for approximately 3% of the included studies (Table 2).

**Table 2:** Distribution of studies in different parts of the treatment chain in Nordic trauma research, 2005–2018

Treatment chain	Number of articles, N (%)
Epidemiology	365 (43.2)
Pre-hospital care	136 (16.1)
Emergency room and trauma assessment	48 (5.7)
Surgery	48 (5.7)
Intensive care	44 (5.2)
Late rehabilitation	42 (5.0)
Early rehabilitation	34 (4.0)
Trauma systems	32 (3.8)
Preventive care	31 (3.7)
Registers and scoring systems	27 (3.2)
Diagnostics	26 (3.1)
Team training	7 (0.8)
Ordinary ward care	4 (0.5)
Total	844 (100)

Within the category “type of injury”, most articles discussed several severe injuries in multiple patients, i.e. included mixed materials. Approximately 20% of studies described multi-trauma and traumatic brain injuries. Other types of severe injuries were rarely described in a trauma context. The included RCTs predominantly described various treatment options for head and spinal injuries (Table 3).

**Table 3:** Distribution of studies describing different types of injuries in in Nordic trauma research, 2005–2018

Type of injuries	Number of studies, N (%)
Several serious injuries	314 (37.2)
Head injuries	219 (25.9)
Multi-trauma	181 (21.4)
Spinal injuries	52 (6.2)
Thorax injuries	18 (2.1)
Abdominal injuries	12 (1.4)
Lower and upper limb injury	6 (0.7)
Accidental hypothermia	5 (0.6)
Neck and throat trauma	4 (0.5)
Pelvic injuries	2 (0.2)
Not defined type of injury	31 (3.7)
Total	844 (100)

Table 4 shows that most of the reviewed research focused on a mixed population with different types of injuries, multi-trauma, and head injuries.

**Table 4:** Numbers of studies included according to type of injury and treatment chain, Nordic trauma research, 2005–2018

Type of injuries/ Treatment chain	Prevention	Pre-hospital	Emergency room	Surgery	Intensive - care	Rehabilitation	Trauma system	Epidemiology	Registries and quality	Diagnostic	Team training
Mixed severe injuries	22	62	9	8	7	3	7	180	10	2	
Head	3	15	1	14	15	49	4	103	3	12	
Multi-trauma	4	45	34	5	17	2	12	47	6	5	4
Spine				1	1	20	2	20	6	2	
Thorax		1	1	6			1	7		2	1
Abdominal				8				2		1	
Extremity		1				2		3			
Hypothermia		3			2						
Neck				3						1	
Pelvic				2							
Not defined	2	9	3	1	2		6	3	2	1	2
Total	31	136	48	48	44	84	32	365	27	26	7

## Discussion

Our present results showed that the annual number of trauma-related publications from Nordic Countries increased during the years 2005–2018. Most included studies had an observational study design with low evidence. Additionally, the studies were predominantly retrospective, and thus less robust, limiting both clinical impact and applicability [14].

Trauma has generally received more international attention in recent years, and many systems have been introduced to improve quality in trauma care. For example, comparing observed survival with the probability of survival calculated from large trauma registries has gained popularity as a method of evaluating trauma care effectiveness [15]. The increased focus on trauma care may be attributable to the increasing number of terrorist attacks in the Nordic countries [16, 17].

When adjusted for inhabitants, Norway published more trauma-related articles compared to the other countries, while Denmark and Sweden lagged behind. There are probably several reasons why we are seeing an increase in publication rate in all Nordic countries and especially in Norway. Established trauma systems have possibly increased the interest for research in traumatology in all countries. Trauma team training (BEST) has been introduced and implemented in Norway [18]. In addition, research has been funded in Norway by the Norwegian Air Ambulance Foundation for prehospital research. The establishment of trauma registries in some of the countries has presumably contributed, but these are of recent date and thus have not had a major impact on research conducted during the period studied. In all, we assume that increased attention and work on several elements at national level has contributed to this increase in publication rate.

We did not find any systematic reviews with pooled results (meta-analyses), likely due to the number of observational studies published in all Nordic countries, and the lack of RCTs. Most of the epidemiological studies involved the collection and summarisation of data available for different types of injuries. The patient groups included in the studies were heterogeneous, with a wide range of different injuries. Thus, it would not be feasible to pool the results from these studies.

Studies on all stages of trauma care were largely focused on only a few different types of injuries. The most frequently described types of injuries were traumatic brain injuries, multiple traumas, and spinal injuries. Few studies examined injuries to the chest, abdomen, or extremities as isolated entities. Moreover, only a limited number of studies investigated rehabilitation and prevention. Registry-based studies were frequent. National trauma registries have been implemented in Norway and Sweden during the last decade, and will likely be essential for providing reliable data for further research. The Norwegian Trauma Register is the only register that includes over 90% of patients, with an average of 7500 patients each year (13% with an injury severity score (ISS) > 15).

A large proportion of the included studies had a cohort design, and thus had a limited impact on important research questions. Only 2% of the included studies were randomized controlled trials, and few papers fulfilled the requirements for the highest levels of quality according to the GRADE principles [19].

The identified limitations regarding study design in trauma research are somewhat worrying. With the emergence of new technology and more compact devices, introducing these developments into the trauma setting will require standardization of the risks and benefits. Descriptive studies will be unable to answer research questions regarding intervention effectiveness. Critical voices have even speculated on whether clinical practices in some parts of trauma care have been founded on tradition and old dogma.

Although it is challenging, in the future, it will be important to perform studies with good methodological designs. We probably cannot expect to reach the highest level of evidence in this field, as heterogeneity will continue to be a hindrance. However, it is possible to make improvements. Randomized studies without proper blinding may still achieve a high level of evidence. Additionally, it is possible to conduct large-scale observational studies and to investigate causality. Interventional studies focused on trauma with a high level of evidence have been performed in non-Nordic countries [20, 21]. In the future, similar studies should be pursued in the homogeneous Nordic countries. Cross-border collaboration may be crucial to examine an adequate volume of patients.

### Limitations

The material reviewed in this study was handled based on strict inclusion and exclusion criteria; however, the title and abstract screening was performed by three different authors. Despite extensive searches of available web resources, university libraries, and the national library, five articles could not be retrieved for full-text evaluation, and were thus excluded. As in all literature searches, the search string was a factor that limited which studies were identified. As trauma is a large study area, it is possible that an even wider search may have led to the inclusion of more references.

## Conclusion

The annual rate of publications in the field of trauma care in the Nordic countries has been increasing over the last two decades. However, there remains a lack of research focusing on several parts of the trauma treatment chain, and most of the available studies are observational studies with low research evidence. There is a lack of studies examining patients with severe injuries in the trauma context, as well as few studies investigating patient transfer between levels.

## Supplementary information

**Additional file 1.** Supplementary data, included papers.

## Data Availability

We have submitted our detailed list over included studies as supplementary material.
