# Supplementary material for: Trauma research in the Nordic countries, 1995–2018 – a systematic review
Source: Scand J Trauma Resusc Emerg Med. 2020 Mar 12;28:20. doi: 10.1186/s13049-020-0703-6 (PMC7069175; doi:10.1186/s13049-020-0703-6)
Supplement: Supplementary file 1 — Additional file 1. Supplementary data, included papers. [file 13049_2020_703_MOESM1_ESM.docx]

**Supplementary data, included papers.**

1. Brorsson C, Kruse IM. Traumatology at small hospitals. Prompt care saves lives. Läkartidningen. 1995;92(44):4107-8.

2. Finset A, Dyrnes S, Krogstad JM, Berstad J. Self-Reported Social Networks and Interpersonal Support 2 Years after Severe Traumatic Brain Injury. Brain Injury. 1995;9(2):141-50.

3. Gordon E, von Holst H, Rudehill A. Outcome of head injury in 2298 patients treated in a single clinic during a 21-year period. Journal of Neurosurgical Anesthesiology. 1995;7(4):235-47.

4. Grimsmo A, Snoen SE. Accidental injuries, violence and self-inflicted injuries in a local community: Incidences, types of accidents and relation to alcohol. Tidsskrift for den Norske Laegeforening. 1995;115(20):2546-51.

5. Gustafsson Å, Sonesson P. Good surgical care at a county hospital. Evaluation of 5 years of emergency traumatology. Läkartidningen. 1995;92(44):4092-4.

6. Jonung T, Parsson H, Norgren L. Vascular Injuries in Sweden 1986-1990 - the Result of an Inquiry. Vasa-Journal of Vascular Diseases. 1995;24(2):130-4.

7. Kuisma M, Suominen P, Korpela R. Paediatric out-of-hospital cardiac arrests--epidemiology and outcome. Resuscitation. 1995;30(2):141-50.

8. Larsen LB, Poulsen TK, Johannsen HG. The age-dependent incidence of injuries due to road traffic accidents in Odense, Denmark from 1980 to 1992. Scandinavian Journal of Social Medicine. 1995;23(3):150-5.

9. Larsen MS, Jorgensen HR. [Mortality among victims of traffic accidents with multiple injuries. A descriptive study 1986-1990]. Ugeskrift for laeger. 1995;157(48):6716-9.

10. Levi R, Hultling C, Seiger A. The Stockholm spinal cord injury study. 3. Health-related issues of the Swedish annual level-of-living survey in SCI subjects and controls. Paraplegia. 1995;33(12):726-30.

11. Ponzer S, Bergman B, Brismar B. Sociodemographic Characteristics and Criminality in Victims of Firearm Injuries. Journal of Trauma-Injury Infection and Critical Care. 1995;38(6):845-50.

12. Svanstrom L, Ekman R, Schelp L, Lindstrom A. The Lidkoping Accident Prevention Programme--a community approach to preventing childhood injuries in Sweden. Injury prevention : journal of the International Society for Child and Adolescent Injury Prevention. 1995;1(3):169-72.

13. Wester K. Surgical treatment of acute head injuries, especially epidural hematomas. Current and future practice in Norway. [Norwegian]. Tidsskrift for den Norske laegeforening. 1995;115(13):1623-6.

14. Ytterstad B, Wasmuth HH. The Harstad Injury Prevention Study - Evaluation of Hospital-Based Injury Recording and Community-Based Intervention for Traffic Injury Prevention. Accident Analysis and Prevention. 1995;27(1):111-23.

15. Asikainen I, Kaste M, Sarna S. Patients with traumatic brain injury referred to a rehabilitation and re-employment programme: Social and professional outcome for 508 Finnish patients 5 or more years after injury. Brain Injury. 1996;10(12):883-99.

16. Benjaminsen S, Gotzsche-Larsen K, Norrie B, Harder L, Luxhoi A. Patient violence in a psychiatric hospital in Denmark. Rate of violence and relation to diagnosis. Nordic Journal of Psychiatry. 1996;50(3):233-42.

17. Bjerneld H, Höglund C, Ellström B. A survey of registred traumas at the Mora hospital. The hospital size is not decisive when it comes to achievement. Läkartidningen. 1996;93(48):4412-4.

18. Brink O, Villadsen I, Davidsen MT, Petersen KK, Charles AV, Sabroe S. [Decline of violence in ]rhus. Violence registered in ]rhus hospital during a period of 12 years]. Ugeskrift for laeger. 1996;158(44):6277-81.

19. Gustafsson J, Lenninger K, Ribbe E, Andren-Sandberg Å. A prospective assessment of 1 270 trauma cases in Lund. A national registry may improve the quality. Läkartidningen. 1996;93(34):2857-9.

20. Hammer RR, Rooser B, Lidman D, Smeds S. Simplified external fixation for primary management of severe musculoskeletal injuries under war and peace time conditions. Journal of orthopaedic trauma. 1996;10(8):545-54.

21. Hansen HL. Surveillance of deaths on board Danish merchant ships, 1986-93: Implications for prevention. Occupational and Environmental Medicine. 1996;53(4):269-75.

22. Holmdahl L, Ortenwall P, Bunketorp O. Trauma grading for quality assurance of health care. [Swedish]. Lakartidningen. 1996;93(32-33):2739-42.

23. Hotvedt R, Kristiansen IS, Forde OH, Thoner J, Almdahl SM, Bjorsvik G, et al. Which groups of patients benefit from helicopter evacuation? Lancet. 1996;347(9012):1362-6.

24. Kopjar B, Guldvog B, Wiik J. Cost of medical treatment of injuries in Norway. Tidsskrift for den Norske Laegeforening. 1996;116(4):512-6.

25. Lindqvist K, Timpka T, Schelp L. Ten years of experiences from a participatory community-based injury prevention program in Motala, Sweden. Public Health. 1996;110(6):339-46.

26. Lindqvist KS, Brodin H. One-year economic consequences of accidents in a Swedish municipality. Accident Analysis and Prevention. 1996;28(2):209-19.

27. Linet MS, Nyren O, Gridley G, Adami HO, Buckland JD, McLaughlin JK, et al. Causes of death among patients surviving at least one year following splenectomy. American Journal of Surgery. 1996;172(4):320-3.

28. Madsen FQ, Hartmann-Andersen JF. [Fatal traffic accidents in Denmark. Survival time and factors of importance for the prehospital phase]. Ugeskrift for laeger. 1996;158(39):5432-7.

29. Madsen FQ, Hartmann-Andersen JF. Fatal traffic accidents in Denmark. Prehospital changes in consciousness and loss of life-years due to traffic accidents. [Danish]. Ugeskrift for laeger. 1996;158(39):5428-31.

30. Ortenwall P, Holmdahl L. A special emergency unit for severe cases of trauma established. Organization and experiences from Ostra hospital in Gothenburg. [Swedish]. Lakartidningen. 1996;93(32-33):2743, 6-7.

31. Ponzer S, Bergman B, Brismar B. Morbidity and injury recurrence in victims of firearm injuries. Public Health. 1996;110(1):41-6.

32. Printzlau A, Larsen CF, Kiaer T. Traumatology. Scoring systems and their use. [Danish]. Ugeskrift for laeger. 1996;158(43):6074-80.

33. Quebec Madsen F, Hartmann-Andersen JF. Deaths due to traffic accidents in Denmark. Survival time and factors of importance for the prehospital phase. Ugeskrift for Laeger. 1996;158(39):5432-7.

34. Quebec Madsen F, Hartmann-Andersen JF. Deaths due to traffic accidents in Denmark. Prehospital changes in consciousness and loss of life-years due to traffic accidents. Ugeskrift for Laeger. 1996;158(39):5428-31.

35. Solheim K. Changed injury pattern and by that therapeutic routines. Tidsskrift for den Norske Laegeforening. 1996;116(6):715.

36. Suominen P, Silfvast T, Korpela R, Erosuo J. Pediatric prehospital care provided by a physician-staffed emergency medical helicopter unit in Finland. Pediatric Emergency Care. 1996;12(3):169-72.

37. Tiika S, Bostman O, Marttinen E, Makitie I. A retrospective analysis of 36 civilian gunshot fractures. Journal of Trauma. 1996;40(3 SUPPL.):S212-S6.

38. Berg J, Johansen JG. [Penetrating head and neck gunshot injuries. A 10-year neurological material]. Tidsskrift for den Norske lageforening : tidsskrift for praktisk medicin, ny rakke. 1997;117(2):236-40.

39. Bostrom L. Injury panorama and medical consequences for 1158 persons assaulted in the central part of Stockholm, Sweden. Archives of Orthopaedic and Trauma Surgery. 1997;116(6-7):315-20.

40. Emanuelson I, vonWendt L. Epidemiology of traumatic brain injury in children and adolescents in south-western Sweden. Acta Paediatrica. 1997;86(7):730-5.

41. Holmdahl L, Ortenwall P. Causes and consequences of trauma in a Swedish county 1989-1992. European Journal of Surgery. 1997;163(2):83-92.

42. Ipsen T, Jorgsholm PB. [Fireworks-caused injuries in Denmark]. Ugeskrift for laeger. 1997;159(50):7492-4.

43. Jorgensen HS. [Hospital rehabilitation of patients with severe traumatic brain injury. I: Current status and organization in Denmark and abroad]. Ugeskrift for laeger. 1997;159(26):4089-92.

44. Jorgensen HS. [Hospital rehabilitation of patients with severe brain injuries. II: Prognosis and effect of early intensive, specialized rehabilitation]. Ugeskrift for laeger. 1997;159(26):4093-8.

45. Kuisma M, Alaspaa A. Out-of-hospital cardiac arrests of non-cardiac origin. Epidemiology and outcome. European Heart Journal. 1997;18(7):1122-8.

46. Makitie I, Paloneva H, Tikka S. Explosion injuries in Finland 1991-1995. Annales Chirurgiae Et Gynaecologiae. 1997;86(3):209-13.

47. Melhuus K, Sorensen K. [Violence 1994--Oslo Legevakt]. Tidsskrift for Den Norske Laegeforening. 1997;117(2):230-5.

48. Steen K, Hunskar S. Women and violence: A one-year survey from Bergen Accident and Emergency Department. Tidsskrift for den Norske Laegeforening. 1997;117(25):3640-2.

49. Steen K, Hunskar S. Violence in Bergen, Norway: A one-year prospective survey from Bergen Accident and Emergency Department. Tidsskrift for den Norske Laegeforening. 1997;117(2):226-9.

50. Steen K, Hunskar S. Violence in Bergen. A one-year material from the emergency department in Bergen. [Norwegian]. Tidsskrift for den Norske laegeforening. 1997;117(2):226-9.

51. Viano D, vonHolst H, Gordon E. Serious brain injury from traffic-related causes: Priorities for primary prevention. Accident Analysis and Prevention. 1997;29(6):811-6.

52. Ahlamaa-Tuompo J, Linna M. The socio-economic and demographic context of children's emergency injury visits in Helsinki in 1991-1994. European journal of emergency medicine : official journal of the European Society for Emergency Medicine. 1998;5(4):429-40.

53. Ahlamaa-Tuompo J, Linna M, Kekomaki M. Impact of user charges and socio-economic environment on visits to paediatric trauma unit in Finland. Scandinavian Journal of Social Medicine. 1998;26(4):265-9.

54. Asikainen I, Kaste M, Sarna S. Predicting late outcome for patients with traumatic brain injury referred to a rehabilitation programme: a study of 508 Finnish patients 5 years or more after injury. Brain Injury. 1998;12(2):95-107.

55. Brink O, Vesterby A, Jensen J. Pattern of injuries due to interpersonal violence. Injury. 1998;29(9):705-9.

56. Emanuelson I, v Wendt L. Medical management in south-west Sweden of children and adolescents with serious traumatic brain injury. Injury. 1998;29(3):193-8.

57. Emanuelson I, Wendt LV. Medical management in south-west Sweden of children and adolescents with serious traumatic brain injury. Injury. 1998;29(3):193-8.

58. Hansen TM, Hansen CM, Spangsberg NL, Christensen EF, Nielsen FF. Severely traumatized patients admitted to Aarhus Community Hospital 1994-1995. [Danish]. Ugeskrift for laeger. 1998;160(39):5640-4.

59. Hansen TM, Muff Hansen C, Spangsberg NL, Frischknecht Christensen E, Farsö Nielsen F. Severely traumatized patients admitted to Århus Municipal Hospital 1994-1995. Ugeskrift for Laeger. 1998;160(39):5640-4.

60. Ingebrigtsen T, Mortensen K, Romner B. The epidemiology of hospital-referred head injury in Northern Norway. Neuroepidemiology. 1998;17(3):139-46.

61. Lindqvist K, Timpka T, Schelp L, Ahlgren M. The WHO safe community program for injury prevention: evaluation of the impact on injury severity. Public Health. 1998;112(6):385-91.

62. Madsen CF. [Can injuries cause by train crash be prevented? Experiences from the train crash in Jelling in 1995]. Ugeskrift for laeger. 1998;160(49):7126-9.

63. Naredi S, Eden E, Zall S, Stephensen H, Rydenhag B. A standardized neurosurgical/neurointensive therapy directed toward vasogenic edema after severe traumatic brain injury: clinical results. Intensive Care Medicine. 1998;24(5):446-51.

64. Ponzer S, Sundquist J, Johansson SE, Bergman B. Gender, marital status and ethnicity. A Swedish retrospective study of criminality, morbidity and mortality among victims of non-fatal firearm injuries. Ethnicity & health. 1998;3(4):275-82.

65. Suominen P, Kivioja A, Ohman J, Korpela R, Rintala R, Olkkola KT. Severe and fatal childhood trauma. Injury-International Journal of the Care of the Injured. 1998;29(6):425-30.

66. Suominen P, Rasanen J, Kivioja A. Efficacy of cardiopulmonary resuscitation in pulseless paediatric trauma patients. Resuscitation. 1998;36(1):9-13.

67. Westgren N, Levi R. Quality of life and traumatic spinal cord injury. Archives of Physical Medicine and Rehabilitation. 1998;79(11):1433-9.

68. Ahlamaa-Tuompo J. The effect of user charges and socio-demographic environment on paediatric trauma hospitalisation in Helsinki in 1989-1994. European Journal of Epidemiology. 1999;15(2):133-9.

69. Alberts KA, Bellander BM, Modin G. Improved trauma care after reorganisation: A retrospective analysis. European Journal of Surgery. 1999;165(5):426-30.

70. Asikainen I, Kaste M, Sarna S. Early and late posttraumatic seizures in traumatic brain injury rehabilitation patients: Brain injury factors causing late seizures and influence of seizures on long-term outcome. Epilepsia. 1999;40(5):584-9.

71. Asikainen I, Nybo T, Muller K, Sarna S, Kaste M. Speed performance and long-term functional and vocational outcome in a group of young patients with moderate or severe traumatic brain injury. European Journal of Neurology. 1999;6(2):179-85.

72. Boman H, Bjornstig U, Hedelin A, Eriksson A. "Avoidable" deaths in two areas of Sweden - Analysis of deaths in hospital after injury. European Journal of Surgery. 1999;165(9):828-33.

73. Bostrom L, Nilsson B. A review of serious injury and death from gunshot wounds in Sweden: 1987 to 1994. European Journal of Surgery. 1999;165(10):930-6.

74. Engeland A, Kopjar B. Injuries among children. Tidsskrift for den Norske Laegeforening. 1999;119(6):784-7.

75. Engeland A, Kopjar B. Injuries among children treated at emergency medical centers and in hospitals 1990-97. Tidsskrift for den Norske Laegeforening. 1999;119(6):784-7.

76. Holstein BE, Due EP. [Injuries among 11-15 year old children]. Ugeskrift for laeger. 1999;161(35):4874-9.

77. Kannus P, Palvanen M, Niemi S, Parkkari J, Natri A, Vuori I, et al. Increasing number and incidence of fall-induced severe head injuries in older adults - Nationwide statistics in Finland in 1970-1995 and prediction for the future. American Journal of Epidemiology. 1999;149(2):143-50.

78. Kannus P, Parkkari J, Koskinen S, Niemi S, Palvanen M, Jarvinen M, et al. Fall-induced injuries and deaths among older adults. Jama-Journal of the American Medical Association. 1999;281(20):1895-9.

79. Kopjar B. Moped injuries among adolescents: a significant forgotten problem? Accident Analysis and Prevention. 1999;31(5):473-8.

80. Leppaniemi AK, Haapiainen RK. Risk factors of delayed diagnosis of pancreatic trauma. European Journal of Surgery. 1999;165(12):1134-7.

81. Mortensen K, Romner B, Ingebrigtsen T. Head injury in Troms. Tidsskrift for den Norske Laegeforening. 1999;119(13):1870-3.

82. Sjokvist P, Berggren L, Svantesson M, Nilstun T. Should the ventilator be withdrawn? Attitudes of the general public, nurses and physicians. European Journal of Anaesthesiology. 1999;16(8):526-33.

83. Skjoth-Rasmussen J, Rasmussen SW. [Injuries of bus passengers in an area of Copenhagen]. Ugeskrift for laeger. 1999;161(42):5803-6.

84. Timpka T, Lindqvist K, Schelp L, Ahlgren M. Community-based injury prevention: effects on health care utilization. International Journal of Epidemiology. 1999;28(3):502-8.

85. Wester T, Fevang LT, Wester K. Decompressive surgery in acute head injuries: Where should it be performed? Journal of Trauma-Injury Infection and Critical Care. 1999;46(5):914-9.

86. Wladis A, Bostrom L, Nilsson B. Unarmed violence-related injuries requiring hospitalization in Sweden from 1987 to 1994. Journal of Trauma-Injury Infection and Critical Care. 1999;47(4):733-7.

87. Alaranta H, Koskinen S, Leppanen L, Palomaki H. Nationwide epidemiology of hospitalized patients with first-time traumatic brain injury with special reference to prevention. Wiener medizinische Wochenschrift (1946). 2000;150(22):444-8.

88. Bjerre B, Schelp L. The community safety approach in Falun, Sweden - is it possible to characterise the most effective prevention endeavours and how long-lasting are the results? Accident Analysis and Prevention. 2000;32(3):461-70.

89. Bostrom L, Heinius G, Nilsson B. Trends in the incidence and severity of stab wounds in Sweden 1987-1994. European Journal of Surgery. 2000;166(10):765-70.

90. Brudvik C. Child injuries in Bergen, Norway. Injury-International Journal of the Care of the Injured. 2000;31(10):761-7.

91. Bunch EH. Delayed clarification: information, clarification and ethical decisions in critical care in Norway. Journal of advanced nursing. 2000;32(6):1485-91.

92. Ellsasser G, Berfenstam R. International comparisons of child injuries and prevention programs: recommendations for an improved prevention program in Germany. Injury prevention : journal of the International Society for Child and Adolescent Injury Prevention. 2000;6(1):41-5.

93. Gilbert M, Busund R, Skagseth A, Nilsen PA, Solbo JP. Resuscitation from accidental hypothermia of 13-7degreeC with circulatory arrest. Lancet. 2000;355(9201):375-6.

94. Hultling C, Giuliano F, Quirk F, Pena B, Mishra A, Smith MD. Quality of life in patients with spinal cord injury receiving Viagra (sildenafil citrate) for the treatment of erectile dysfunction. Spinal Cord. 2000;38(6):363-70.

95. Höylo T. Interdisciplinary training of emergency treatment in hospitals. Tidsskriftet Sykepleien. 2000;88(3):54-6.

96. Jensen TH, Pedersen B, Crone KL, Sarkandi HN, Juhl KH, Kristensen C, et al. [Emergency room patients and alcohol consumption]. Ugeskrift for laeger. 2000;162(43):5782-5.

97. Jousi M, Leppaniemi A. Management and outcome of traumatic aortic injuries. Annales Chirurgiae Et Gynaecologiae. 2000;89(2):89-92.

98. Kjeldsen SR. "Danger spots" for bicyclists based on data from accident registration in emergency departments. [Danish]. Ugeskrift for laeger. 2000;162(25):3602-5.

99. Lossius HM, Langhelle A, Pillgram-Larsen J, Lossius TA, Soreide E, Laake P, et al. Efficiency of activation of the trauma team in a Norwegian trauma referral centre. European Journal of Surgery. 2000;166(10):760-4.

100. Parkkari J, Kannus P, Niemi S, Koskinen S, Palvanen M, Vuori I, et al. Childhood deaths and injuries in Finland in 1971-1995. International Journal of Epidemiology. 2000;29(3):516-23.

101. Ponzer S, Nilsson M, Svensen C, Törnkvist H. Trauma registry is meaningful only for severe trauma. A year`s experience of trauma registration at Södersjukhuset. Läkartidningen. 2000;97(19):2320-2.

102. Rashid MA, Wikstrom T, Ortenwall P. Cardiac injuries: a ten-year experience. European Journal of Surgery. 2000;166(1):18-21.

103. Rashid MA, Wikstrom T, Ortenwall P. Outcome of lung trauma. European Journal of Surgery. 2000;166(1):22-8.

104. Suominen P, Baillie C, Kivioja A, Ohman J, Olkkola KT. Intubation and survival in severe paediatric blunt head injury. European journal of emergency medicine : official journal of the European Society for Emergency Medicine. 2000;7(1):3-7.

105. Wester T, Fevang LT, Wester K. Treatment of patients with acute head injury in Vestfold 1987-96. [Norwegian]. Tidsskrift for den Norske laegeforening. 2000;120(17):1960-3.

106. Yranheikki E, Savolainen H. Occupational safety and health in Finland. Journal of Safety Research. 2000;31(4):177-83.

107. Biering-Sorensen F, Haigh R, Holgersson MH, Ravnborg MH. [Use of outcome measures in physical medicine/rheumatological rehabilitation. Results of a questionnaire study]. Ugeskrift for laeger. 2001;163(5):612-6.

108. Bjorn Stolle L, Jorgensen B, Hvass I. Hurricane-related visits to the Emergency Department at Esbjerg Hospital on 3 December 1999. [Danish]. Ugeskrift for Laeger. 2001;163(49):6916-8.

109. Boesen MP, Larsen CF, Lippert FK, Larsen MS, Rock ND, Lang-Jensen T. [Admission, initial examination and care of severely injured in Denmark]. Ugeskrift for laeger. 2001;163(43):5963-6.

110. Bostrom L, Nilsson B. Pedestrians who required admission to hospital after collisions with motor vehicles in Sweden from 1987 to 1994. European Journal of Surgery. 2001;167(11):810-5.

111. Bostrom L, Nilsson B. A review of serious injuries and deaths from bicycle accidents in Sweden from 1987 to 1994. Journal of Trauma-Injury Infection and Critical Care. 2001;50(5):900-7.

112. Bostrom L, Wladis A, Nilsson B. A review of serious injuries and deaths among car occupants after motor vehicle crashes in Sweden from 1987 to 1994. Archives of Orthopaedic and Trauma Surgery. 2001;121(1-2):1-6.

113. Brattebo G, Wisborg T, Hoylo T. Organization of trauma admissions at Norwegian hospitals. [Norwegian]. Tidsskrift for den Norske laegeforening. 2001;121(20):2364-7.

114. Bylund PO, Bjornstig U. Use of seat belts in cars with different seat belt reminder systems. A study of injured car drivers. Annual proceedings / Association for the Advancement of Automotive Medicine Association for the Advancement of Automotive Medicine. 2001;45:1-9.

115. Ekelund M, Victorin A, Bergman O, Nilsson S, Lonroth H. [A case report: young man survived penetrating knife stabs to the heart and lung]. Lakartidningen. 2001;98(24):2936-8.

116. Ekman R, Welander G, Svanstrom L, Schelp L. Long-term effects of legislation and local promotion of child restraint use in motor vehicles in Sweden. Accident Analysis and Prevention. 2001;33(6):793-7.

117. Engberg AW, Petersen P. [A new regional service for rehabilitation after very serious brain injuries. I: Neurorehabilitation]. Ugeskrift for laeger. 2001;163(27):3755-6.

118. Engberg AW, Teasdale TW. Traumatic brain injury in Denmark 1979-1996. A national study of incidenceand mortality. European Journal of Epidemiology. 2001;17(5):437-42.

119. Hagen EM, Aarli JA, Gronning M. Patients with traumatic spinal cord injuries in a Norwegian university hospital, 1952-99. [Norwegian]. Tidsskrift for den Norske Laegeforening. 2001;121(28):3273-5.

120. Hasselberg M, Laflamme L, Weitoft GR. Socioeconomic differences in road traffic injuries during childhood and youth: a closer look at different kinds of road user. Journal of Epidemiology and Community Health. 2001;55(12):858-62.

121. Henriksson E, Ostrom M, Eriksson A. Preventability of vehicle-related fatalities. Accident Analysis and Prevention. 2001;33(4):467-75.

122. Holm C, Larsen MS, Houshian S, Torfing T. Ultrasonographic scanning of patients with severe abdominal injuries. [Danish]. Ugeskrift for laeger. 2001;163(43):5967-70.

123. Haagensen R, Sjoborg KA, Rossing A, Ingilae H, Markengbakken L, Steen PA. [Rescue operations with helicopter ambulances in the Barents sea]. Tidsskrift for den Norske lageforening : tidsskrift for praktisk medicin, ny rakke. 2001;121(9):1070-4.

124. Johansson L, Eriksson A, Bjornstig U. Teenager injury panorama in northern Sweden. International journal of circumpolar health. 2001;60(3):380-90.

125. Kannus P, Niemi S, Parkkari J, Palvanen M. Epidemiology of adulthood injuries: A quickly changing injury profile in Finland. Journal of Clinical Epidemiology. 2001;54(6):597-602.

126. Lippert F, Svensson LB. Trauma centers and trauma systems - a new development. Medicinsk Årbog. 2001:161-72.

127. Lossius HM, Langhelle A, Sreide E, Pillgram-Larsen J, Lossius TA, Laake P, et al. Reporting data following major trauma and analysing factors associated with outcome using the new Utstein style recommendations. Resuscitation. 2001;50(3):263-72.

128. Rashid MA. Thoracic vascular injuries: A major problem in trauma. Scandinavian Cardiovascular Journal. 2001;35(4):285-7.

129. Rashid MA, Ortenwall P, Wikstrom T. Cardiovascular injuries associated with sternal fractures. European Journal of Surgery. 2001;167(4):243-8.

130. Sjolingstad A, Alvaer K, Engeland A, Forsen L. [Registration of injuries with the help of ICD-10 at Norwegian hospitals]. Tidsskrift for den Norske lageforening : tidsskrift for praktisk medicin, ny rakke. 2001;121(9):1052-4.

131. Streng M, Tikka S, Leppaniemi A. Assessing the severity of truncal gunshot wounds: A nation-wide analysis from Finland. Annales Chirurgiae Et Gynaecologiae. 2001;90(4):246-51.

132. Teasdale TW, Engberg AW. Suicide after traumatic brain injury: a population study. Journal of Neurology Neurosurgery and Psychiatry. 2001;71(4):436-40.

133. Vesterbacka J, Eriksson A. A rural ambulance helicopter system in northern Sweden. Air Medical Journal. 2001;20(3):28-31.

134. Ytterstad B, Norheim AJ. The epidemiology of injuries in Svalbard compared with Harstad. International journal of circumpolar health. 2001;60(2):184-95.

135. Ytterstad B, Norheim J. Snowmobile injuries in Svalbard--a three year study [corrected]. International journal of circumpolar health. 2001;60(4):685-95.

136. Bostrom L, Nilsson B. [Marked injury decline among pedestrians 1987-1994]. Lakartidningen. 2002;99(28-29):3018-20.

137. Bostrom L, Wladis A, Nilsson B. Injured moped riders who required admission to hospital in Sweden from 1987 to 1994. European Journal of Surgery. 2002;168(6):360-5.

138. Brink O, Bitch O, Petersen KK, Charles AV. [Violence in Aarhus over two decades]. Ugeskrift for laeger. 2002;164(8):1044-8.

139. Brink O, Bitch O, Petersen KK, Charles AV. Two decades of violence - A cohort study from the Danish municipality of Aarhus. Danish Medical Bulletin. 2002;49(1):64-7.

140. Elf K, Nilsson P, Enblad P. Outcome after traumatic brain injury improved by an organized secondary insult program and standardized neurointensive care. Critical Care Medicine. 2002;30(9):2129-34.

141. Engstrom K, Ekman R, Welander G, Laflamme L. Area-based differences in injury risks in a small Swedish municipality--Geographic and social differences. Injury control and safety promotion. 2002;9(1):53-7.

142. Faergemann C, Larsen LB. Severe injuries following road traffic accidents with motor vehicles in the Municipality of Odense in the years 1990 to 1999. Ugeskrift for Laeger. 2002;164(44):5126-30.

143. Faergemann C, Mikkelsen JB, Wedderkopp N, Skov O. [Injured elderly victims of bag-snatching treated at Odense University Hospital 1996-2000]. Ugeskrift for laeger. 2002;164(21):2768-71.

144. Hansen TM, Hoyer CC, Laustrup TK, Landsfeldt US, Christensen EF. [Seriously injured patients intubated at the accident site. A three-year survey from the mobile emergency care unit in Aarhus]. Ugeskrift for Laeger. 2002;164(44):5123-6.

145. Houshian S, Larsen MS, Holm C. Missed injuries in a level I trauma center. Journal of Trauma-Injury Infection and Critical Care. 2002;52(4):715-9.

146. Kurola J, Wangel M, Uusaro A, Ruokonen E. Paramedic helicopter emergency service in rural Finland - do benefits justify the cost? Acta Anaesthesiologica Scandinavica. 2002;46(7):779-84.

147. Larsen LB. [The importance of the use of bicycle helmets for head injuries among injured bicyclists aged 0-15 years]. Ugeskrift for laeger. 2002;164(44):5115-9.

148. Larsen MS, Houshian S, Mikkelsen JB. Evaluation of multitraumatised patients admitted to a trauma centre. [Danish]. Ugeskrift for Laeger. 2002;164(44):5130-4.

149. Lauritsen JM, Kidholm K, Skov O, Norgard L. [Economic coverage and average costs related to injuries treated in a hospital]. Ugeskrift for laeger. 2002;164(44):5107-12.

150. Laursen B, Moller H, Frimodt-Moller B. Bicycle accidents: Differences between one-vehicle accidents and accidents involving two or more vehicles. [Danish]. Ugeskrift for Laeger. 2002;164(44):5112-5.

151. Lindqvist K. Economic impact of injuries according to type of injury. Croatian Medical Journal. 2002;43(4):386-9.

152. Lings S. [Increased frequency of driving accidents in epilepsy]. Ugeskrift for laeger. 2002;164(14):1920-2.

153. Lings S. Increased frequency of driving accidents among patients with multiple sclerosis. [Danish]. Ugeskrift for Laeger. 2002;164(44):5134-7.

154. Makitie I, Pihlajamaki H. Fatal firearm injuries in Finland: a nationwide survey. Scandinavian journal of surgery : SJS : official organ for the Finnish Surgical Society and the Scandinavian Surgical Society. 2002;91(4):328-31.

155. Nielsen EW, Ulvik A, Carlsen AW, Rannestad B. When is an anesthesiologist needed in a helicopter emergency medical service in northern Norway? Acta Anaesthesiologica Scandinavica. 2002;46(7):785-8.

156. Nieminen S, Lehtonen O-P, Linna M. Population density and occurrence of accidents in Finland. Prehospital and disaster medicine. 2002;17(4):206-8.

157. Schultz Larsen M, Houshian S, Beck Mikkelsen J. Evaluation of multitraumatized patients admitted to a trauma center. Ugeskrift for Laeger. 2002;164(42):5130-4.

158. Steen K, Hunskaar S. Medical consequences of violence: a two-year prospective study from a primary care accident and emergency department in Norway. Scandinavian Journal of Primary Health Care. 2002;20(2):108-12.

159. Timonen M, Miettunen J, Hakko H, Zitting P, Veijola J, von Wendt L, et al. The association of preceding traumatic brain injury with mental disorders, alcoholism and criminality: the Northern Finland 1966 Birth Cohort Study. Psychiatry Research. 2002;113(3):217-26.

160. Ulvik A, Rannestad BS, Carlsen AW, Nielsen EW. Emergency medical service with a rescue helicopter in Northern Norway. [Norwegian]. Tidsskrift for den Norske Laegeforening. 2002;122(1):25-9.

161. Ulvik A, Rannestad BS, Carlsen AW, Nielsen EW. [Rescue helicopter service in Bodo--advanced emergency service or alternative transportation?]. Tidsskrift for Den Norske Laegeforening. 2002;122(1):25-9.

162. Uranus S, Lennquist S. Trauma management and education in Europe: A survey of twelve geographically and socioeconomically diverse European countries. European Journal of Surgery. 2002;168(12):730-5.

163. Weitoft GR, Haglund B, Hjern A, Rosen M. Mortality, severe morbidity and injury among long-term lone mothers in Sweden. International Journal of Epidemiology. 2002;31(3):573-80.

164. Wladis A, Bostrom L, Nilsson B. Injuries in 8927 patients admitted after motor-cycle crashes in Sweden 1987-1994 inclusive. European Journal of Surgery. 2002;168(3):187-92.

165. Andersson EH, Bjorklund R, Emanuelson I, Stalhammar D. Epidemiology of traumatic brain injury: a population based study in western Sweden. Acta Neurologica Scandinavica. 2003;107(4):256-9.

166. Augutis M, Levi R. Pediatric spinal cord injury in Sweden: Incidence, etiology and outcome. Spinal Cord. 2003;41(6):328-36.

167. Christensen EF, Hoyer CC. [Prehospital endotracheal intubation of critically injured patients]. Ugeskrift for laeger. 2003;165(45):4299-301.

168. Christensen EF, Laustrup TK, Hoyer CC, Hougaard K, Spangsberg NL. [Mortality among severely injured patients before and after establishment of a trauma center in Aarhus]. Ugeskrift for laeger. 2003;165(45):4296-9.

169. de Groot PC, Hjeltnes N, Heijboer AC, Stal W, Birkeland K. Effect of training intensity on physical capacity, lipid profile and insulin sensitivity in early rehabilitation of spinal cord injured individuals. Spinal Cord. 2003;41(12):673-9.

170. Ebeling H, Vuokko A, Moilanen I. Traumatic injuries of children and adolescents - are they associated with psychiatric contacts? Nordic Journal of Psychiatry. 2003;57(5):345-50.

171. Emanuelson I, von Wendt L, Hagberg I, Marchioni-Johansson M, Ekberg G, Olsson U, et al. Early community outreach intervention in children with acquired brain injury. International Journal of Rehabilitation Research. 2003;26(4):257-64.

172. Engstrom K, Laflamme L, Diderichsen F. Equalisation of socioeconomic differences in injury risks at school age? A study of three age cohorts of Swedish children and adolescents. Social Science & Medicine. 2003;57(10):1891-9.

173. Franzen L, Ortenwall P, Backteman T. Major trauma with multiple injuries in Swedish children. The European journal of surgery Supplement : = Acta chirurgica Supplement. 2003(588):3-7.

174. Gravseth HM, Lund J, Wergeland E. [Occupational injuries in Oslo: a study of occupational injuries treated by the Oslo Emergency Ward and Oslo Ambulance Service]. Tidsskrift for den Norske lageforening : tidsskrift for praktisk medicin, ny rakke. 2003;123(15):2060-4.

175. Gravseth HM, Wergeland E, Lund J. [Underreporting of occupational injuries to the Labour Inspection]. Tidsskrift for den Norske lageforening : tidsskrift for praktisk medicin, ny rakke. 2003;123(15):2057-9.

176. Groot PC, Hjeltnes N, Heijboer AC, Stal W, Birkeland K. Effect of training intensity on physical capacity, lipid profile and insulin sensitivity in early rehabilitation of spinal cord injured individuals. Spinal cord [Internet]. 2003; 41(12):[673-9 pp.]. Available from: http://onlinelibrary.wiley.com/o/cochrane/clcentral/articles/374/CN-00559374/frame.html

http://www.nature.com/sc/journal/v41/n12/pdf/3101534a.pdf.

177. Hagglund M, Walden M, Ekstrand J. Exposure and injury risk in Swedish elite football: a comparison between seasons 1982 and 2001. Scandinavian Journal of Medicine & Science in Sports. 2003;13(6):364-70.

178. Halvorsrud R, Hagen S, Fagernes S, Mjelstad S, Romundstad L. Trauma team training in a distributed virtual emergency room. Studies in health technology and informatics. 2003;94:100-2.

179. Hoyer CC, Christensen EF. [Prehospital recording of vital parameters in injured patients]. Ugeskrift for laeger. 2003;165(45):4285-7.

180. Lysberg K, Severinsson E. Spinal cord injured women's views of sexuality: a Norwegian survey. Rehabil Nurs. 2003;28(1):23-6.

181. Maraste P, Persson U, Berntman M. Long-term follow-up and consequences for severe road traffic injuries - treatment costs and health impairment in Sweden in the 1960s and the 1990s. Health Policy. 2003;66(2):147-58.

182. Rake EL. Emergency management and decision making on accident scenes: Taxonomy, models and future research. International Journal of Emergency Management. 2003;1(4):397-409.

183. Rashid MA, Lund JT. Trauma to the heart and thoracic aorta: The Copenhagen experience. Interactive Cardiovascular and Thoracic Surgery. 2003;2(1):53-7.

184. Rasmussen K, Carstensen O, Lauritsen JM, Glasscock DJ, Hansen ON, Jensen UF. Prevention of farm injuries in Denmark. Scandinavian Journal of Work, Environment and Health. 2003;29(4):288-96.

185. Sollid S, Munch-Ellingsen J, Gilbert M, Ingebrigtsen T. Pre- and inter-hospital transport of severely head-injured patients in rural Northern Norway. Journal of Neurotrauma. 2003;20(3):309-14.

186. van't Hooft I, Andersson K, Sejersen T, Bartfai A, von Wendt L. Attention and memory training in children with acquired brain injuries. Acta Paediatrica. 2003;92(8):935-40.

187. Wisborg T, Hoylo T, Siem G. Death after injury in rural Norway: high rate of mortality and prehospital death. Acta Anaesthesiologica Scandinavica. 2003;47(2):153-6.

188. Wisborg T, Ronning TH, Beck VB, Brattebo G. Preparing teams for low-frequency emergencies in Norwegian hospitals. Acta Anaesthesiologica Scandinavica. 2003;47(10):1248-50.

189. Wladis A, Bostrom L, Nilsson B. [Injuries and mortality in motorcycle and moped accidents in Sweden 1987-1994. Advanced age and male sex are risk factors of fatal moped and motorcycle accidents]. Lakartidningen. 2003;100(14):1238-41.

190. Aare M, von Holst H. Injuries from motorcycle- and moped crashes in Sweden from 1987 to 1999. Injury control and safety promotion. 2003;10(3):131-8.

191. Biering-Sorensen F, Hansen RB, Biering-Sorensen J. Mobility aids and transport possibilities 10-45 years after spinal cord injury. Spinal Cord. 2004;42(12):699-706.

192. Dahlberg A, Perttila I, Wuokko E, Ala-Opas M. Bladder management in persons with spinal cord lesion. Spinal Cord. 2004;42(12):694-8.

193. Ekeus C, Christensson K, Hjern A. Unintentional and violent injuries among pre-school children of teenage mothers in Sweden: a national cohort study. Journal of Epidemiology and Community Health. 2004;58(8):680-5.

194. Ekstrand J, Walden M, Hagglund M. Risk for injury when playing in a national football team. Scandinavian Journal of Medicine & Science in Sports. 2004;14(1):34-8.

195. Engberg AW, Teasdale TW. A population-based study of survival and discharge status for survivors after head injury. Acta Neurologica Scandinavica. 2004;110(5):281-90.

196. Haagensen R, Sjoborg K-A, Rossing A, Ingilae H, Markengbakken L, Steen P-A. Long-range rescue helicopter missions in the Arctic. Prehospital and disaster medicine. 2004;19(2):158-63.

197. Ingemann-Hansen O, Brink O. City centre violence. Journal of Clinical Forensic Medicine. 2004;11(6):303-7.

198. Jansson B, Stenbacka M, Leifman A, Romelsjo A. A small fraction of patients with repetitive injuries account for a large portion of medical costs. European Journal of Public Health. 2004;14(2):161-7.

199. Laflamme L, Engstrom K, Huisman M. Is there equalisation in socioeconomic differences in the risk of traffic injuries in childhood? A study of three cohorts of Swedish school children. International journal of adolescent medicine and health. 2004;16(3):253-63.

200. Lund J, Bjerkedal T, Gravseth HM, Vilimas K, Wergeland E. A two-step medically based injury surveillance system - experiences from the Oslo injury register. Accident Analysis and Prevention. 2004;36(6):1003-17.

201. Norrbrink Budh C, Lundeberg T. Non-pharmacological pain-relieving therapies in individuals with spinal cord injury: a patient perspective. Complement Ther Med. 2004;12(4):189-97.

202. Olsson J, Svensen C. The use of hypertonic solutions in prehospital care in Scandinavia. Akuttjournalen. 2004;21(2):78-85.

203. Ostergaard HT, Ostergaard D, Lippert A. Implementation of team training in medical education in Denmark. Quality and Safety in Health Care. 2004;13(SUPPL. 1):i91-i5.

204. Persson G. Are specially equiped ambulances necessary in prehospital emergency care? A study of the significance of emergency ambulances for the survival of patients after Prio-1 alarm in Jönköping during 1997-2001. Akuttjournalen. 2004;12(3):157-61.

205. Rognås LK, Hansen PB, Lippert FK, Trier Möller J. Medical emergency calls and ambulance dispatch in Copenhagen. Ugeskrift for Laeger. 2004;166(13):1229-32.

206. Ruter A, Nilsson H, Wikström T. Prehospital management leadership - conceptual training and certification. Akuttjournalen. 2004;12(3):148-9.

207. Skak C, Rasmussen LS, Moller JT. [Transfer ambulance by Copenhagen Hospital Cooperation--a status report]. Ugeskrift for laeger. 2004;166(36):3084-5.

208. Steen K, Hunskaar S. Gender and physical violence. Social Science & Medicine. 2004;59(3):567-71.

209. Stormo A, Sollid S, Stormer J, Ingebrigtsen T. Neurosurgical teleconsultations in northern Norway. Journal of Telemedicine and Telecare. 2004;10(3):135-9.

210. Dahlberg A, Kotila M, Leppanen P, Kautiainen H, Alaranta H. Prevalence of spinal cord injury in Helsinki. Spinal Cord. 2005;43(1):47-50.

211. Ekman R, Svanstrom L, Langberg B. Temporal trends, gender, and geographic distributions in child and youth injury rates in Sweden. Injury Prevention. 2005;11(1):29-32.

212. Elfstrom ML, Ryden A, Kreuter M, Taft C, Sullivan M. Relations between coping strategies and health-related quality of life in patients with spinal cord lesion. Journal of Rehabilitation Medicine. 2005;37(1):9-16.

213. Gotschalck Sunesen K, Pallesen J, Koefoed-Nielsen J, Frischknecht Christensen E. From accident to trauma center - the lapse of time for patients with severe head injuries. Ugeskrift for Laeger. 2005;167(36):3397-400.

214. Gaarder C, Skaga NO, Eken T, Pillgram-Larsen J, Buanes T, Naess PA. The impact of patient volume on surgical trauma training in a Scandinavian trauma centre. Injury-International Journal of the Care of the Injured. 2005;36(11):1288-92.

215. Hagen EM, Aarli JA, Gronning M. The clinical significance of spinal cord injuries in patients older than 60 years of age. Acta Neurologica Scandinavica. 2005;112(1):42-7.

216. Hillman J, Aneman O, Anderson C, Sjogren F, Saberg C, Mellergard P. A microdialysis technique for routine measurement of macromolecules in the injured human brain. Neurosurgery. 2005;56(6):1264-8.

217. Howells T, Elf K, Jones PA, Ronne-Engstrom E, Piper I, Nilsson P, et al. Pressure reactivity as a guide in the treatment of cerebral perfusion pressure in patients with brain trauma. Journal of Neurosurgery. 2005;102(2):311-7.

218. Jeppesen E, Wisborg T. [ISnowmobiles and serious injuries in Western Finnmark]. Tidsskrift for den Norske lageforening : tidsskrift for praktisk medicin, ny rakke. 2005;125(23):3248-51.

219. Jeppesen E, Wisborg T. Increased number of snowmobiles and serious injuries in Western Finnmark, Norway. [Norwegian, English]. Tidsskrift for den Norske Laegeforening. 2005;125(23):3248-51.

220. Johansson L, Stenlund H, Lindqvist P, Eriksson A. A survey of teenager unnatural deaths in northern Sweden 1981-2000. Accident Analysis and Prevention. 2005;37(2):253-8.

221. Laursen B, Frimodt-Moller B. [Socioeconomic differences in accident-related health care]. Ugeskrift for laeger. 2005;167(17):1855-8.

222. Lundälv J. Emergency medical vehicle collisions (EMVC) seen from an international perspective - a literature review of the phenomenom EMVC and a knowledge review of interest for the injury registration within the EMVC field in Sweden during 1990-2005. Akuttjournalen. 2005;13(3):148-58.

223. Mattila VM, Parkkari J, Niemi S, Kannus P. Injury-related deaths among Finnish adolescents in 1971-2002. Injury. 2005;36(9):1016-21.

224. Nordseth T, Skogvoll E, Gisvold SE. Emergency in-hospital anaesthesia assistance. [Norwegian]. Tidsskrift for den Norske Laegeforening. 2005;125(22):3124-6.

225. Polinder S, Meerding WJ, van Baar ME, Toet H, Mulder S, van Beeck EF. Cost estimation of injury-related hospital admissions in 10 European countries. Journal of Trauma-Injury Infection and Critical Care. 2005;59(6):1283-90.

226. Sarajuuri JM, Kaipio ML, Koskinen SK, Niemela MR, Servo AR, Vilkki JS. Outcome of a comprehensive neurorehabilitation program for patients with traumatic brain injury. Archives of Physical Medicine and Rehabilitation. 2005;86(12):2296-302.

227. Skoglund TS, Nellgard B. Long-time outcome after transient transtentorial herniation in patients with traumatic brain injury. Acta Anaesthesiologica Scandinavica. 2005;49(3):337-40.

228. Sundstrom T, Sollid S, Wester K. [Deaths from traumatic brain injury in the Nordic countries, 1987-2000]. Tidsskrift for den Norske lageforening : tidsskrift for praktisk medicin, ny rakke. 2005;125(10):1310-2.

229. Sunesen KG, Pallesen J, Kofoed-Nielsen J, Christensen EF. [From accident to trauma center--the lapse of time for patients with severe head injuries]. Ugeskrift for Laeger. 2005;167(36):3397-400.

230. Talving P, Palstedt J, Riddez L. Prehospital management and fluid resuscitation in hypotensive trauma patients admitted to Karolinska University Hospital in Stockholm. Prehospital and disaster medicine. 2005;20(4):228-34.

231. Teasdale TW, Engberg AW. Subjective well-being and quality of life following traumatic brain injury in adults: A long-term population-based follow-up. Brain Injury. 2005;19(12):1041-8.

232. Vester AE, Christensen EF, Andersen SK, Tonnesen E. Ethical and practical problems in blood sampling for research purposes during pre-hospital emergencies. Acta Anaesthesiologica Scandinavica. 2005;49(10):1540-3.

233. Wahlstrom MR, Olivecrona M, Koskinen LO, Rydenhag B, Naredi S. Severe traumatic brain injury in pediatric patients: treatment and outcome using an intracranial pressure targeted therapy--the Lund concept. Intensive Care Medicine. 2005;31(6):832-9.

234. Ytterstad B, Dahlberg T. A five-year study of snowmobile injuries in Svalbard: Does prevention work?. [Norwegian, English]. Tidsskrift for den Norske Laegeforening. 2005;125(23):3252-5.

235. Ala-Kokko T, Ohtonen P, Laurila J, Martikainen M, Kaukoranta P. Development of renal failure during the initial 24 h of intensive care unit stay correlates with hospital mortality in trauma patients. Acta Anaesthesiologica Scandinavica. 2006;50(7):828-32.

236. Albertsson P, Falkmer T, Kirk A, Mayrhofer E, Bjornstig U. Case study: 128 Injured in rollover coach crashes in Sweden - Injury outcome, mechanisms and possible effects of seat belts. Safety Science. 2006;44(2):87-109.

237. Augutis M, Abel R, Levi R. Pediatric spinal cord injury in a subset of European countries. Spinal Cord. 2006;44(2):106-12.

238. Bergstrom AL, Samuelsson K. Evaluation of manual wheelchairs by individuals with spinal cord injuries. Disability and rehabilitation. 2006;Assistive technology. 1(3):175-82.

239. Busch M. Portable ultrasound in pre-hospital emergencies: a feasibility study. Acta Anaesthesiologica Scandinavica. 2006;50(6):754-8.

240. Clemmesen ML, Rytter S, Birch K, Lindholt JS, Jensen SS, Troelsen S. [Should high-energy traumas always result in a trauma team call?]. Ugeskrift for laeger. 2006;168(35):2916-20.

241. Engberg AW, Liebach A, Nordenbo A. Centralized rehabilitation after severe traumatic brain injury - a population-based study. Acta Neurologica Scandinavica. 2006;113(3):178-84.

242. Estrada F. Trends in violence in Scandinavia according to different indicators - An exemplification of the value of Swedish hospital data. British Journal of Criminology. 2006;46(3):486-504.

243. Franzen C, Bjornstig U, Jansson L. Injured in traffic: experiences of care and rehabilitation. Accident and emergency nursing. 2006;14(2):104-10.

244. Gravseth HM, Lund J, Wergeland E. [Risk factors for accidental injuries in the construction industry]. Tidsskrift for den Norske lageforening : tidsskrift for praktisk medicin, ny rakke. 2006;126(4):453-6.

245. Handolin L, Leppaniemi A, Vihtonen K, Lakovaara M, Lindahl J. Finnish Trauma Audit 2004: Current state of trauma management in Finnish hospitals. Injury. 2006;37(7):622-5.

246. Himanen L, Portin R, Isoniemi H, Helenius H, Kurki T, Tenovuo O. Longitudinal cognitive changes in traumatic brain injury - A 30-year follow-up study. Neurology. 2006;66(2):187-92.

247. Hoyer CC, Christensen EF, Andersen NT. On-scene time in advanced trauma life support by anaesthesiologists. European Journal of Emergency Medicine. 2006;13(3):156-9.

248. Iirola TT, Laaksonen MI, Vahlberg TJ, Palve HK. Effect of physician-staffed helicopter emergency medical service on blunt trauma patient survival and prehospital care. European Journal of Emergency Medicine. 2006;13(6):335-9.

249. Isaksen MI, Wisborg T, Brattebo G. [Organisation of trauma services--major improvements over four years]. Tidsskrift for den Norske lageforening : tidsskrift for praktisk medicin, ny rakke. 2006;126(2):145-7.

250. Kinnunen I, Aitasalo K. A review of 59 consecutive patients with lesions of the anterior cranial base operated on using the subcranial approach. Journal of Cranio-Maxillofacial Surgery. 2006;34(7):405-11.

251. Kruger AJ, Hesselberg N, Abrahamsen GT, Bartnes K. When should the trauma team be activated?. [Norwegian]. Tidsskrift for den Norske Laegeforening. 2006;126(10):1335-7.

252. Laursen B. Injury patterns in children with frequent emergency department visits. Emergency Medicine Journal. 2006;23(1):59-60.

253. Lundgren-Nilsson A, Tennant A, Grimby G, Sunnerhagen KS. Cross-diagnostic validity in a generic instrument: an example from the Functional Independence Measure in Scandinavia. Health Qual Life Outcomes. 2006;4:55.

254. Makitie I, Mattila VM, Pihlajamaki H. Severe vascular gunshot injuries of the extremities: A ten-year nation-wide analysis from Finland. Scandinavian Journal of Surgery. 2006;95(1):49-54.

255. Mattila VM, Makitie I, Pihlajamaki H. Trends in hospitalization for firearm-related injury in Finland from 1990 to 2003. Journal of Trauma-Injury Infection and Critical Care. 2006;61(5):1222-7.

256. Mattila VM, Parkkari J, Korpela H, Pihlajamaki H. Hospitalisation for injuries among Finnish conscripts in 1990-1999. Accident Analysis and Prevention. 2006;38(1):99-104.

257. Nordqvist C, Holmqvist M, Nilsen P, Bendtsen P, Lindqvist K. Usual drinking patterns and non-fatal injury among patients seeking emergency care. Public Health. 2006;120(11):1064-73.

258. Nysted M, Drogset JO. Trampoline injuries. British Journal of Sports Medicine. 2006;40(12):984-7.

259. Pakarinen TK, Lepaniemi A, Hiltunen KM, Salo J. Management of cervical stab wounds in low volume trauma centres: Systematic physical examination and low threshold for adjunctive studies, or surgical exploration. Injury-International Journal of the Care of the Injured. 2006;37(5):440-7.

260. Ringdal M, Johansson L, Lundberg D, Bergbom I. Delusional memories from the intensive care unit-Experienced by patients with physical trauma. Intensive and Critical Care Nursing. 2006;22(6):346-54.

261. Skaga NO, Eken T, Steen PA. Assessing quality of care in a trauma referral center: Benchmarking performance by TRISS-based statistics or by analysis of stratified ISS data? Journal of Trauma-Injury Infection and Critical Care. 2006;60(3):538-47.

262. Sulheim S, Holme I, Ekeland A, Bahr R. Helmet use and risk of head injuries in alpine skiers and snowboarders. Jama-Journal of the American Medical Association. 2006;295(8):919-24.

263. Tagliaferri F, Compagnone C, Korsic M, Servadei F, Kraus J. A systematic review of brain injury epidemiology in Europe. Acta Neurochirurgica. 2006;148(3):255-67.

264. Winqvist S, Jokelainen J, Luukinen H, Hillbom M. Adolescents' drinking habits predict later occurrence of traumatic brain injury: 35-year follow-up of the northern Finland 1966 birth cohort. The Journal of adolescent health : official publication of the Society for Adolescent Medicine. 2006;39(2):275.e1-7.

265. Wisborg T, Brattebo G, Brattebo J, Brinchmann-Hansen A. Training multiprofessional trauma teams in Norwegian hospitals using simple and low cost local simulations. Education for health (Abingdon, England). 2006;19(1):85-95.

266. Zambon F, Hasselberg M. Factors affecting the severity of injuries among young motorcyclists - A Swedish nationwide cohort study. Traffic Injury Prevention. 2006;7(2):143-9.

267. Andersson SO, Dahlgren LO, Lundberg L, Sjostrom B. The criteria nurses use in assessing acute trauma in military emergency care. Accident and Emergency Nursing. 2007;15(3):148-56.

268. Bach A, Christensen EF. Accuracy in identifying patients with loss of consciousness in a police-operated emergency call centre - First step in the chain of survival. Acta Anaesthesiologica Scandinavica. 2007;51(6):742-6.

269. Blicher JU, Jensen CB, Westh J, Hellemann I. Housing and work outcomes one year after severe brain injury: An interview-based follow-up study. [Danish]. Ugeskrift for Laeger. 2007;169(3):228-31.

270. Bylund PO, Wretstrand A, Falkmer T, Lovgren A, Petzall J. Injuries in special transportation services for elderly and disabled--a multi-methodology approach to estimate incidence and societal costs. Traffic injury prevention. 2007;8(2):180-8.

271. De Leon AP, Svanstrom L, Welander G, Schelp L, Santesson P, Ekman R. Differences in child injury hospitalizations in Sweden: the use of time-trend analysis to compare various community injury-prevention approaches. Scandinavian Journal of Public Health. 2007;35(6):623-30.

272. Engberg AW, Penninga EI, Teasdale TW. [Nordic accident classification system used in the Danish National Hospital Registration System to register causes of severe traumatic brain injury]. Ugeskrift for laeger. 2007;169(45):3856-60.

273. Engberg AW, Teasdale TW. Epidemiology and treatment of head injuries in Denmark 1994-2002, illustrated with hospital statistics. [Danish]. Ugeskrift for Laeger. 2007;169(3):199-203.

274. Faergemann C, Lauritsen JM, Brink O, Skov O. Trends in deliberate interpersonal violence in the Odense Municipality, Denmark 1991-2002. The Odense study on deliberate interpersonal violence. Journal of forensic and legal medicine. 2007;14(1):20-6.

275. Faergemann C, Lauritsen JM, Brink O, Stovring H. The epidemiology of repeat contacts with an Emergency Department or an Institute of Forensic Medicine due to violent victimization in a Danish urban population. Journal of forensic and legal medicine. 2007;14(6):333-9.

276. Falk AC. Current incidence and management of children with traumatic head injuries: The Stockholm experience. Developmental Neurorehabilitation. 2007;10(1):49-55.

277. Franzen L, Ortenwall P, Backteman T. Children in Sweden admitted to intensive care after trauma. Injury-International Journal of the Care of the Injured. 2007;38(1):91-7.

278. Gaarder C, Naess PA, Eken T, Skaga NO, Pillgram-Larsen J, Klow NE, et al. Liver injuries-Improved results with a formal protocol including angiography. Injury. 2007;38(9):1075-83.

279. Halldorsson JG, Flekkoy KM, Gudmundsson KR, Arnkelsson GB, Arnarson EO. Urban-rural differences in pediatric traumatic head injuries: A prospective nationwide study. Neuropsychiatric Disease and Treatment. 2007;3(6):935-41.

280. Handolin L, Lindahl J, Lakovaara M, Vihtonen K, Leppaniemi A. Towards regionalized care of severe orthopedic injuries: A survey on non-university hospitals in Finland. European Journal of Trauma and Emergency Surgery. 2007;33(2):183-7.

281. Hannerz H, Mikkelsen KL, Nielsen ML, Tuchsen F, Spangenberg S. Social inequalities in injury occurrence and in disability retirement attributable to injuries: a 5 year follow-up study of a 2.1 million gainfully employed people. Bmc Public Health. 2007;7.

282. Hansen KS, Uggen PE, Brattebo G, Wisborg T. Training operating room teams in damage control surgery for trauma: A Followup study of the Norwegian model. Journal of the American College of Surgeons. 2007;205(5):712-6.

283. Herttua K, Makela P, Martikainen P. Differential trends in alcohol-related mortality: A register-based follow-up study in Finland in 1987-2003. Alcohol and Alcoholism. 2007;42(5):456-64.

284. Jacobsson LJ, Westerberg M, Lexell J. Demographics, injury characteristics and outcome of traumatic brain injuries in northern Sweden. Acta Neurologica Scandinavica. 2007;116(5):300-6.

285. Johansson A, Andersson S, Persson M-L. A psychiatric and social matched case series comparison of victims of criminal homicide and homicide perpetrators in Sweden. Nordic Journal of Psychiatry. 2007;61(6):427-32.

286. Johansson A, Oden A, Dahlgren L-O, Sjostrom B. A comparison of experiences of training emergency care in military exercises and competences among conscript nurses with different levels of education. Military medicine. 2007;172(10):1046-52.

287. Kann SH, Hougaard K, Christensen EF. Evaluation of pre-hospital trauma triage criteria: a prospective study at a Danish level I trauma centre. Acta Anaesthesiologica Scandinavica. 2007;51(9):1172-7.

288. Kjös HO, Lande TM, Eriksson E, Nordhaug D, Karevold A, Haaverstad R. Thoracic injuries at a regional trauma centre. Tidsskrift for den Norske Laegeforening. 2007;127(11):1496-9.

289. Krakau K, Hansson A, Karlsson T, de Boussard CN, Tengvar C, Borg J. Nutritional treatment of patients with severe traumatic brain injury during the first six months after injury. Nutrition. 2007;23(4):308-17.

290. Larsson Lund M, Fisher AG, Lexell J, Bernspång B. Impact on participation and autonomy questionnaire: internal scale validity of the Swedish version for use in people with spinal cord injury. Journal of Rehabilitation Medicine. 2007;39(2):156-62.

291. Liebach A, Nordenbo AM, Engberg AW. Subacute intensive rehabilitation after severe traumatic brain injury. Follow-up 6 months after discharge. [Danish]. Ugeskrift for Laeger. 2007;169(3):223-7.

292. Lund ML, Fisher AG, Lexell J, Bernspang B. Impact on participation and autonomy questionnaire: internal scale validity of the Swedish version for use in people with spinal cord injury. Journal of Rehabilitation Medicine. 2007;39(2):156-62.

293. Mainio A, Kyllonen T, Viilo K, Hakko H, Sarkioja T, Rasanen P. Traumatic brain injury, psychiatric disorders and suicide: A population-based study of suicide victims during the years 1988-2004 in Northern Finland. Brain Injury. 2007;21(8):851-5.

294. Mattila VA, Kuronen P, Pihlajamaeki H. Nature and risk factors of injury hospitalization in young adults: A follow-up of 135,987 military conscripts. Scandinavian Journal of Public Health. 2007;35(4):418-23.

295. Mattila VM, Parkkari J, Rimpela A. Adolescent survey non-response and later risk of death. A prospective cohort study of 78 609 persons with 11-year follow-up. BMC Public Health. 2007;7(87).

296. Myhre MC, Grogaard JB, Dyb GA, Sandvik L, Nordhov M. Traumatic head injury in infants and toddlers. Acta Paediatrica. 2007;96(8):1159-63.

297. Olivecrona M, Rodling-Wahlstrom M, Naredi S, Koskinen L-OD. Effective ICP reduction by decompressive craniectomy in patients with severe traumatic brain injury treated by an ICP-targeted therapy. Journal of Neurotrauma. 2007;24(6):927-35.

298. Osler M, Andersen A-MN, Laursen B, Lawlor DA. Cognitive function in childhood and early adulthood and injuries later in life: the Metropolit 1953 male birth cohort. International Journal of Epidemiology. 2007;36(1):212-9.

299. Polinder S, Meerding WJ, Mulder S, Petridou E, van Beeck E. Assessing the burden of injury in six European countries. Bulletin of the World Health Organization. 2007;85(1):27-34.

300. Puljula J, Savola O, Tuomivaara V, Pribula J, Hillbom M. Weekday distribution of head traumas in patients admitted to the emergency department of a city hospital: Effects of age, gender and drinking pattern. Alcohol and Alcoholism. 2007;42(5):474-9.

301. Ringdal KG, Lossius HM. Feasibility of comparing core data from existing trauma registries in Scandinavia. Reaching for a Scandinavian major trauma outcome study (MTOS). Scandinavian Journal of Surgery. 2007;96(4):325-31.

302. Simonsen LL, Sonne-Holm S, Krasheninnikoff M, Engberg AW. Symptomatic heterotopic ossification after very severe traumatic brain injury in 114 patients: Incidence and risk factors. Injury-International Journal of the Care of the Injured. 2007;38(10):1146-50.

303. Skaga NO, Eken T, Hestnes M, Jones JM, Steen PA. Scoring of anatomic injury after trauma: AIS 98 versus AIS 90-do the changes affect overall severity assessment? Injury. 2007;38(1):84-90.

304. Soberg HL, Bautz-Holter E, Roise O, Finset A. Long-term multidimensional functional consequences of severe multiple injuries two years after trauma: a prospective longitudinal cohort study. J Trauma. 2007;62(2):461-70.

305. Soberg HL, Finset A, Bautz-Holter E, Sandvik L, Roise O. Return to work after severe multiple injuries: a multidimensional approach on status 1 and 2 years postinjury. J Trauma. 2007;62(2):471-81.

306. Soreide K, Ellingsen CL, Knutson V. How dangerous is BASE jumping? An analysis of adverse events in 20,850 jumps from the Kjerag Massif, Norway. J Trauma. 2007;62(5):1113-7.

307. Soreide K, Krueger AJ, Vardal AL, Ellingsen CL, Soreide E, Lossius HM. Epidemiology and contemporary patterns of trauma deaths: Changing place, similar pace, older face. World Journal of Surgery. 2007;31(11):2092-103.

308. Soreide K, Petrone P, Asensio JA. Emergency thoracotomy in trauma: Rationale, risks, and realities. Scandinavian Journal of Surgery. 2007;96(1):4-10.

309. Soreide K, Soiland H, Lossius HM, Vetrhus M, Soreide JA, Soreide E. Resuscitative emergency thoracotomy in a Scandinavian trauma hospital--is it justified? Injury. 2007;38(1):34-42.

310. Sundstrom T, Sollid S, Wentzel-Larsen T, Wester K. Head injury mortality in the Nordic countries. Journal of Neurotrauma. 2007;24(1):147-53.

311. Uleberg O, Vinjevoll OP, Eriksson U, Aadahl P, Skogvoll E. Overtriage in trauma - what are the causes? Acta Anaesthesiologica Scandinavica. 2007;51(9):1178-83.

312. Ulvik A, Kvale R, Wentzel-Larsen T, Flaatten H. Multiple organ failure after trauma affects even long-term survival and functional status. Critical Care. 2007;11(5).

313. Ulvik A, Wentzel-Larsen T, Flaatten H. Trauma patients in the intensive care unit: short- and long-term survival and predictors of 30-day mortality. Acta Anaesthesiologica Scandinavica. 2007;51(2):171-7.

314. Waehrens EE, Fisher AG. Improving quality of ADL performance after rehabilitation among people with acquired brain injury. Scandinavian journal of occupational therapy. 2007;14(4):250-7.

315. Wiman E, Wikblad K, Idvall E. Trauma patients' encounters with the team in the emergency department-A qualitative study. International Journal of Nursing Studies. 2007;44(5):714-22.

316. Winqvist S, Jokelainen J, Luukinen H, Hillbom M. Parental alcohol misuse is a powerful predictor for the risk of traumatic brain injury in childhood. Brain Injury. 2007;21(10):1079-85.

317. Winqvist S, Lehtilahti M, Jokelainen J, Luukinen H, Hillbom M. Traumatic brain injuries in children and young adults: A birth cohort study from northern Finland. Neuroepidemiology. 2007;29(1-2):136-42.

318. Andelic N, Sigurdardottir S, Brunborg C, Roe C. Incidence of hospital-treated traumatic brain injury in the Oslo population. Neuroepidemiology. 2008;30(2):120-8.

319. Bach A, Bendix J, Hougaard K, Frischknecht Christensen E. Retroperitoneal packing as part of damage control surgery in a Danish trauma centre - fast, effective, and cost-effective. Scandinavian Journal of Trauma, Resuscitation and Emergency Medicine. 2008;16(4 Jul):1-4.

320. Backe SN, Andersson R. Monitoring the "tip of the iceberg": Ambulance records as a source of injury surveillance. Scandinavian Journal of Public Health. 2008;36(3):250-7.

321. Bellander BM, Sollid S, Kock-Jensen C, Juul N, Eskesen V, Sundström T, et al. Prehospital management of patients with severe head injuries. Scandinavian guidelines according to Brain Trauma Foundation. Läkartidningen. 2008;105(24-25):1834-8.

322. Elmqvist C, Fridlund B, Ekebergh M. More than medical treatment: the patient's first encounter with prehospital emergency care. International emergency nursing. 2008;16(3):185-92.

323. Gedeborg R, Engquist H, Berglund L, Michaelsson K. Identification of Incident Injuries in Hospital Discharge Registers. Epidemiology. 2008;19(6):860-7.

324. Hansen KS, Uggen PE, Brattebo G, Wisborg T. Team-oriented training for damage control surgery in rural trauma: A new paradigm. Journal of Trauma-Injury Infection and Critical Care. 2008;64(4):949-53.

325. Hansen TS, Larsen K, Engberg AW. The Association of Functional Oral Intake and Pneumonia in Patients With Severe Traumatic Brain Injury. Archives of Physical Medicine and Rehabilitation. 2008;89(11):2114-20.

326. Hasselberg M, Laflamme L. Road traffic injuries among young car drivers by country of origin and socioeconomic position. International Journal of Public Health. 2008;53(1):40-5.

327. Heskestad B, Baardsen R, Helseth E, Ingebrigtsen T. Guideline Compliance in Management of Minimal, Mild, and Moderate Head Injury: High Frequency of Noncompliance Among Individual Physicians Despite Strong Guideline Support From Clinical Leaders. Journal of Trauma-Injury Infection and Critical Care. 2008;65(6):1309-13.

328. Juul N, Sollid S, Sundstrom T, Kock-Jensen C, Eskesen V, Bellander B-M, et al. [Scandinavian guidelines on the pre-hospital management of traumatic brain injury]. Ugeskrift for laeger. 2008;170(26-32):2337-41.

329. Juul N, Sollid S, Sundström T, Kock-Jensen C, Eskesen V, Bellander BM, et al. Scandinavian guidelines on pre-hospital management of severe traumatic brain injury. Ugeskrift for Laeger. 2008;170(26-32):2337-41.

330. Juutilainen M, Vintturi J, Robinson S, Back L, Lehtonen H, Makitie AA. Laryngeal fractures: Clinical findings and considerations on suboptimal outcome. Acta Oto-Laryngologica. 2008;128(2):213-8.

331. Koskinen S, Alaranta H. Traumatic brain injury in Finland 1991-2005: A nationwide register study of hospitalized and fatal TBI. Brain Injury. 2008;22(3):205-14.

332. Lundberg L, Molde A, Dalenius E. BATLS/BARTS/BBTLS training for Swedish Armed Forces medical personnel--a ten year retrospective study. Journal of the Royal Army Medical Corps. 2008;154(1):34-7.

333. Moen KG, Klepstad P, Skandsen T, Fredriksli OA, Vik A. Direct transport versus interhospital transfer of patients with severe head injury in Norway. European Journal of Emergency Medicine. 2008;15(5):249-55.

334. Nordgren C. On the need of validating inpatient registers. Spinal Cord. 2008;46(11):748-52.

335. Polinder S, Meerding WJ, Lyons RA, Haagsma JA, Toet H, Petridou ET, et al. International variation in clinical injury incidence: Exploring the performance of indicators based on health care, anatomical and outcome criteria. Accident Analysis and Prevention. 2008;40(1):182-91.

336. Rekand T, Schaanning EE, Varga V, Schattel U, Gronning M. Spinal cord injuries among paragliders in Norway. Spinal Cord. 2008;46(6):412-6.

337. Rutberg L, Friden B, Karlsson AK. Amenorrhoea in newly spinal cord injured women: An effect of hyperprolactinaemia? Spinal Cord. 2008;46(3):189-91.

338. Shibuya H, Cleal B, Mikkelsen KL. Work injuries among drivers in the goods-transport branch in Denmark. American Journal of Industrial Medicine. 2008;51(5):364-71.

339. Shibuya H, Hannerz H, Mikkelsen KL, Cleal B, Gubba L. Hospital contacts due to injuries among male drivers working for road goods-transport contractors in Denmark. International Journal of Occupational Medicine and Environmental Health. 2008;21(1):59-66.

340. Skaga NO, Eken T, Jones JM, Steen PA. Different definitions of patient outcome: Consequences for performance analysis in trauma. Injury-International Journal of the Care of the Injured. 2008;39(5):612-22.

341. Soreide K, Soreide AH. Multiple lacerations in a pregnant woman caused by spontaneously exploding shower screen glass. American Journal of Emergency Medicine. 2008;26(9):1065.e1-2.

342. Ulvik A, Kvale R, Wentzel-Larsen T, Flaatten H. Quality of life 2-7 years after major trauma. Acta Anaesthesiologica Scandinavica. 2008;52(2):195-201.

343. Utsi R, Brandstorp H, Johansen K, Wisborg T. Training in multiprofessional emergency medicine in primary health care. [Norwegian]. Tidsskrift for den Norske laegeforening. 2008;128(9):1057-9.

344. Winqvist S, Luukinen H, Jokelainen J, Lehtilahti M, Nayha S, Hillbom M. Recurrent traumatic brain injury is predicted by the index injury occurring under the influence of alcohol. Brain Injury. 2008;22(10):780-5.

345. Wisborg T, Brattebo G. Keeping the spirit high: why trauma team training is (sometimes) implemented. Acta Anaesthesiologica Scandinavica. 2008;52(3):437-41.

346. Wisborg T, Brattebo G, Brinchmann-Hansen A, Uggen PE, Hansen KS. Effects of nationwide training of multiprofessional trauma teams in Norwegian hospitals. Journal of Trauma-Injury Infection and Critical Care. 2008;64(6):1613-8.

347. Woyen AV, Thomsen AB. [Is observation of trauma patients necessary if no significant injury is detected during primary evaluation?]. Ugeskrift for laeger. 2008;170(25):2225-7.

348. Ala-Kokko TI, Ohtonen P, Koskenkari J, Laurila JJ. Improved outcome after trauma care in university-level intensive care units. Acta Anaesthesiologica Scandinavica. 2009;53(10):1251-6.

349. Andelic N, Hammergren N, Bautz-Holter E, Sveen U, Brunborg C, Roe C. Functional outcome and health-related quality of life 10 years after moderate-to-severe traumatic brain injury. Acta Neurologica Scandinavica. 2009;120(1):16-23.

350. Biering-Sorensen T, Hansen RB, Biering-Sorensen F. Home aids and personal assistance 10-45 years after spinal cord injury. Spinal Cord. 2009;47(5):405-12.

351. Christensen J, Pedersen MG, Pedersen CB, Sidenius P, Olsen J, Vestergaard M. Long-term risk of epilepsy after traumatic brain injury in children and young adults: a population-based cohort study. Lancet. 2009;373(9669):1105-10.

352. Christensen P, Andreasen J, Ehlers L. Cost-effectiveness of transanal irrigation versus conservative bowel management for spinal cord injury patients. Spinal cord [Internet]. 2009; 47(2):[138-43 pp.]. Available from: http://onlinelibrary.wiley.com/o/cochrane/clcentral/articles/370/CN-00684370/frame.html

http://www.nature.com/sc/journal/v47/n2/pdf/sc200898a.pdf.

353. Franzen C, Bjornstig U, Brulin C, Lindholm L. A cost-utility analysis of nursing intervention via telephone follow-up for injured road users. Bmc Health Services Research. 2009;9.

354. Fromovich-Amit Y, Biering-Sorensen F, Baskov V, Juocevicius A, Hansen HV, Gelernter I, et al. Properties and outcomes of spinal rehabilitation units in four countries. Spinal Cord. 2009;47(8):597-603.

355. Gedeborg R, Thiblin I, Byberg L, Wernroth L, Michaelsson K. The impact of clinically undiagnosed injuries on survival estimates. Critical Care Medicine. 2009;37(2):449-55.

356. Hagen EM, Rekand T, Gilhus NE, Gronning M. Diagnostic coding accuracy for traumatic spinal cord injuries. Spinal Cord. 2009;47(5):367-71.

357. Heskestad B, Baardsen R, Helseth E, Romner B, Waterloo K, Ingebrigtsen T. Incidence of hospital referred head injuries in Norway: A population based survey from the Stavanger region. Scandinavian Journal of Trauma Resuscitation & Emergency Medicine. 2009;17.

358. Hjortdahl M, Ringen A, Naess AN, Wisborg T. Leadership is the essential human factor in the trauma team - Results of a qualitative study. Acta Anaesthesiologica Scandinavica, Supplement. 2009;53 (119):51-2.

359. Hjortdahl M, Ringen AH, Naess A-C, Wisborg T. Leadership is the essential non-technical skill in the trauma team - results of a qualitative study. Scandinavian Journal of Trauma Resuscitation & Emergency Medicine. 2009;17.

360. Holmqvist K, Kamwendo K, Ivarsson AB. Occupational therapists' descriptions of their work with persons suffering from cognitive impairment following acquired brain injury. Scandinavian Journal of Occupational Therapy. 2009;16(1):13-24.

361. Jacobsson LJ, Westerberg M, Soderberg S, Lexell J. Functioning and disability 6-15 years after traumatic brain injuries in northern Sweden. Acta Neurologica Scandinavica. 2009;120(6):389-95.

362. Johansson C, Bodin P, Kreuter M. Validity and responsiveness of the spinal cord index of function: An instrument on activity level. Spinal Cord. 2009;47(11):817-21.

363. Jumisko E, Lexell J, Soderberg S. The meaning of feeling well in people with moderate or severe traumatic brain injury. Journal of Clinical Nursing. 2009;18(16):2273-81.

364. Kosola S, Salminen P, Laine T. Heading for a Fall - Moped and Scooter Accidents from 2002 to 2007. Scandinavian Journal of Surgery. 2009;98(3):175-9.

365. Laursen B, Moller H. Unintentional injuries in children of Danish and foreign-born mothers. Scandinavian Journal of Public Health. 2009;37(6):577-83.

366. Meisler R, Berlac PA. [Prehospital trauma triage before and after deployment of a physician-manned mobile emergency care unit in Nordsjaelland]. Ugeskrift for laeger. 2009;171(36):2548-52.

367. Nakstad AR, Bjelland B, Sandberg M. Medical emergency motorcycle--is it useful in a Scandinavian Emergency Medical Service? Scandinavian journal of trauma, resuscitation and emergency medicine. 2009;17:9.

368. Norrman G, Tingstedt B, Ekelund M, Andersson R. Non-operative management of blunt liver trauma: Feasible and safe also in centres with a low trauma incidence. Hpb. 2009;11(1):50-6.

369. Olivecrona M, Zetterlund B, Rodling-Wahlstrom M, Naredi S, Koskinen L-OD. Absence of electroencephalographic seizure activity in patients treated for head injury with an intracranial pressure-targeted therapy. Journal of Neurosurgery. 2009;110(2):300-5.

370. Petersen JA, Christensen K, Steinmetz J. Penetrating trauma treated by the Medical Emergency Care Unit in Copenhagen. [Danish]. Ugeskrift for laeger. 2009;171(41):2982-5.

371. Ringdal M, Plos K, Lundberg D, Johansson L, Bergbom I. Outcome after injury: Memories, health-related quality of life, anxiety, and symptoms of depression after intensive care. Journal of Trauma - Injury, Infection and Critical Care. 2009;66(4):1226-33.

372. Rortveit S, Hunskar S. [Development of events in medical emergency situations in a rural community]. Tidsskrift for den Norske lageforening : tidsskrift for praktisk medicin, ny rakke. 2009;129(8):735-7.

373. Rortveit S, Hunskar S. [Medical emergencies in a rural community]. Tidsskrift for den Norske lageforening : tidsskrift for praktisk medicin, ny rakke. 2009;129(8):738-42.

374. Sigurdardottir S, Andelic N, Roe C, Jerstad T, Schanke A-K. Post-concussion symptoms after traumatic brain injury at 3 and 12 months post-injury: A prospective study. Brain Injury. 2009;23(6):489-97.

375. Sollid S, Sundstrom T, Ingebrigtsen T, Romner B, Wester K. Organisation of traumatic head injury management in the Nordic countries. Emergency Medicine Journal. 2009;26(11):769-72.

376. Stensballe J, Christiansen M, E TO, Espersen K, Lippert FK, Rasmussen LS. The early IL-6 and IL-10 response in trauma is correlated with injury severity and mortality. Acta Anaesthesiologica Scandinavica. 2009;53(4):515-21.

377. Suhonen R, Berg A, Idvall E, Kalafati M, Katajisto J, Land L, et al. European orthopaedic and trauma patients' perceptions of nursing care: a comparative study. Journal of Clinical Nursing. 2009;18(20):2818-29.

378. Veisten K, Nossum A, Akhtar J. Total costs of injury from accidents in the home and during education, sports and leisure activities: estimates for Norway with assessment of uncertainty. European Journal of Health Economics. 2009;10(3):337-46.

379. Wisborg T, Brattebo G, Brinchmann-Hansen A, Hansen KS. Mannequin or standardized patient: participants' assessment of two training modalities in trauma team simulation. Scandinavian Journal of Trauma Resuscitation & Emergency Medicine. 2009;17.

380. Wisborg T, Brattebø G, Brinchmann-Hansen A, Hansen KS. Mannequin or standardized patient: participants' assessment of two training modalities in trauma team simulation. Scandinavian journal of trauma, resuscitation and emergency medicine [Internet]. 2009; 17:[59 p.]. Available from: http://onlinelibrary.wiley.com/o/cochrane/clcentral/articles/587/CN-00769587/frame.html.

381. Zakariassen E, Holm Hansen E, Hunskaar S. Incidence of emergency contacts (red responses) to Norwegian emergency primary healthcare services in 2007 - a prospective observational study. Scandinavian Journal of Trauma, Resuscitation and Emergency Medicine. 2009;17(30):1-6.

382. Andelic N, Jerstad T, Sigurdardottir S, Schanke A-K, Sandvik L, Roe C. Effects of acute substance use and pre-injury substance abuse on traumatic brain injury severity in adults admitted to a trauma centre. Journal of trauma management & outcomes. 2010;4:6-.

383. Andersson EE, Lund J, Mansson J. Traumatic brain injury in children between 7-12 years of age. Developmental Neurorehabilitation. 2010;13(5):346-50.

384. Astrand R, Unden J, Hesselgard K, Reinstrup P, Romner B. Clinical Factors Associated with Intracranial Complications after Pediatric Traumatic Head Injury: An Observational Study of Children Submitted to a Neurosurgical Referral Unit. Pediatric Neurosurgery. 2010;46(2):101-9.

385. Berlin JM, Carlstrom ED. From artefact to effect: the organising effects of artefacts on teams. Journal of health organization and management. 2010;24(4):412-27.

386. Brattstrom O, Granath F, Rossi P, Oldner A. Early predictors of morbidity and mortality in trauma patients treated in the intensive care unit. Acta Anaesthesiologica Scandinavica. 2010;54(8):1007-17.

387. Corrigan JD, Selassie AW, Orman JA. The Epidemiology of Traumatic Brain Injury. Journal of Head Trauma Rehabilitation. 2010;25(2):72-80.

388. Dehli T, Bagenholm A, Johnsen LH, Osbakk SA, Fredriksen K, Bartnes K. [Seriously injured patients transferred from local hospitals to a university hospital]. Tidsskrift for Den Norske Laegeforening. 2010;130(15):1455-7.

389. Dehli T, Bågenholm A, Johnsen L-H, Osbakk SA, Fredriksen K, Bartnes K. Seriously injured patients transferred to a university hospital. Tidsskrift for den Norske Laegeforening. 2010;130(15):1455-7.

390. Dirks J, Jorgensen H, Jensen CH, Ostrowski SR, Johansson PI. Blood product ratio in acute traumatic coagulopathy--effect on mortality in a Scandinavian level 1 trauma centre. Scandinavian journal of trauma, resuscitation and emergency medicine. 2010;18:65.

391. Divanoglou A, Seiger A, Levi R. Acute management of traumatic spinal cord injury in a Greek and a Swedish region: a prospective, population-based study. Spinal Cord. 2010;48(6):477-82.

392. Divanoglou A, Westgren N, Bjelak S, Levi R. Medical conditions and outcomes at 1 year after acute traumatic spinal cord injury in a Greek and a Swedish region: A prospective, population-based study. Spinal Cord. 2010;48(6):470-6.

393. Divanoglou A, Westgren N, Seiger A, Hulting C, Levi R. Late Mortality During the First Year After Acute Traumatic Spinal Cord Injury: A Prospective, Population-Based Study. Journal of Spinal Cord Medicine. 2010;33(2):117-27.

394. Elmqvist C, Brunt D, Fridlund B, Ekebergh M. Being first on the scene of an accident--experiences of 'doing' prehospital emergency care. Scandinavian journal of caring sciences. 2010;24(2):266-73.

395. Gedeborg R, Thiblin I, Byberg L, Melhus H, Lindback J, Michaelsson K. Population density and mortality among individuals in motor vehicle crashes. Injury Prevention. 2010;16(5):302-8.

396. Hagen EM, Eide GE, Rekand T, Gilhus NE, Gronning M. Traumatic spinal cord injury and concomitant brain injury: a cohort study. Acta Neurologica Scandinavica. 2010;122:51-7.

397. Hermansen DT, Bilde A, Rasmussen N. Observation of tardive laryngeal edema after blunt trauma to the neck is not necessary: A 10-year retrospective analysis. European Archives of Oto-Rhino-Laryngology. 2010;267(1):95-100.

398. Heskestad B, Waterloo K, Baardsen R, Helseth E, Romner B, Ingebrigtsen T. No impact of early intervention on late outcome after minimal, mild and moderate head injury. Scandinavian journal of trauma, resuscitation and emergency medicine [Internet]. 2010; 18:[10 p.]. Available from: http://onlinelibrary.wiley.com/o/cochrane/clcentral/articles/684/CN-00742684/frame.html

http://www.ncbi.nlm.nih.gov/pmc/articles/PMC2844351/pdf/1757-7241-18-10.pdf.

399. Jacobsson LJ, Westerberg M, Lexell J. Health-related quality-of-life and life satisfaction 6-15 years after traumatic brain injuries in northern Sweden. Brain Injury. 2010;24(9):1075-86.

400. Janvier A, Hansen T, Davis P, Verhagen E, Laventhal N, Musante G, et al. Internatio nal comparison of triage for criticalyil patients: Children first, adults and neonates last. Paediatrics and Child Health. 2010;15:20A.

401. Kirves H, Handolin L, Niemela M, Pitkaniemi J, Randell T. Paramedics' and pre-hospital physicians' assessments of anatomic injury in trauma patients: a cohort study. Scandinavian Journal of Trauma Resuscitation & Emergency Medicine. 2010;18.

402. Kornfalt J, Johansson A. Occurrence of hypothermia in a prehospital setting, southern Sweden. International emergency nursing. 2010;18(2):76-9.

403. Kristiansen T, Soreide K, Ringdal KG, Rehn M, Kruger AJ, Reite A, et al. Trauma systems and early management of severe injuries in Scandinavia: Review of the current state. Injury-International Journal of the Care of the Injured. 2010;41(5):444-52.

404. Lannem AM, Sorensen M, Lidal IB, Hjeltnes N. Perceptions of exercise mastery in persons with complete and incomplete spinal cord injury. Spinal Cord. 2010;48(5):388-92.

405. Larsen KT, Uleberg O, Skogvoll E. Differences in trauma team activation criteria among Norwegian hospitals. Scandinavian Journal of Trauma Resuscitation & Emergency Medicine. 2010;18.

406. Lindfors N, Raatikainen T. Incidence, epidemiology, and operative outcome of replantation or revascularisation of injury to the upper extremity. Journal of Plastic Surgery and Hand Surgery. 2010;44(1):44-9.

407. Lindroos O, Burstrom L. Accident rates and types among self-employed private forest owners. Accident Analysis and Prevention. 2010;42(6):1729-35.

408. Linnarsson JR, Bubini J, Perseius K-I. Review: a meta-synthesis of qualitative research into needs and experiences of significant others to critically ill or injured patients. Journal of Clinical Nursing. 2010;19(21-22):3102-11.

409. Lundqvist A, Grundstrom K, Samuelsson K, Ronnberg J. Computerized training of working memory in a group of patients suffering from acquired brain injury. Brain Injury. 2010;24(10):1173-83.

410. Meerding WJ, Polinder S, Lyons RA, Petridou ET, Toet H, van Beeck EF, et al. How adequate are emergency department home and leisure injury surveillance systems for cross-country comparisons in Europe? International Journal of Injury Control and Safety Promotion. 2010;17(1):13-22.

411. Meisler R, Thomsen AB, Abildstrom H, Guldstad N, Borge P, Rasmussen SW, et al. Triage and mortality in 2875 consecutive trauma patients. Acta Anaesthesiologica Scandinavica. 2010;54(2):218-23.

412. Norup A, Siert L, Mortensen EL. Emotional distress and quality of life in relatives of patients with severe brain injury: The first month after injury. Brain Injury. 2010;24(2):81-8.

413. Pahle AS, Pedersen BL, Skaga NO, Pillgram-Larsen J. Emergency Thoracotomy Saves Lives in a Scandinavian Hospital Setting. Journal of Trauma-Injury Infection and Critical Care. 2010;68(3):599-603.

414. Pedersen BH, Hannerz H, Tuchsen F, Mikkelsen KL, Dyreborg J. Industry and injury related hospital contacts: a follow-up study of injuries among working men in Denmark. Journal of occupational health. 2010;52(3):147-54.

415. Perheentupa U, Kinnunen I, Grenman R, Aitasalo K, Makitie AA. Management and outcome of pediatric skull base fractures. International Journal of Pediatric Otorhinolaryngology. 2010;74(11):1245-50.

416. Polinder S, Haagsma JA, Toet H, Brugmans MJP, van Beeck EF. Burden of injury in childhood and adolescence in 8 European countries. Bmc Public Health. 2010;10.

417. Ringdal M, Plos K, Otenwall P, Bergbom I. Memories and health-related quality of life after intensive care: A follow-up study. Critical Care Medicine. 2010;38(1):38-44.

418. Scheiman S, Moghaddas HS, Bjornstig U, Bylund P-O, Saveman B-I. Bicycle injury events among older adults in Northern Sweden: A 10-year population based study. Accident Analysis and Prevention. 2010;42(2):758-63.

419. Sluys K, Lannge M, Iselius L, Eriksson LE. Outcomes in Pediatric Trauma Care in the Stockholm Region. European Journal of Trauma and Emergency Surgery. 2010;36(4):308-17.

420. Sollid SJ, Lossius HM, Nakstad AR, Aven T, Söreide E. Risk assessment of pre-hospital trauma airway management by anaesthesiologists using the predictive Bayesian approach. Scandinavian Journal of Trauma, Resuscitation and Emergency Medicine. 2010;18(22):1-10.

421. Sollid SJ, Lossius HM, Soreide E. Pre-hospital intubation by anaesthesiologists in patients with severe trauma: an audit of a Norwegian helicopter emergency medical service. Scandinavian journal of trauma, resuscitation and emergency medicine. 2010;18:30.

422. Soreide K. Temporal Patterns of Death after Trauma: Evaluation of Circadian, Diurnal, Periodical and Seasonal Trends in 260 Fatal Injuries. Scandinavian Journal of Surgery. 2010;99(4):235-9.

423. Suhonen R, Land L, Valimaki M, Berg A, Idvall E, Kalafati M, et al. Impact of patient characteristics on orthopaedic and trauma patients' perceptions of individualised nursing care. International journal of evidence-based healthcare. 2010;8(4):259-67.

424. Sun Y, Hsu P, Vestergaard M, Christensen J, Li J, Olsen J. Gestational Age, Birth Weight, and Risk for Injuries in Childhood. Epidemiology. 2010;21(5):650-7.

425. Toien K, Myhren H, Bredal IS, Skogstad L, Sandvik L, Ekeberg O. Psychological Distress After Severe Trauma: A Prospective 1-Year Follow-Up Study of a Trauma Intensive Care Unit Population. Journal of Trauma-Injury Infection and Critical Care. 2010;69(6):1552-9.

426. Whitley E, Batty GD, Gale CR, Deary IJ, Tynelius P, Rasmussen F. Intelligence in early adulthood and subsequent risk of unintentional injury over two decades: cohort study of 1 109 475 Swedish men. Journal of Epidemiology and Community Health. 2010;64(5):419-25.

427. Whitley E, Batty GD, Gale CR, Deary IJ, Tynelius P, Rasmussen F. Intelligence in Early Adulthood and Subsequent Risk of Assault: Cohort Study of 1,120,998 Swedish Men. Psychosomatic Medicine. 2010;72(4):390-6.

428. Wretstrand A, Bylund P-O, Petzall J, Falkmer T. Injuries in special transport services-Situations and risk levels involving wheelchair users. Medical Engineering & Physics. 2010;32(3):248-53.

429. Zakariassen E. Emergency cases to the EMC-centers in Norway - epidemiology, who is receives the alarm and who responds. Scandinavian Update Magazine. 2010;3(3):54-7.

430. Zakariassen E, Burman RA, Hunskaar S. The epidemiology of medical emergency contacts outside hospitals in Norway--a prospective population based study. Scandinavian journal of trauma, resuscitation and emergency medicine. 2010;18:9.

431. Bakke HK, Wisborg T. Rural High North: A High Rate of Fatal Injury and Prehospital Death. World Journal of Surgery. 2011;35(7):1615-20.

432. Bensch FV, Koivikko MP, Koskinen SK. MDCT findings in sports and recreational accidents. Acta radiologica (Stockholm, Sweden : 1987). 2011;52(10):1107-12.

433. Bergstrom MF, Byberg L, Melhus H, Michaelsson K, Gedeborg R. Extent and consequences of misclassified injury diagnoses in a national hospital discharge registry. Injury Prevention. 2011;17(2):108-13.

434. Bogstrand ST, Normann PT, Rossow I, Larsen M, Morland J, Ekeberg O. Prevalence of alcohol and other substances of abuse among injured patients in a Norwegian emergency department. Drug and Alcohol Dependence. 2011;117(2-3):132-8.

435. Christensen D, Jensen NM, Maaloe R, Rudolph SS, Belhage B, Perrild H. Low compliance with a validated system for emergency department triage. Danish Medical Bulletin. 2011;58(6).

436. Christensen D, Maaloe R, Jensen NM, Rudolph SS, Perrild H. Quality of care using a multidisciplinary team in the emergency room. Danish Medical Bulletin. 2011;58(6).

437. Dahlqvist P, Mattsson C, Olivecrona Z, Koskinen LOD. Pituitary function after severe traumatic brain injury (TBI) in northern sweden. Endocrine Reviews. 2011;32 (3 Meeting Abstracts).

438. Dehli T, Fredriksen K, Osbakk SA, Bartnes K. Evaluation of a university hospital trauma team activation protocol. Scandinavian Journal of Trauma Resuscitation & Emergency Medicine. 2011;19.

439. Ek AS. Personal experiences of sexuality after an acquired brain injury: An interview study. Journal of Sexual Medicine. 2011;8:162.

440. Fattah S, Ekas GR, Hyldmo PK, Wisborg T. The lateral trauma position: What do we know about it and how do we use it? A cross-sectional survey of all Norwegian emergency medical services. Scandinavian Journal of Trauma Resuscitation & Emergency Medicine. 2011;19.

441. Funder KS, Petersen JA, Steinmetz J. On-scene time and outcome after penetrating trauma: An observational study. Emergency Medicine Journal. 2011;28(9):797-801.

442. Godbolt AK, Tengvar C, Johansson B, Stenson S, Borg J. Opportunity for Inpatient Brain Injury Rehabilitation for Persons in a Vegetative State Survey of Swedish Physicians. American Journal of Physical Medicine & Rehabilitation. 2011;90(6):482-9.

443. Gouveia J, Seedat MA, Ekman R, Ekman DS, Bowman B. Tracing the utility of injury surveillance data in Pretoria (South Africa) and Boras (Sweden). International Journal of Injury Control and Safety Promotion. 2011;18(1):75-83.

444. Groven S, Eken T, Skaga NO, Roise O, Naess PA, Gaarder C. Long-Lasting Performance Improvement After Formalization of a Dedicated Trauma Service. Journal of Trauma-Injury Infection and Critical Care. 2011;70(3):569-74.

445. Groven S, Naess PA, Trondsen E, Gaarder C. A national survey on temporary and delayed abdominal closure in Norwegian hospitals. Scandinavian Journal of Trauma, Resuscitation and Emergency Medicine. 2011;19(51):1-4.

446. Hagen EM, Eide GE, Elgen I. Traumatic spinal cord injury among children and adolescents; a cohort study in western Norway. Spinal Cord. 2011;49(9):981-5.

447. Harr ME, Heskestad B, Ingebrigtsen T, Romner B, Ronning P, Helseth E. Alcohol consumption, blood alcohol concentration level and guideline compliance in hospital referred patients with minimal, mild and moderate head injuries. Scandinavian Journal of Trauma Resuscitation & Emergency Medicine. 2011;19.

448. Larsson Lund M, Lovgren-Engstrom AL, Lexell J. Using everyday technology to compensate for difficulties in task performance in daily life: Experiences in persons with acquired brain injury and their significant others. Disability and Rehabilitation: Assistive Technology. 2011;6(5):402-11.

449. Meisler R, Thomsen AB, Theilade P, Abildstrom H, Borge P, Treschow M, et al. Age-related differences in mechanism, cause, and location of trauma deaths. Minerva Anestesiologica. 2011;77(6):592-7.

450. Nakstad AR, Heimdal H-J, Strand T, Sandberg M. Incidence of desaturation during prehospital rapid sequence intubation in a physician-based helicopter emergency service. American Journal of Emergency Medicine. 2011;29(6):639-44.

451. Nakstad AR, Skaga NO, Pillgram-Larsen J, Gran B, Heier HE. Trends in transfusion of trauma victims - evaluation of changes in clinical practice. Scandinavian Journal of Trauma Resuscitation & Emergency Medicine. 2011;19.

452. Nakstad AR, Strand T, Sandberg M. Landing sites and intubation may influence helicopter emergency medical services on-scene time. Journal of Emergency Medicine. 2011;40(6):651-7.

453. Nilsson S, Levi R, Nordstrom A. Treatment-resistant sensory motor symptoms in persons with SCI may be signs of restless legs syndrome. Spinal Cord. 2011;49(6):754-6.

454. Norum J, Elsbak TM. Air ambulance services in the Arctic 1999-2009: A Norwegian study. International Journal of Emergency Medicine. 2011;4(1).

455. Overgaard M, Hoyer CB, Christensen EF. Long-Term Survival and Health-Related Quality of Life 6 to 9 Years After Trauma. Journal of Trauma-Injury Infection and Critical Care. 2011;71(2):435-41.

456. Ronning PA, Pedersen T, Skaga NO, Helseth E, Langmoen IA, Stavem K. External Validation of a Prognostic Model for Early Mortality After Traumatic Brain Injury. Journal of Trauma-Injury Infection and Critical Care. 2011;70(4):E56-E61.

457. Saveman B-I, Bjornstig U. Unintentional injuries among older adults in northern Sweden - a one-year population-based study. Scandinavian Journal of Caring Sciences. 2011;25(1):185-93.

458. Staff T, Sovik S. A retrospective quality assessment of pre-hospital emergency medical documentation in motor vehicle accidents in south-eastern Norway. Scandinavian Journal of Trauma Resuscitation & Emergency Medicine. 2011;19.

459. Strandroth J, Rizzi M, Sternlund S, Lie A, Tingvall C. The Correlation Between Pedestrian Injury Severity in Real-Life Crashes and Euro NCAP Pedestrian Test Results. Traffic Injury Prevention. 2011;12(6):604-13.

460. Suominen JS, Pakarinen MP, Kaariainen S, Impinen A, Vartiainen E, Helenius I. In-Hospital Treated Pediatric Injuries Are Increasing in Finland - a Population Based Study between 1997 and 2006. Scandinavian Journal of Surgery. 2011;100(2):129-35.

461. Taylor JS, Ruiz MA, Soler MD, Bouhassira D, Poole H, Jauregui ML, et al. Initial psychometric properties of the eurodolmed questionnaire: A new instrument to measure neuropathic pain in patients with spinal cord injury (SCI) based on pain intensity, pain interference and pain descriptors. Value in Health. 2011;14 (7):A326.

462. Toien K, Bredal IS, Skogstad L, Myhren H, Ekeberg O. Health related quality of life in trauma patients. Data from a one-year follow up study compared with the general population. Scandinavian Journal of Trauma Resuscitation & Emergency Medicine. 2011;19.

463. van Langeveld SA, Post MW, van Asbeck FW, Gregory M, Halvorsen A, Rijken H, et al. Comparing content of therapy for people with a spinal cord injury in postacute inpatient rehabilitation in Australia, Norway, and The Netherlands. Physical therapy. 2011;91(2):210-24.

464. Vassend O, Quale AJ, Roise O, Schanke AK. Predicting the long-term impact of acquired severe injuries on functional health status: The role of optimism, emotional distress and pain. Spinal Cord. 2011;49(12):1193-7.

465. Abelsson A, Lindwall L. The Prehospital assessment of severe trauma patients' performed by the specialist ambulance nurse in Sweden - a phenomenographic study. Scandinavian Journal of Trauma Resuscitation & Emergency Medicine. 2012;20.

466. al-Ayoubi F, Eriksson H, Myrelid P, Wallon C, Andersson P. Distribution of emergency operations and trauma in a Swedish hospital: need for reorganisation of acute surgical care? Scandinavian Journal of Trauma Resuscitation & Emergency Medicine. 2012;20.

467. Andelic N, Anke A, Skandsen T, Sigurdardottir S, Sandhaug M, Ader T, et al. Incidence of Hospital-Admitted Severe Traumatic Brain Injury and In-Hospital Fatality in Norway: A National Cohort Study. Neuroepidemiology. 2012;38(4):259-67.

468. Andelic N, Bautz-Holter E, Ronning P, Olafsen K, Sigurdardottir S, Schanke AK, et al. Does an early onset and continuous chain of rehabilitation improve the long-term functional outcome of patients with severe traumatic brain injury? Journal of Neurotrauma. 2012;29(1):66-74.

469. Andelic N, Roe C. Is an early onset and continuous chain of rehabilitation beneficial for severe traumatic brain injury in five years perspective? Journal of Neurotrauma. 2012;29 (10):A37.

470. Andelic N, Stevens LF, Sigurdardottir S, Arango-Lasprilla JC, Roe C. Associations between disability and employment 1 year after traumatic brain injury in a working age population. Brain Injury. 2012;26(3):261-9.

471. Andelic N, Sveen U, Anke A, Skandsen T, Sigurdardottir S, Berntsen S, et al. Association between early-introduced rehabilitation and short-term functional outcome in severe traumatic brain injury. Brain Injury. 2012;26 (4-5):542-3.

472. Andersen M. Criteria based emergency medical dispatch - A novelty in Denmark. Resuscitation. 2012;83:e18.

473. Aulanko M, Handolin L, Soderlund T, Pajarinen J. Accidental Falls Related to Shovelling Snow from Rooftops: Analysis of Injuries in an Extraordinary Epidemic in Southern Finland. Scandinavian Journal of Surgery. 2012;101(4):271-4.

474. Bogstrand ST, Gjerde H, Normann PT, Rossow I, Ekeberg O. Alcohol, psychoactive substances and non-fatal road traffic accidents--a case-control study. BMC public health. 2012;12:734.

475. Brattstrom O, Larsson E, Granath F, Riddez L, Bell M, Oldner A. Time dependent influence of host factors on outcome after trauma. European Journal of Epidemiology. 2012;27(3):233-41.

476. Brink O, Borris LC, Hougaard K. Effective treatment at a Danish trauma centre. Danish Medical Journal. 2012;59(3).

477. Cederberg D, Figaji A, Siesjo P. Cytokine analysis in paediatric severe traumatic brain injury. Brain Injury. 2012;26 (4-5):719-20.

478. Do HQ, Steinmetz J, Rasmussen LS. In-hospital mortality pattern of severely injured children. Injury. 2012;43(12):2060-4.

479. Eide LS, Schanke AK. Examining computerized working memory training as a supplement to cognitive rehabilitation for patients with acquired brain injury. Brain Injury. 2012;26 (4-5):749-50.

480. Falk A-C, Lindström V, Bergvall T, Castren M. Guidelines for prehospital care in severe head injuries are not followed. Big difference between the Scandinavian guidelines and the regional Swedish guidelines. Läkartidningen. 2012;109(17-18):882-3.

481. Gedeborg R, Chen LH, Thiblin I, Byberg L, Melhus H, Michaelsson K, et al. Prehospital injury deaths--strengthening the case for prevention: nationwide cohort study. J Trauma Acute Care Surg. 2012;72(3):765-72.

482. Hedstrom EM, Bergstrom U, Michno P. Injuries in children and adolescents--analysis of 41,330 injury related visits to an emergency department in northern Sweden. Injury. 2012;43(9):1403-8.

483. Hejselbaek J, Steinmetz J, Rasmussen LS. Prehospital guidelines for use of hypertonic saline are not followed systematically. Danish Medical Journal. 2012;59(4).

484. Hesselfeldt R, Steinmetz J, Rasmussen LS. A physician staffed helicopter improves triage and reduces mortality for severely injured trauma patients. European Journal of Anaesthesiology. 2012;29:2.

485. Hien Quoc D, Steinmetz J, Rasmussen LS. In-hospital mortality pattern of severely injured children. Injury-International Journal of the Care of the Injured. 2012;43(12):2060-4.

486. Joergensen HS. The need of hospital and community health care services for the rehabilitation of acquired brain injury and stroke. Neurorehabilitation and Neural Repair. 2012;26 (6):729.

487. Johansson J, Blomberg H, Svennblad B, Wernroth L, Melhus H, Byberg L, et al. Prehospital Trauma Life Support (PHTLS) training of ambulance caregivers and impact on survival of trauma victims. Resuscitation. 2012;83(10):1259-64.

488. Johansson L, Stenlund H, Bylund P-O, Eriksson A. ER visits predict premature death among teenagers. Accident Analysis and Prevention. 2012;48:397-400.

489. Johansson PI, Sorensen AM, Perner A, Welling KL, Wanscher M, Larsen CF, et al. High sCD40L levels early after trauma are associated with enhanced shock, sympathoadrenal activation, tissue and endothelial damage, coagulopathy and mortality. Journal of Thrombosis and Haemostasis. 2012;10(2):207-16.

490. Johansson PI, Stensballe J, Rasmussen LS, Ostrowski SR. High circulating adrenaline levels at admission predict increased mortality after trauma. J Trauma Acute Care Surg. 2012;72(2):428-36.

491. Jorgensen HS. A national strategy for the rehabilitation of patients with acquired brain injury and stroke in Denmark. Neurorehabilitation and Neural Repair. 2012;26 (6):729-30.

492. Kristiansen T, Rehn M, Gravseth HM, Lossius HM, Kristensen P. Paediatric trauma mortality in Norway: A population-based study of injury characteristics and urban-rural differences. Injury-International Journal of the Care of the Injured. 2012;43(11):1865-72.

493. Kristiansen T, Ringdal KG, Skotheimsvik T, Salthammer HK, Gaarder C, Naess PA, et al. Implementation of recommended trauma system criteria in south-eastern Norway: a cross-sectional hospital survey. Scandinavian Journal of Trauma Resuscitation & Emergency Medicine. 2012;20.

494. Lafrenz T, Lindberg SO, La Cour JL, Folkestad L, Hallas P, Brabrand M. Emergency teams in Danish emergency departments. Danish Medical Journal. 2012;59(6).

495. Laursen B, Helweg-Larsen K. Health service use in adults 20-64 years with traumatic brain injury, spinal cord injury or pelvic fracture. A cohort study with 9-year follow-up. Bmj Open. 2012;2(5).

496. Lindqvist K. Motala Municipality - a sustainable Safe Community in Sweden. International Journal of Injury Control and Safety Promotion. 2012;19(3):249-59.

497. Luukkainen S, Riala K, Laukkanen M, Hakko H, Rasanen P. Association of traumatic brain injury with criminality in adolescent psychiatric inpatients from Northern Finland. Psychiatry Research. 2012;200(2-3):767-72.

498. Norda R, Andersson TML, Edgren G, Nyren O, Reilly M. The impact of plasma preparations and their storage time on short-term posttransfusion mortality: A population-based study using the Scandinavian Donation and Transfusion database. Journal of Trauma and Acute Care Surgery. 2012;72(4):954-60.

499. Olofsson E, Bunketorp O, Andersson A-L. Children at risk of residual physical problems after public road traffic injuries-A 1-year follow-up study. Injury-International Journal of the Care of the Injured. 2012;43(1):84-90.

500. Orwelius L, Bergkvist M, Nordlund A, Simonsson E, Nordlund P, Backman C, et al. Physical effects of trauma and the psychological consequences of preexisting diseases account for a significant portion of the health-related quality of life patterns of former trauma patients. Journal of Trauma and Acute Care Surgery. 2012;72(2):504-12.

501. Oyo-Ita A, Ugare Udey G, Ikpeme Ikpeme A. Surgical versus non-surgical management of abdominal injury. Cochrane Database of Systematic Reviews [Internet]. 2012; (11). Available from: http://onlinelibrary.wiley.com/doi/10.1002/14651858.CD007383.pub2/abstract.

502. Perheentupa U, Kinnunen I, Grenman R, Aitasalo K, Karhu JO, Makitie AA. Post-traumatic morbidity is frequent in children with frontobasilar fractures. International Journal of Pediatric Otorhinolaryngology. 2012;76(5):670-4.

503. Renstrom B, Soderman K, Domellof E, Emanuelson I. Self-reported health and influence on life situation 5-8 years after paediatric traumatic brain injury. Brain Injury. 2012;26(12):1405-14.

504. Roe C, Anke A, Skandsen T, Manskow U, Andelic N. The effect of age on injury mechanisms, treatment and early outcome in patients with severe traumatic brain injury in norway. Brain Injury. 2012;26 (4-5):436.

505. Soreide JA, Konradsson A, Sandvik OM, Ovrebo K, Viste A. Esophageal perforation: Clinical patterns and outcomes from a patient cohort of western Norway. Digestive Surgery. 2012;29(6):494-502.

506. Staff T, Eken T, Hansen TB, Steen PA, Sovik S. A field evaluation of real-life motor vehicle accidents: Presence of unrestrained objects and their association with distribution and severity of patient injuries. Accident Analysis and Prevention. 2012;45:529-38.

507. Tenovuo O, Menon DK, Katila A, Coles J, Frantzen J, Outtrim J. The international TBIcare study - Preliminary results. Journal of Neurotrauma. 2012;29 (10):A187.

508. Toien K, Skogstad L, Ekeberg O, Myhren H, Bredal IS. Prevalence and predictors of return to work in hospitalised trauma patients during the first year after discharge: A prospective cohort study. Injury-International Journal of the Care of the Injured. 2012;43(9):1606-13.

509. Tuominen R, Joelsson P, Tenovuo O. Treatment costs and productivity losses caused by traumatic brain injuries. Brain Injury. 2012;26(13-14):1697-701.

510. Ulfarsson T, Gudnason G, Rosen T, Lundgren-Nilsson A, Nilsson M. Hypopituitarism in adults in the long-term perspective after severe traumatic brain injury: Relationship to outcome. Brain Injury. 2012;26 (4-5):691-2.

511. Vaaramo K, Puljula J, Tetri S, Juvela S, Hillbom M. Mortality of of Harmful Drinkers Increased after Reduction of Alcohol Prices in Northern Finland: A 10-Year Follow-Up of Head Trauma Subjects. Neuroepidemiology. 2012;39(3-4):156-62.

512. Zetterlund P, Plos K, Bergbom I, Ringdal M. Memories from intensive care unit persist for several years-A longitudinal prospective multi-centre study. Intensive and Critical Care Nursing. 2012;28(3):159-67.

513. Afzali M, Hesselfeldt R, Steinmetz J, Thomsen AB, Rasmussen LS. A helicopter emergency medical service may allow faster access to highly specialised care. Danish Medical Journal. 2013;60(7).

514. Andersen MS, Johnsen SP, Sörensen JN, Jepsen SB, Hansen JB, Christensen EF. Implementing a nationwide criteria-based emergency medical dispatch system: a register-based follow-up study. Scandinavian Journal of Trauma, Resuscitation and Emergency Medicine. 2013;21(53):1-8.

515. Bakkelund KE, Sundland E, Moen S, Vangberg G, Mellesmo S, Klepstad P. Undertreatment of pain in the prehospital setting: a comparison between trauma patients and patients with chest pain. European Journal of Emergency Medicine. 2013;20(6):428-30.

516. Blomberg H, Svennblad B, Michaelsson K, Byberg L, Johansson J, Gedeborg R. Prehospital Trauma Life Support Training of Ambulance Caregivers and the Outcomes of Traffic-Injury Victims in Sweden. Journal of the American College of Surgeons. 2013;217(6):1010-+.

517. Drange OK, Vaaler AE, Morken G, Finseth PI. Clinical course of disease and epileptic seizures in patients with bipolar disorder and a history of traumatic brain injury. European Neuropsychopharmacology. 2013;23:S331.

518. Fjaeldstad A, Kirk MH, Knudsen L, Bjerring J, Christensen EF. Physician-staffed emergency helicopter reduces transportation time from alarm call to highly specialized centre. Danish Medical Journal. 2013;60(7).

519. Groven S, Naess PA, Skaga NO, Gaarder C. Effects of moving emergency trauma laparotomies from the ED to a dedicated OR. Scandinavian Journal of Trauma Resuscitation & Emergency Medicine. 2013;21.

520. Hagemo JS, Naess PA, Johansson P, Windelov NA, Cohen MJ, Roislien J, et al. Evaluation of TEG (R) and RoTEM (R) inter-changeability in trauma patients. Injury-International Journal of the Care of the Injured. 2013;44(5):600-5.

521. Hagemo JS, Naess PA, Johansson P, Windelov NA, Cohen MJ, Roislien J, et al. Evaluation of TEG() and RoTEM() inter-changeability in trauma patients. Injury. 2013;44(5):600-5.

522. Halldorsson JG, Arnkelsson GB, Tomasson K, Flekkoy KM, Magnadottir HB, Arnarson EO. Long-term outcome of medically confirmed and self-reported early traumatic brain injury in two nationwide samples. Brain Injury. 2013;27(10):1106-18.

523. Halmin M, Bostrom F, Brattstrom O, Lundahl J, Wikman A, Ostlund A, et al. Effect of plasma-to-RBC ratios in trauma patients: a cohort study with time-dependent data. Critical Care Medicine. 2013;41(8):1905-14.

524. Hesselfeldt R, Steinmetz J, Jans H, Jacobsson ML, Andersen DL, Buggeskov K, et al. Impact of a physician-staffed helicopter on a regional trauma system: a prospective, controlled, observational study. Acta Anaesthesiologica Scandinavica. 2013;57(5):660-8.

525. Johannesdottir BK, Mogensen B, Gudbjartsson T. Emergency thoracotomy as a rescue treatment for trauma patients in Iceland. Injury. 2013;44(9):1186-90.

526. Jonsson K, Fridlund B. A comparison of adherence to correctly documented triage level of critically ill patients between emergency department and the ambulance service nurses. International Emergency Nursing. 2013;21(3):204-9.

527. Kammersgaard LP, Linnemann M, Tibaek M. Hydrocephalus following severe traumatic brain injury in adults. Incidence, timing, and clinical predictors during rehabilitation. Neurorehabilitation. 2013;33(3):473-80.

528. Karlsen AM, Thomassen O, Vikenes BH, Brattebo G. Equipment to prevent, diagnose, and treat hypothermia: a survey of Norwegian pre-hospital services. Scandinavian journal of trauma, resuscitation and emergency medicine. 2013;21:63.

529. Korhonen N, Kannus P, Niemi S, Palvanen M, Parkkari J. Fall-induced deaths among older adults: nationwide statistics in Finland between 1971 and 2009 and prediction for the future. Injury. 2013;44(6):867-71.

530. Kruger AJ, Lossius HM, Mikkelsen S, Kurola J, Castren M, Skogvoll E. Pre-hospital critical care by anaesthesiologist-staffed pre-hospital services in Scandinavia: a prospective population-based study. Acta Anaesthesiologica Scandinavica. 2013;57(9):1175-85.

531. Languren G, Montiel T, Julio-Amilpas A, Massieu L. Neuronal damage and cognitive impairment associated with hypoglycemia: An integrated view. Neurochemistry International. 2013;63(4):331-43.

532. Lossius HM, Kruger AJ, Ringdal KG, Sollid SJM, Lockey DJ. Developing templates for uniform data documentation and reporting in critical care using a modified nominal group technique. Scandinavian Journal of Trauma Resuscitation & Emergency Medicine. 2013;21.

533. Luoto TM, Artsola M, Helminen M, Liimatainen S, Kosunen E, Ohman J. [Acute head injuries in primary health care--internet survey conducted with general practitioners]. Duodecim; laaketieteellinen aikakauskirja. 2013;129(9):966-71.

534. Nordstrom C-H, Nielsen TH, Jacobsen A. Techniques and Strategies in Neurocritical Care Originating from Southern Scandinavia. Journal of Rehabilitation Medicine. 2013;45(8):710-7.

535. Nyholm L, Howells T, Enblad P, Lewen A. Introduction of the Uppsala Traumatic Brain Injury register for regular surveillance of patient characteristics and neurointensive care management including secondary insult quantification and clinical outcome. Upsala Journal of Medical Sciences. 2013;118(3):169-80.

536. Puljula J, Makinen E, Cygnel H, Kortelainen ML, Hillbom M. Incidence of moderate-to-severe traumatic brain injuries after reduction in alcohol prices. Acta Neurologica Scandinavica. 2013;127(3):192-7.

537. Raj R, Bendel S, Reinikainen M, Kivisaari R, Siironen J, Lang M, et al. Hyperoxemia and long-term outcome after traumatic brain injury. Critical Care. 2013;17(4).

538. Raj R, Siironen J, Kivisaari R, Kuisma M, Brinck T, Lappalainen J, et al. Factors correlating with delayed trauma center admission following traumatic brain injury. Scandinavian Journal of Trauma Resuscitation & Emergency Medicine. 2013;21.

539. Raninen AL, Olerud C, Robinson Y. Increased mortality of conservatively treated C2 fractures in the elderly population. European Spine Journal. 2013;1):S657.

540. Ringdal KG, Skaga NO, Hestnes M, Steen PA, Roislien J, Rehn M, et al. Abbreviated Injury Scale: not a reliable basis for summation of injury severity in trauma facilities? Injury. 2013;44(5):691-9.

541. Roe C, Skandsen T, Anke A, Ader T, Vik A, Lund SB, et al. Severe traumatic brain injury in Norway: impact of age on outcome. Journal of rehabilitation medicine. 2013;45(8):734-40.

542. Rognås L, Hansen TM, Kirkegaard H, Tönnesen E. Standard operating procedure changed pre-hospital critical care anaesthesiologists' behaviour: a quality control study. Scandinavian Journal of Trauma, Resuscitation and Emergency Medicine. 2013;21(84):1-7.

543. Röe C, Skandsen T, Anke A, Ader T, Vik A, Lund SB, et al. Severe traumatic brain injury in Norway: impact of age on outcome. Journal of Rehabilitation Medicine. 2013;45(8):734-40.

544. Raatiniemi L, Lankimaki S, Martikainen M. Pre-hospital airway management by non-physicians in Northern Finland - a cross-sectional survey. Acta Anaesthesiologica Scandinavica. 2013;57(5):654-9.

545. Raatiniemi L, Mikkelsen K, Fredriksen K, Wisborg T. Do pre-hospital anaesthesiologists reliably predict mortality using the NACA severity score? A retrospective cohort study. Acta Anaesthesiologica Scandinavica. 2013;57(10):1253-9.

546. Raatiniemi L, Mikkelsen K, Fredriksen K, Wisborg T. Does prehospital NACA score predict mortality and need for advanced treatment in hospital? Acta Anaesthesiologica Scandinavica, Supplement. 2013;57:21.

547. Siponkoski S-T, Wilson L, von Steinbuechel N, Sarajuuri J, Koskinen S. Quality of Life after Traumatic Brain Injury: Finnish Experience of the Qolibri in Residential Rehabilitation. Journal of Rehabilitation Medicine. 2013;45(8):835-42.

548. Skoglund K, Enblad P, Marklund N. Monitoring and Sedation Differences in the Management of Severe Head Injury and Subarachnoid Hemorrhage Among Neurocritical Care Centers. Journal of Neuroscience Nursing. 2013;45(6):360-8.

549. Stenberg M, Koskinen L-O, Levi R, Stalnacke B-M. Severe Traumatic Brain Injuries in Northern Sweden: A Prospective 2-Year Study. Journal of Rehabilitation Medicine. 2013;45(8):792-800.

550. Thelin EP, Johannesson L, Nelson D, Bellander B-M. S100B Is an Important Outcome Predictor in Traumatic Brain Injury. Journal of Neurotrauma. 2013;30(7):519-28.

551. Thordardottir EB, Haraldsdottir S. Prevalence and characteristics of accidents in the Icelandic population 2012. European Journal of Epidemiology. 2013;1):S250.

552. Virk J, Hsu P, Olsen J. Socio-demographic characteristics of women sustaining injuries during pregnancy: A study from the Danish national birth cohort. European Journal of Epidemiology. 2013;1):S61.

553. Zwisler S, Hallas J, Larsen MS, Handberg G, Mikkelsen S, Enggaard T. Legal opioid use before and after high energy trauma. Acta Anaesthesiologica Scandinavica, Supplement. 2013;57:4.

554. Andelic N, Soberg HL, Berntsen S, Sigurdardottir S, Roe C. Self-Perceived Health Care Needs and Delivery of Health Care Services 5 Years After Moderate-to-Severe Traumatic Brain Injury. Pm&R. 2014;6(11):1013-21.

555. Andelic N, Ye J, Tornas S, Roe C, Lu J, Bautz-Holter E, et al. Cost-effectiveness analysis (CEA) of continuous chain of rehabilitation after severe traumatic brain injury. Brain Injury. 2014;28 (5-6):609-10.

556. Bakke HK, Dehli T, Wisborg T. Fatal injury caused by low-energy trauma - a 10-year rural cohort. Acta Anaesthesiologica Scandinavica. 2014;58(6):726-32.

557. Bauer R, Steiner M, Kisser R, Macey SM, Thayer D. Accidents and injuries in the EU. Results of the EuroSafe Reports. Bundesgesundheitsblatt-Gesundheitsforschung-Gesundheitsschutz. 2014;57(6):673-80.

558. Becker F, Kirmess M, Tornas S, Lovstad M. A description of cognitive rehabilitation at Sunnaas Rehabilitation Hospital - Balancing comprehensive holistic rehabilitation and retraining of specific functional domains. Neurorehabilitation. 2014;34(1):87-100.

559. Bendixen AB, Hansen IS, Morild I, Lilleng PK. Deaths from violent causes and accidents in Hordaland County 2003-04. Tidsskrift for den Norske lageforening : tidsskrift for praktisk medicin, ny rakke. 2014;134(1):27-30.

560. Brorsson C, Dahlqvist P, Nilsson L, Thunberg J, Sylvan A, Naredi S. Adrenal response after trauma is affected by time after trauma and sedative/analgesic drugs. Injury. 2014;45(8):1149-55.

561. Ellensen EN, Hunskaar S, Wisborg T, Zakariassen E. Variations in contact patterns and dispatch guideline adherence between Norwegian emergency medical communication centres--a cross-sectional study. Scand J Trauma Resusc Emerg Med. 2014;22:2.

562. Eriksson M, Brattstrom O, Martensson J, Larsson E, Oldner A. Risk factors for trauma-induced acute kidney injury. Intensive Care Medicine. 2014;1):S93.

563. Falk A-C, Alm A, Lindstrom V. Has increased nursing competence in the ambulance services impacted on pre-hospital assessment and interventions in severe traumatic brain-injured patients? Scandinavian Journal of Trauma Resuscitation & Emergency Medicine. 2014;22.

564. Fattah S, Johnsen AS, Andersen JE, Vigerust T, Olsen T, Rehn M. Rapid extrication of entrapped victims in motor vehicle wreckage using a Norwegian chain method - cross-sectional and feasibility study. BMC Emergency Medicine. 2014;14(1).

565. Fazel S, Wolf A, Pillas D, Lichtenstein P, Langstrom N. Suicide, fatal injuries, and other causes of premature mortality in patients with traumatic brain injury a 41-year swedish population study. JAMA Psychiatry. 2014;71(3):326-33.

566. Fischerstrom A, Nyholm L, Lewen A, Enblad P. Acute neurosurgery for traumatic brain injury by general surgeons in Swedish county hospitals: A regional study. Acta Neurochirurgica. 2014;156(1):177-85.

567. Forslund MV, Arango-Lasprilla JC, Roe C, Perrin PB, Andelic N. Multilevel modeling of partnered relationship trajectories and relationship stability at 1, 2, and 5 years after traumatic brain injury in Norway. Neurorehabilitation. 2014;34(4):781-8.

568. Forslund MV, Arango-Lasprilla JC, Roe C, Perrin PB, Sigurdardottir S, Andelic N. Multi-level modelling of employment probability trajectories and employment stability at 1, 2 and 5 years after traumatic brain injury. Brain Injury. 2014;28(7):980-6.

569. Frantzen J, Katila A, Takala R, Ala-Seppala H, Kyllonen A, Maanpaa HR, et al. Injury profiles, demographic features and outcomes in 200 unselected consecutive adults with acute TBI attending a regional emergency care unit. Brain Injury. 2014;28 (5-6):777.

570. Gedeborg R, Warner M, Chen L-H, Gulliver P, Cryer C, Robitaille Y, et al. Internationally comparable diagnosis-specific survival probabilities for calculation of the ICD-10-based Injury Severity Score. Journal of Trauma and Acute Care Surgery. 2014;76(2):358-65.

571. Ghorbani P, Falken M, Riddez L, Sundelöf M, Oldner A, Strömmer L. Clinical review is essential to evaluate 30-day mortality after trauma. Scandinavian Journal of Trauma, Resuscitation and Emergency Medicine. 2014;22(18):1-7.

572. Gisladottir EH, Karason S, Sigvaldason K, Ulfarsson E, Mogensen B. [Visits to an emergency department due to head injuries]. Laeknabladid. 2014;100(6):331-5.

573. Halldorsson J, Arnkelsson G, Tomasson K, Flekkoy K, Magnadottir H, Arnarson E. Incidence, prevalence and prognostic factors-Findings from the ICTBI research project. Brain Injury. 2014;28 (5-6):550.

574. Halldorsson J, Arnkelsson G, Tomasson K, Flekkoy K, Magnadottir H, Arnarson E. TBI in infancy and early childhood-Findings from the ICTBI research project. Brain Injury. 2014;28 (5-6):550.

575. Hart T, Kozlowski AJ, Whyte J, Poulsen I, Kristensen K, Nordenbo A, et al. Functional Recovery After Severe Traumatic Brain Injury: An Individual Growth Curve Approach. Archives of Physical Medicine and Rehabilitation. 2014;95(11):2103-10.

576. Jelenkovic A, Silventoinen K, Tynelius P, Rasmussen F. Association of paternal IQ in early adulthood with offspring mortality and hospital admissions for injuries: a cohort study of 503 492 Swedish children. Journal of Epidemiology and Community Health. 2014;68(7):679-82.

577. Jensen EK, Biering-Sorensen F. Medication before and after a spinal cord lesion. Spinal Cord. 2014;52(5):358-63.

578. Jones JM, Skaga NO, Sovik S, Lossius HM, Eken T. Norwegian survival prediction model in trauma: modelling effects of anatomic injury, acute physiology, age, and co-morbidity. Acta Anaesthesiologica Scandinavica. 2014;58(3):303-15.

579. Kalzen H, Larsson BA, Eksborg S, Lindberg L, Edberg CE, Frostell C. Long term mortality in children admitted to pediatric intensive care in Sweden 2008-2010. Intensive Care Medicine. 2014;1):S248.

580. Kemppainen S, Pitkanen A, Tenovuo O, Sillanpaa M. The incidence of traumatic brain injury in the County of Kainuu, Finland: The Kainuu TBI cohort. Journal of Neurotrauma. 2014;31 (12):A44.

581. Klose M, Stochholm K, Janukonyte J, Christensen LL, Frystyk J, Andersen M, et al. Prevalence of Posttraumatic Growth Hormone Deficiency Is Highly Dependent on the Diagnostic Set-up: Results From The Danish National Study on Posttraumatic Hypopituitarism. Journal of Clinical Endocrinology & Metabolism. 2014;99(1):101-10.

582. Koskinen EA, Hakulinen U, Brander AE, Luoto TM, Ylinen A, Ohman JE. Clinical correlates of cerebral diffusion tensor imaging findings in chronic traumatic spinal cord injury. Spinal Cord. 2014;52(3):202-8.

583. Kristiansen T, Lossius HM, Rehn M, Kristensen P, Gravseth HM, Roislien J, et al. Epidemiology of trauma: A population-based study of geographical risk factors for injury deaths in the working-age population of Norway. Injury-International Journal of the Care of the Injured. 2014;45(1):23-30.

584. Kuhlman MB, Lohse N, Sorensen AM, Larsen CF, Christensen KB, Steinmetz J. Impact of the severity of trauma on early retirement. Injury-International Journal of the Care of the Injured. 2014;45(3):618-23.

585. Langlo NM, Orvik AB, Dale J, Uleberg O, Bjornsen LP. The acute sick and injured patients: an overview of the emergency department patient population at a Norwegian University Hospital Emergency Department. European Journal of Emergency Medicine. 2014;21(3):175-80.

586. Larsson J, Bjorkdahl A, Esbjornsson E, Sunnerhagen KS. Participation after traumatic brain injury related to sick-leave. Brain Injury. 2014;28 (5-6):733.

587. Laugsand LE, Strand LB, Vatten LJ, Janszky I, Bjorngaard JH. Insomnia Symptoms and Risk for Unintentional Fatal Injuries-The HUNT Study. Sleep. 2014;37(11):1776-88.

588. Linnemann M, Tibaek M, Kammersgaard LP. Hydrocephalus during rehabilitation following severe TBI. Relation to recovery, outcome, and length of stay. Neurorehabilitation. 2014;35(4):755-61.

589. Lovio R, Bergman P, Lonnerblad M, Skalin E, Gunnarson E, Hagberg-Van't Hooft I. Acute and long-term neurorehabilitation: A comprehensive (follow-up) programme for children and young adults with ABI of all severities. Brain Injury. 2014;28 (5-6):760.

590. Lovstad M, Andelic N, Knoph R, Jerstad T, Anke A, Skandsen T, et al. Rate of disorders of consciousness in a prospective population-based study of adults with traumatic brain injury. Brain Injury. 2014;28 (5-6):708.

591. Mikkelsen R, Hansen OM, Brink O. Non-survivors after admission to trauma centre. Danish Medical Journal. 2014;61(10).

592. Mikkelsen R, Moller Hansen O, Brink O. Non-survivors after admission to trauma centre. Danish Medical Journal. 2014;61(10):A4928.

593. Norden C, Hult K, Engstrom A. Ambulance nurses' experiences of nursing critically ill and injured children: A difficult aspect of ambulance nursing care. International Emergency Nursing. 2014;22(2):75-80.

594. Pekkari P, Bylund PO, Lindgren H, Oman M. Abdominal injuries in a low trauma volume hospital--a descriptive study from northern Sweden. Scandinavian journal of trauma, resuscitation and emergency medicine. 2014;22:48.

595. Perheentupa U, Makitie AA, Kinnunen I. Subcranial craniotomy approach for frontobasal fracture correction. Journal of Cranio-Maxillofacial Surgery. 2014;42(7):1371-7.

596. Roe C, Godbolt A, Deboussard CN, Kammersgaard LP, Nielsen JF, Borg J, et al. Organization of rehabilitation and outcome after severe traumatic brain injury in the Scandinavian countries. Brain Injury. 2014;28 (5-6):570.

597. Rognas L, Hansen TM, Kirkegaard H, Tonnesen E. Anaesthesiologist-provided prehospital airway management in patients with traumatic brain injury: an observational study. European Journal of Emergency Medicine. 2014;21(6):418-23.

598. Samant Y, Gravseth HM, Lund J, Wergeland E. Epidemiological surveillance of work-related injuries in Norway: An enduring challenge. Occupational and Environmental Medicine. 2014;71:A28.

599. Skattum J, Gaarder C, Naess PA. Splenic artery embolisation in children and adolescents--an 8 year experience. Injury. 2014;45(1):160-3.

600. Staff T, Eken T, Wik L, Roislien J, Sovik S. Physiologic, demographic and mechanistic factors predicting New Injury Severity Score (NISS) in motor vehicle accident victims. Injury-International Journal of the Care of the Injured. 2014;45(1):9-15.

601. Steinmetz J. Impact of the severity of trauma on early retirement. Injury. 2014;45(3):618-23.

602. Stenberg M, Koskinen LO, Levi R, Jonasson P, Stalnacke BM. Severe traumatic brain injury patients in Northern Sweden computed tomography and clinical outcomes. Brain Injury. 2014;28 (5-6):593.

603. Stenhagen M, Ekstrom H, Nordell E, Elmstahl S. Accidental falls, health-related quality of life and life satisfaction: A prospective study of the general elderly population. Archives of Gerontology and Geriatrics. 2014;58(1):95-100.

604. Stubbs PW, Pallesen H, Pedersen AR, Nielsen JF. Using EFA and FIM rating scales could provide a more complete assessment of patients with acquired brain injury. Disability and Rehabilitation. 2014;36(26):2278-81.

605. Thelin EP, Nelson DW, Bellander BM. Secondary peaks of S100B in serum relate to subsequent radiological pathology in traumatic brain injury. Neurocritical Care. 2014;20(2):217-29.

606. Uleberg O, Vinjevoll O-P, Kristiansen T, Klepstad P. Norwegian trauma care: a national cross-sectional survey of all hospitals involved in the management of major trauma patients. Scandinavian Journal of Trauma Resuscitation & Emergency Medicine. 2014;22.

607. Ulfarsson T, Lundgren-Nilsson A, Blomstrand C, Jakobsson K-E, Oden A, Nilsson M, et al. Ten-year mortality after severe traumatic brain injury in western Sweden: A case control study. Brain Injury. 2014;28(13-14):1675-81.

608. Ulfarsson T, Lundgren-Nilsson A, Blomstrand C, Nilsson M. A history of unemployment or sick leave influences long-term functioning and health-related quality-of-life after severe traumatic brain injury. Brain Injury. 2014;28(3):328-35.

609. Vaaramo K, Puljula J, Tetri S, Juvela S, Hillbom M. Predictors of new-onset seizures: a 10-year follow-up of head trauma subjects with and without traumatic brain injury. Journal of Neurology Neurosurgery and Psychiatry. 2014;85(6):598-602.

610. Wallin BG, Karlsson T, Pegenius G, Karlsson AK, MacEfield VG, Elam M. Sympathetic single axonal discharge after spinal cord injury in humans: Activity at rest and after bladder stimulation. Spinal Cord. 2014;52(6):434-8.

611. Wisborg T, Bjerkan B. Air ambulance nurses as expert supplement to local emergency services. Air Medical Journal. 2014;33(1):40-3.

612. Anke A, Andelic N, Skandsen T, Knoph R, Ader T, Manskow U, et al. Functional Recovery and Life Satisfaction in the First Year After Severe Traumatic Brain Injury: A Prospective Multicenter Study of a Norwegian National Cohort. Journal of Head Trauma Rehabilitation. 2015;30(4):E38-E49.

613. Austdal LE, Strand MC, Vindenes V, Bogstrand ST. [Prevalence of injuries associated with the <<russ>> celebration in Norway]. Tidsskrift for Den Norske Laegeforening. 2015;135(7):648-52.

614. Bogstrand ST, Hoiseth G, Rossow I, Normann PT, Ekeberg O. Prevalence of ethyl glucuronide and ethyl sulphate among patients injured when driving or at work. Alcohol and alcoholism (Oxford, Oxfordshire). 2015;50(1):68-73.

615. Brennvall H-M, Hauken H, Hunskår S, Wisborg T, Zakariassen E. Out-of-hours doctors' decisions on call-outs in emergency situations. Tidsskrift for den Norske Laegeforening. 2015;135(7):654-7.

616. Buendia R, Candefjord S, Fagerlind H, Balint A, Sjoqvist BA. On scene injury severity prediction (OSISP) algorithm for car occupants. Accident Analysis and Prevention. 2015;81:211-7.

617. Dehli T, Gaarder T, Christensen BJ, Vinjevoll OP, Wisborg T. Implementation of a trauma system in Norway: a national survey. Acta Anaesthesiologica Scandinavica. 2015;59(3):384-91.

618. Dybdal B, Svane C, Hesselfeldt R, Steinmetz J, Sorensen AM, Rasmussen LS. Is there a diurnal difference in mortality of severely injured trauma patients? Emergency Medicine Journal. 2015;32(4):287-90.

619. Falk A-C, Alm A, Lindstrom V. y Prehospital management of traumatic brain injury patients - a gender perspective. International Emergency Nursing. 2015;23(3):250-3.

620. Godbolt AK, Stenberg M, Lindgren M, Ulfarsson T, Lannsjo M, Stalnacke BM, et al. Associations between care pathways and outcome 1 year after severe traumatic brain injury. Journal of Head Trauma Rehabilitation. 2015;30(3):E41-E51.

621. Herou E, Romner B, Tomasevic G. Acute Traumatic Brain Injury: Mortality in the Elderly. World Neurosurgery. 2015;83(6):996-1001.

622. Holmström B, Alhbin S, Pazooki D, Granhed H. 58 people with bullet wounds in Gothenburg during 18 months. This demonstrates the need for preparedness and competence within trauma care. Läkartidningen. 2015;112(29-31):1284-7.

623. Klose M, Stochholm K, Janukonyte J, Christensen LL, Cohen AS, Wagner A, et al. Patient reported outcome in posttraumatic pituitary deficiency: results from The Danish National Study on posttraumatic hypopituitarism. Eur. 2015;172(6):753-62.

624. Lantto M, Renko M, Uhari M. Regional differences in postneonatal childhood mortality in Finland, 1985-2004. Acta Paediatrica. 2015;104(5):466-72.

625. Mikkelsen S, Krueger AJ, Zwisler ST, Brochner AC. Outcome following physician supervised prehospital resuscitation: a retrospective study. Bmj Open. 2015;5(1).

626. Molero Y, Lichtenstein P, Zetterqvist J, Gumpert CH, Fazel S. Varenicline and risk of psychiatric conditions, suicidal behaviour, criminal offending, and transport accidents and offences: Population based cohort study. BMJ (Online). 2015;350(h2388).

627. Nichol A, French C, Little L, Presneill J, Cooper DJ, Haddad S, et al. Erythropoietin in traumatic brain injury: study protocol for a randomised controlled trial. Trials. 2015;16(1):528-.

628. Pedersen K, Fahlstedt M, Jacobsson A, Kleiven S, von Holst H. A National Survey of Traumatic Brain Injuries Admitted to Hospitals in Sweden from 1987 to 2010. Neuroepidemiology. 2015;45(1):20-7.

629. Pickering A, Cooper K, Harnan S, Sutton A, Mason S, Nicholl J. Impact of prehospital transfer strategies in major trauma and head injury: Systematic review, meta-analysis, and recommendations for study design. Journal of Trauma and Acute Care Surgery. 2015;78(1):164-77.

630. Samuelsson C, Sjoberg F, Karlstrom G, Nolin T, Walther SM. Gender differences in outcome and use of resources do exist in Swedish intensive care, but to no advantage for women of premenopausal age. Critical Care. 2015;19(1).

631. Steuer J, Bjorck M, Sonesson B, Resch T, Dias N, Hultgren R, et al. Editor's Choice - Durability of Endovascular Repair in Blunt Traumatic Thoracic Aortic Injury: Long-Term Outcome from Four Tertiary Referral Centers. European journal of vascular and endovascular surgery : the official journal of the European Society for Vascular Surgery. 2015;50(4):460-5.

632. Stordahl H, Passas E, Hopland A, Nielsen EW. Nine out of ten trauma calls to a Norwegian hospital are avoidable: A retrospective analysis. BMC Emergency Medicine. 2015;15(1).

633. Zakariassen E, Uleberg O, Roislien J. Helicopter emergency medical services response times in Norway: Do they matter? Air Medical Journal. 2015;34(2):98-103.

634. Alstrom HB, Pedersen NE, Moller AM. Clinical findings leading to pre-hospital intubation. Acta Anaesthesiologica Scandinavica. 2015;121):21-2.

635. Alstrom HB, Pedersen NE, Moller AM. Pre-hospital interventions and on-scene time. Acta Anaesthesiologica Scandinavica. 2015;121):21.

636. Austdal LE, Strand MC, Vindenes V, Bogstrand ST. Prevalence of injuries associated with the Norway russ-celebration. Tidsskr Nor Laegeforen. 2015;135(7):648-52.

637. Bagher A, Andersson L, Wingren CJ, Ottosson A, Wangefjord S, Acosta S. Outcome after red trauma alarm at an urban Swedish hospital: Implications for prevention. Scandinavian Journal of Public Health. 2015;43(5):506-13.

638. Bagher A, Wingren CJ, Ottosson A, Andersson L, Wangefjord S, Acosta S. Necessity of including medico-legal autopsy data in epidemiological surveys of individuals with major trauma. Injury-International Journal of the Care of the Injured. 2015;46(8):1515-9.

639. Bakke HK, Steinvik T, Eidissen SI, Gilbert M, Wisborg T. Bystander first aid in trauma - prevalence and quality: a prospective observational study. Acta Anaesthesiologica Scandinavica. 2015;59(9):1187-93.

640. Bjoernaa E, Hammer NR, Ponten E, Valkonen H. Current pediatric transfusion strategies in the Nordic countries. Acta Anaesthesiologica Scandinavica. 2015;121):12.

641. Bjornsdottir E, Sigvaldason K, Sigurdsson MI, Moller AD, Blondal A, Sigurdsson GH. The epidemiology of mechanical ventilation in Iceland. Acta Anaesthesiologica Scandinavica. 2015;121):43.

642. Brinck T, Handolin L, Paffrath T, Lefering R. Trauma registry comparison: six-year results in trauma care in Southern Finland and Germany. European Journal of Trauma and Emergency Surgery. 2015;41(5):509-16.

643. Brommeland T, Rydning PN, Pripp AH, Helseth E. Cranioplasty complications and risk factors associated with bone flap resorption. Scandinavian Journal of Trauma Resuscitation & Emergency Medicine. 2015;23.

644. Buendia R, Candefjord S, Fagerlind H, Balint A, Sjoqvist BA. On scene injury severity prediction (OSISP) algorithm for car occupants. Accid Anal Prev. 2015;81:211-7.

645. Candefjord S, Buendia R, Fagerlind H, Balint A, Wege C, Sjoqvist BA. On-Scene Injury Severity Prediction (OSISP) Algorithm for Truck Occupants. Traffic Injury Prevention. 2015;16:S190-S6.

646. Cherpitel CJ, Ye Y, Bond J, Borges G, Monteiro M, Chou P, et al. Alcohol Attributable Fraction for Injury Morbidity from the Dose-Response Relationship of Acute Alcohol Consumption: Emergency Department Data from 18 Countries. Addiction. 2015;110(11):1724-32.

647. Dalsgaard S, Leckman JF, Mortensen PB, Nielsen HS, Simonsen M. Effect of drugs on the risk of injuries in children with attention deficit hyperactivity disorder: A prospective cohort study. The Lancet Psychiatry. 2015;2(8):702-9.

648. Dehli T, Bagenholm A, Trasti NC, Monsen SA, Bartnes K. The treatment of spleen injuries: a retrospective study. Scand J Trauma Resusc Emerg Med. 2015;23:85.

649. Doohan I, Saveman BI. Need for compassion in prehospital and emergency care: a qualitative study on bus crash survivors' experiences. International emergency nursing. 2015;23(2):115-9.

650. Duffy JR, Warburg FE, Koelle SFT, Werner MU, Nielsen PR. Pain-related psychological distress, self-rated health and significance of neuropathic pain in Danish soldiers injured in Afghanistan. Acta Anaesthesiologica Scandinavica. 2015;59(10):1367-76.

651. Dybdal B, Svane C, Hesselfeldt R, Steinmetz J, Sorensen AM, Rasmussen LS. Is there a diurnal difference in mortality of severely injured trauma patients? Emerg Med J. 2015;32(4):287-90.

652. Eriksson M, Brattstrom O, Martensson J, Larsson E, Oldner A. Acute kidney injury following severe trauma: Risk factors and long-term outcome. J Trauma Acute Care Surg. 2015;79(3):407-12.

653. Falk A-C, Alm A, Lindstrom V. Prehospital management of traumatic brain injury patients - a gender perspective. International Emergency Nursing. 2015;23(3):250-3.

654. Fattah S, Agledahl KM, Rehn M, Wisborg T. Implementation of a global open access template for reporting pre-hospital major incident medical management. Acta Anaesthesiologica Scandinavica. 2015;121):28.

655. Godbolt AK, Stenberg M, Lindgren M, Ulfarsson T, Lannsjo M, Stalnacke BM, et al. Associations between care pathways and outcome 1 year after severe traumatic brain injury. J Head Trauma Rehabil. 2015;30(3):E41-51.

656. Gunnarsson B, Jensen NSK, Tummas i G, Hardardottir H, Stefansdottir L, Heimisdottir M. Air ambulance and hospital services for critically ill and injured in Greenland, Iceland and the Faroe Islands: how can we improve? Int J Circumpolar Health. 2015;74.

657. Gyllencreutz L, Rolfsman E, Saveman B-I. Non-minor injuries among children sustained in an outdoor environment - a retrospective register study. International Journal of Injury Control and Safety Promotion. 2015;22(1):3-10.

658. Holmstrom B, Alhbin S, Pazooki D, Granhed H. [58 people with bullet wounds in Gothenburg during 18 months. This demonstrates the need for preparedness and competence within trauma care]. Lakartidningen. 2015;112.

659. Holzmann MJ, Bylund PO, Degerfält L, Carlsson AC, Wändell P, Ruge T. Longer waiting time and higher mortality in older people with traumatic brain injuries. Mapping of emergency prehospital management and hospital management in Västerbotten. Läkartidningen. 2015;112(41):1784-8.

670. Inkinen J, Kirjasuo K, Gunn J, Kuttila K. Penetrating trauma; experience from Southwest Finland between 1997 and 2011, a retrospective descriptive study. Eur. 2015;41(4):429-33.

671. Jaldell H. How important is the time factor? Saving lives using fire and rescue services. Value in Health. 2015;18 (7):A535.

672. Jaldell H, Ryen L, Sund B, Andersson R. Are national injury prevention and research efforts matching the distribution of injuries across sectors? Inj Prev. 2015;21(E1):E113-E5.

673. Jorgensen TSH, Hansen AH, Sahlberg M, Gislason GH, Torp-Pedersen C, Andersson C, et al. Nationwide time trends and risk factors for in-hospital falls-related major injuries. International Journal of Clinical Practice. 2015;69(6):703-9.

674. Kaljusto M-L, Skaga NO, Pillgram-Larsen J, Tonnessen T. Survival predictor for penetrating cardiac injury; a 10-year consecutive cohort from a scandinavian trauma center. Scandinavian Journal of Trauma Resuscitation & Emergency Medicine. 2015;23.

675. Kirkegard J, Avlund TH, Amanavicius N, Mortensen FV, Kissmeyer-Nielsen P. Non-operative management of blunt splenic injuries in a paediatric population: a 12-year experience. Danish medical journal. 2015;62(2).

676. Knudsen A, Hebsgaard S, Zwisler ST. Trauma and analgesics-a retrospective pre-hospital evaluation. Resuscitation. 2015;1):157.

677. Manskow US, Sigurdardottir S, Roe C, Andelic N, Skandsen T, Damsgard E, et al. Factors Affecting Caregiver Burden 1 Year After Severe Traumatic Brain Injury: A Prospective Nationwide Multicenter Study. J Head Trauma Rehabil. 2015;30(6):411-23.

678. Mohseni S, Talving P, Thelin EP, Wallin G, Ljungqvist O, Riddez L. The Effect of beta-blockade on Survival After Isolated Severe Traumatic Brain Injury. World Journal of Surgery. 2015;39(8):2076-83.

679. Moller TP, Ersboll AK, Tolstrup JS, Ostergaard D, Viereck S, Overton J, et al. Why and when citizens call for emergency help: an observational study of 211,193 medical emergency calls. Scandinavian Journal of Trauma Resuscitation & Emergency Medicine. 2015;23.

680. Norup A, Perrin PB, Cuberos-Urbano G, Anke A, Andelic N, Doyle ST, et al. Family needs after brain injury: A cross cultural study. Neurorehabilitation. 2015;36(2):203-14.

681. Oroy A, Stromskag KE, Gjengedal E. Do we treat individuals as patients or as potential donors? A phenomenological study of healthcare professionals' experiences. Nursing Ethics. 2015;22(2):163-75.

682. Oyo-Ita A, Chinnock P, Ikpeme IA. Surgical versus non-surgical management of abdominal injury. Cochrane Database of Systematic Reviews. 2015(11):CD007383.

683. Pedersen K, Fahlstedt M, Jacobsson A, Kleiven S, von Holst H. A National Survey of Traumatic Brain Injuries Admitted to Hospitals in Sweden from 1987 to 2010. Neuroepidemiology. 2015;45(1):20-7.

684. Pensbo-Madsen T, Anderson K, Lucantoni C, Babu R, Gehrchen M, Biering-Sorensen F, et al. The epidemiology of surgically treated spinal fractures in Eastern Denmark. European Spine Journal. 2015;1):S779.

685. Poyhonen R, Suominen V, Uurto I, Salenius J. Non-iatrogenic civilian vascular trauma in a well-defined geographical region in Finland. European Journal of Trauma and Emergency Surgery. 2015;41(5):545-9.

686. Remes H, Martikainen P. Young adult's own and parental social characteristics predict injury morbidity: a register-based follow-up of 135 000 men and women. Bmc Public Health. 2015;15.

687. Rimstad R, Holtan A. A cross-sectional survey of patient needs in hospital evacuation. Journal of emergency management (Weston, Mass). 2015;13(4):295-301.

688. Rimstad R, Sollid SJM. A retrospective observational study of medical incident command and decision-making in the 2011 Oslo bombing. International Journal of Emergency Medicine. 2015;8(1).

689. Roaldsen KS, Wakefield E, Jorgensen V, Opheim A. Pragmatic evaluation of aspects concerning validity and feasibility of the mini balance evaluation system test in a specialized rehabilitation setting. Physiotherapy (United Kingdom). 2015;1):eS694-eS5.

690. Roding F, Lindkvist M, Bergstrom U, Lysholm J. Epidemiologic patterns of injuries treated at the emergency department of a Swedish medical center. Injury epidemiology. 2015;2(1):3-.

691. Roe C, Skandsen T, Manskow U, Ader T, Anke A. Mortality and One-Year Functional Outcome in Elderly and Very Old Patients with Severe Traumatic Brain Injuries: Observed and Predicted. Behavioural Neurology. 2015;2015:845491.

692. Roislien J, Lossius HM, Kristiansen T. Does transport time help explain the high trauma mortality rates in rural areas? New and traditional predictors assessed by new and traditional statistical methods. Inj Prev. 2015;21(6):367-73.

693. Raatiniemi L, Liisanantti J, Niemi S, Nal H, Ohtonen P, Antikainen H, et al. Short-term outcome and differences between rural and urban trauma patients treated by mobile intensive care units in Northern Finland: a retrospective analysis. Scandinavian Journal of Trauma Resuscitation & Emergency Medicine. 2015;23.

694. Raatiniemi LV, Steinvik T, Wisborg T, Martikainen M, Liisanantti J, Alahuhta S, et al. Fatal injuries in Northern Finland-pre-hospital death dominates. Acta Anaesthesiologica Scandinavica. 2015;121):27.

695. Sandhaug M, Andelic N, Langhammer B, Mygland A. Community integration 2 years after moderate and severe traumatic brain injury. Brain Injury. 2015;29(7-8):915-20.

696. Soderlund T, Ikonen A, Pyhalto T, Handolin L. Factors Associated with in-Hospital Outcomes in 594 Consecutive Patients Suffering from Severe Blunt Chest Trauma. Scandinavian Journal of Surgery. 2015;104(2):115-20.

697. Stenberg M, Godbolt AK, Nygren De Boussard C, Levi R, Stalnacke BM. Cognitive Impairment after Severe Traumatic Brain Injury, Clinical Course and Impact on Outcome: A Swedish-Icelandic Study. Behavioural Neurology. 2015;2015:680308.

698. Steuer J, Bjorck M, Sonesson B, Resch T, Dias N, Hultgren R, et al. Editor's Choice - Durability of Endovascular Repair in Blunt Traumatic Thoracic Aortic Injury: Long-Term Outcome from Four Tertiary Referral Centers. Eur J Vasc Endovasc Surg. 2015;50(4):460-5.

699. Sveen U, Andelic N, Bautz-Holter E, Roe C. Self-reported competency - validation of the Norwegian version of the patient competency rating scale for traumatic brain injury. Disability and Rehabilitation. 2015;37(3):239-46.

700. Thomsen L, Lindholt J, Roeder O, Green A, Mikkelsen RT. Excess mortality during the first year after arterial trauma. Danish Medical Journal. 2015;62(3).

701. Tran TT, Goss B, Bjarnason H, Skaga NO, Roy S, Leonard J, et al. Prevention of pulmonary embolism in patients with severe trauma: Role of IVC filters. CardioVascular and Interventional Radiology. 2015;1):S189.

702. Vaaramo K, Puljula J, Tetri S, Juvela S, Hillbom M. Head Trauma with or without Mild Brain Injury Increases the Risk of Future Traumatic Death: A Controlled Prospective 15-Year Follow-Up Study. Journal of Neurotrauma. 2015;32(20):1579-83.

703. Wisborg T, Ellensen EN, Svege I, Dehli T. Dispatch precision of advanced prehospital treatment for severely injured trauma victims in Norway-a national study. Acta Anaesthesiologica Scandinavica. 2015;121):28-9.

704. Wisborg T, Ellensen EN, Svege I, Dehli T. A national trauma system? A retrospective assessment of the flow of severely injured patients in Norway 2013. Acta Anaesthesiologica Scandinavica. 2015;121):24.

705. Zibung E, Riddez L, Nordenvall C. Helmet use in bicycle trauma patients: a population-based study. Eur. 2015;41(5):517-21.

706. Zwisler ST, Hallas J, Larsen MS, Handberg G, Mikkelsen S, Enggaard TP. Opioid prescriptions before and after high-energy trauma. Journal of opioid management. 2015;11(4):313-8.

707. Adelved A, Totterman A, Glott T, Hellund JC, Madsen JE, Roise O. Long-term functional outcome after traumatic lumbosacral dissociation. A retrospective case series of 13 patients. Injury. 2016;47(7):1562-8.

708. Airaksinen N, Nurmi-Luthje I, Luthje P. Comparison of Injury Severity Between Moped and Motorcycle Crashes: A Finnish Two-Year Prospective Hospital-Based Study. Scandinavian Journal of Surgery. 2016;105(1):49-55.

709. Ala-Seppala H, Heino I, Frantzen J, Takala RSK, Katila AJ, Kyllonen A, et al. Injury profiles, demography and representativeness of patients with TBI attending a regional emergency department. Brain Injury. 2016;30(9):1062-7.

709. Andelic N, Arango-Lasprilla JC, Perrin PB, Sigurdardottir S, Lu J, Landa LO, et al. Modeling of Community Integration Trajectories in the First Five Years after Traumatic Brain Injury. Journal of Neurotrauma. 2016;33(1):95-100.

710. Andersen MS, Christensen EF, Jepsen SB, Nortved J, Hansen JB, Johnsen SP. Can public health registry data improve Emergency Medical Dispatch? Acta Anaesthesiologica Scandinavica. 2016;60(3):370-9.

711. Anke A, Manskow US, Friborg O, Roe C, Arntzen C. The family experiences of in-hospital care questionnaire in severe traumatic brain injury (FECQ-TBI): a validation study. Bmc Health Services Research. 2016;16.

712. Aro E, Jaaskelainen JE, Reinikainen M, Leinonen V, Hakala T. [Emergency craniotomies at the North Karelia Central Hospital in 2006 to 2014]. Duodecim; laaketieteellinen aikakauskirja. 2016;132(9):858-65.

713. Bagher A, Andersson L, Wingren CJ, Ottosson A, Wangefjord S, Acosta S. Socio-economic status and major trauma in a Scandinavian urban city: A population-based case-control study. Scandinavian Journal of Public Health. 2016;44(2):217-23.

714. Bakke E, Bogstrand ST, Normann PT, Ekeberg O, Bachs L. Influence of alcohol and other substances of abuse at the time of injury among patients in a Norwegian emergency department. Bmc Emergency Medicine. 2016;16.

715. Bernhoff K, Bjorck M, Larsson J, Jangland E. Patient Experiences of Life Years After Severe Civilian Lower Extremity Trauma With Vascular Injury. European Journal of Vascular and Endovascular Surgery. 2016;52(5):690-5.

716. Brazinova A, Rehorcikova V, Taylor MS, Buckova V, Majdan M, Psota M, et al. Epidemiology of Traumatic Brain Injury in Europe: A Living Systematic Review. Journal of Neurotrauma. 2016.

717. Brinck T, Handolin L, Lefering R. The Effect of Evolving Fluid Resuscitation on the Outcome of Severely Injured Patients: An 8-year Experience at a Tertiary Trauma Center. Scandinavian Journal of Surgery. 2016;105(2):109-16.

718. Brinck T, Raj R, Skrifvars MB, Kivisaari R, Siironen J, Lefering R, et al. Unconscious trauma patients: outcome differences between southern Finland and Germany-lesson learned from trauma-registry comparisons. European Journal of Trauma and Emergency Surgery. 2016;42(4):445-51.

719. Bylund P-O, Johansson J, Albertsson P. Injuries sustained during snow removal from roofs resulting in hospital care. International Journal of Injury Control and Safety Promotion. 2016;23(1):105-9.

720. Candefjord S, Buendia R, Caragounis E-C, Sjoqvist BA, Fagerlind H. Prehospital transportation decisions for patients sustaining major trauma in road traffic crashes in Sweden. Traffic Injury Prevention. 2016;17:16-20.

721. Christensen EF, Larsen TM, Jensen FB, Bendtsen MD, Hansen PA, Johnsen SP, et al. Diagnosis and mortality in prehospital emergency patients transported to hospital: a population-based and registry-based cohort study. Bmj Open. 2016;6(7).

722. Eriksson M, Brattstrom O, Larsson E, Oldner A. Causes of excessive late death after trauma compared with a matched control cohort. British Journal of Surgery. 2016;103(10):1282-9.

723. Funder KS, Rasmussen LS, Lohse N, Siersma V, Hesselfeldt R, Steinmetz J. Long-term follow-up of trauma patients before and after implementation of a physician-staffed helicopter: A prospective observational study. Injury-International Journal of the Care of the Injured. 2016;47(1):7-13.

724. Gaski IA, Barckman J, Naess PA, Skaga NO, Madsen JE, Klow NE, et al. Reduced need for extraperitoneal pelvic packing for severe pelvic fractures is associated with improved resuscitation strategies. Journal of Trauma and Acute Care Surgery. 2016;81(4):644-51.

725. Ghorbani P, Ringdal KG, Hestnes M, Skaga NO, Eken T, Ekbom A, et al. Comparison of risk-adjusted survival in two Scandinavian Level-I trauma centres. Scandinavian Journal of Trauma Resuscitation & Emergency Medicine. 2016;24.

726. Hargestam M, Lindkvist M, Jacobsson M, Brulin C, Hultin M. Trauma teams and time to early management during in situ trauma team training. Bmj Open. 2016;6(1).

727. Hart T, Whyte J, Poulsen I, Kristensen KS, Nordenbo AM, Chervoneva I, et al. How Do Intensity and Duration of Rehabilitation Services Affect Outcomes From Severe Traumatic Brain Injury? A Natural Experiment Comparing Health Care Delivery Systems in 2 Developed Nations. Archives of Physical Medicine and Rehabilitation. 2016;97(12):2045-53.

728. Harve H, Salmi H, Rahiala E, Pohjalainen P, Kuisma M. Out-of-hospital paediatric emergencies: a prospective, population-based study. Acta Anaesthesiologica Scandinavica. 2016;60(3):360-9.

729. Hasselbalch RB, Plesner LL, Pries-Heje M, Ravn L, Lind M, Greibe R, et al. The Copenhagen Triage Algorithm: a randomized controlled trial. Scandinavian Journal of Trauma Resuscitation & Emergency Medicine. 2016;24.

730. Hernefalk B, Eriksson N, Borg T, Larsson S. Estimating pre-traumatic quality of life in patients with surgically treated acetabular fractures and pelvic ring injuries: Does timing matter? Injury. 2016;47(2):389-94.

731. Hoikka M, Lankimaki S, Silfvast T, Ala-Kokko TI. Medical priority dispatch codes-comparison with National Early Warning Score. Scandinavian Journal of Trauma Resuscitation & Emergency Medicine. 2016;24.

732. Holgersson A, Sahovic D, Saveman B-I, Bjornstig U. Factors influencing responders' perceptions of preparedness for terrorism. Disaster Prevention and Management. 2016;25(4):520-33.

733. Husebo SE, Olsen OE. Impact of clinical leadership in teams' course on quality, efficiency, responsiveness and trust in the emergency department: Study protocol of a trailing research study. BMJ Open. 2016;6 (8) (no pagination)(e011899).

734. Irgens EL, Henriksen N, Moe S. Acquired brain injury rehabilitation: dilemmas in neurological physiotherapy across healthcare settings. European Journal of Physiotherapy. 2016;18(4):202-9.

735. Janstrup KH, Kaplan S, Hels T, Lauritsen J, Prato CG. Understanding traffic crash under-reporting: Linking police and medical records to individual and crash characteristics. Traffic Injury Prevention. 2016;17(6):580-4.

736. Jensen JF, Egerod I, Bestle MH, Christensen DF, Elklit A, Hansen RL, et al. A recovery program to improve quality of life, sense of coherence and psychological health in ICU survivors: a multicenter randomized controlled trial, the RAPIT study. Intensive Care Medicine. 2016;42(11):1733-43.

737. Jonsson A, Larusson SH, Mogensen A, Bjornsson HM, Mogensen BA. Incidence of Bicycle injuries presenting to the Emergency Department in Reykjavik 2005-2010. [Icelandic]. Laeknabladid. 2016;102(2):77-82.

738. Jorgensen JJ, Naess PA, Gaarder C. Injuries caused by fragmenting rifle ammunition. Injury-International Journal of the Care of the Injured. 2016;47(9):1951-4.

739. Kivipuro TJ, Wiksten JK. Overview of the use of blood products in the hospital district of helsinki and uusimaa in 2011-2014. Transfusion. 2016;56 (Supplement 4):171A.

740. Kosola S, Salminen P, Kallio P. Driver's education may reduce annual incidence and severity of moped and scooter accidents. A population-based study. Injury-International Journal of the Care of the Injured. 2016;47(1):239-43.

741. Kristinsdottir EA, Knutsdottir S, Sigvaldason K, Jonsson H, Ingvarsson PE. Epidemiology of Spinal Cord Injury in Iceland from 1975 to 2014. [Icelandic]. Laeknabladid. 2016;102(11):491-6.

742. Kristoffersen S, Normann S-A, Morild I, Lilleng PK, Heltne J-K. The hazard of sharp force injuries: Factors influencing outcome. Journal of Forensic and Legal Medicine. 2016;37:71-7.

743. Lander F, Nielsen KJ, Lauritsen J. Work injury trends during the last three decades in the construction industry. Safety Science. 2016;85:60-6.

744. Lu J, Roe C, Sigurdardottir S, Forslund M, Andelic N. Five-year functional outcomes following moderate and severe traumatic brain injury. Brain Injury. 2016;30 (5-6):608-9.

745. Luef SM, Lauritsen JM, Faergemann C. Trends in weapon-related injuries from violence in Odense Municipality, Denmark 1991-2009. Danish medical journal. 2016;63(11).

746. Maribo T, Pedersen AR, Jensen J, Nielsen JF. Assessment of primary rehabilitation needs in neurological rehabilitation: translation, adaptation and face validity of the Danish version of Rehabilitation Complexity Scale-Extended. BMC Neurol. 2016;16(1):205.

747. McKinlay A, Linden M, DePompei R, Aaro Jonsson C, Anderson V, Braga L, et al. Service provision for children and young people with acquired brain injury: Practice recommendations. Brain Injury. 2016;30(13-14):1656-64.

748. Mehus G, Mehus AG, Germeten S, Henriksen N. Young people and snowmobiling in northern Norway: accidents, injury prevention and safety strategies. Rural and Remote Health. 2016;16(4).

749. Mohseni S, Bellander B-M, Riddez L, Talving P, Thelin EP. Positive blood alcohol level in severe traumatic brain injury is associated with better long-term functional outcome. Brain Injury. 2016;30(10):1256-60.

750. Narvestad JK, Meskinfamfard M, Soreide K. Emergency resuscitative thoracotomy performed in European civilian trauma patients with blunt or penetrating injuries: a systematic review. European Journal of Trauma and Emergency Surgery. 2016;42(6):677-85.

751. Nordberg M, Castren M, Lindstrom V. Primary Trauma Triage Performed by Bystanders: An Observation Study. Prehospital and Disaster Medicine. 2016;31(4):353-7.

752. Oesterlund AH, Lander F, Lauritsen J. A case-crossover study of transient risk factors influence on occupational injuries: a study protocol based on a review of previous studies. Inj Prev. 2016;22(5):375.

753. Ohrt-Nissen S, Colville-Ebeling B, Kandler K, Hornbech K, Steinmetz J, Ravn J, et al. Indication for resuscitative thoracotomy in thoracic injuries-Adherence to the ATLS guidelines. A forensic autopsy based evaluation. Injury-International Journal of the Care of the Injured. 2016;47(5):1019-24.

754. Oman M, Fredriksson R, Bylund P-O, Bjornstig U. Analysis of the mechanism of injury in non-fatal vehicle-to-pedestrian and vehicle-to-bicyclist frontal crashes in Sweden. International Journal of Injury Control and Safety Promotion. 2016;23(4):405-12.

755. Osteras O, Brattebo G, Heltne JK. Helicopter-based emergency medical services for a sparsely populated region: A study of 42,500 dispatches. Acta Anaesthesiologica Scandinavica. 2016;60(5):659-67.

756. Puljula J, Vaaramo K, Tetri S, Juvela S, Hillbom M. Risk for All-cause and Traumatic Death in Head Trauma Subjects A Prospective Population-based Case-control Follow-up Study. Annals of Surgery. 2016;263(6):1235-9.

757. Radestad M, Montan KL, Ruter A, Castren M, Svensson L, Gryth D, et al. Attitudes Towards and Experience of the Use of Triage Tags in Major Incidents: A Mixed Method Study. Prehospital and Disaster Medicine. 2016;31(4):376-85.

758. Raj R, Bendel S, Reinikainen M, Hoppu S, Luoto T, Ala-Kokko T, et al. Traumatic brain injury patient volume and mortality in neurosurgical intensive care units: a Finnish nationwide study. Scandinavian Journal of Trauma Resuscitation & Emergency Medicine. 2016;24.

759. Raj R, Brinck T, Skrifvars MB, Handolin L. External validation of the Norwegian survival prediction model in trauma after major trauma in Southern Finland. Acta Anaesthesiologica Scandinavica. 2016;60(1):48-58.

760. Rehn M, Hyldmo PK, Magnusson V, Kurola J, Kongstad P, Rognas L, et al. Scandinavian SSAI clinical practice guideline on pre-hospital airway management. Acta Anaesthesiologica Scandinavica. 2016;60(7):852-64.

761. Ribbe E, Riddez L, Örtenwall P. Trauma investigation - a help to secure good care of the seriously injured patient. Svensk Kirurgi. 2016;74(2):74-6.

762. Robertsen A, Forde R, Skaga NO, Helseth E. Treatment-limiting-decisions in patients with severe traumatic brain injury in a Norwegian trauma hospital. Intensive Care Medicine Experimental Conference: 29th Annual Congress of the European Society of Intensive Care Medicine, ESICM. 2016;4(Supplement 1).

763. Rosenstock CV, Norskov AK, Wetterslev J, Lundstrom LH. Emergency surgical airway management in Denmark: A cohort study of 452 461 patients registered in the Danish Anaesthesia Database. British Journal of Anaesthesia. 2016;117(Supplement 1):i75-i82.

764. Rubenson Wahlin R, Ponzer S, Lovbrand H, Skrivfars M, Lossius HM, Castren M. Do male and female trauma patients receive the same prehospital care?: An observational follow-up study. BMC Emergency Medicine. 2016;16 (1) (no pagination)(6).

765. Rubenson Wahlin R, Ponzer S, Skrifvars MB, Lossius HM, Castren M. Effect of an organizational change in a prehospital trauma care protocol and trauma transport directive in a large urban city: a before and after study. Scandinavian journal of trauma, resuscitation and emergency medicine. 2016;24:26-.

766. Raaber N, Duvald I, Riddervold I, Christensen EF, Kirkegaard H. Geographic information system data from ambulances applied in the emergency department: effects on patient reception. Scandinavian Journal of Trauma Resuscitation & Emergency Medicine. 2016;24.

767. Raatiniemi L, Steinvik T, Liisanantti J, Ohtonen P, Martikainen M, Alahuhta S, et al. Fatal injuries in rural and urban areas in northern Finland: a 5-year retrospective study. Acta Anaesthesiologica Scandinavica. 2016;60(5):668-76.

768. Sandstrom L, Nilsson C, Juuso P, Engstrom A. Experiences of nursing patients suffering from trauma - preparing for the unexpected: A qualitative study. Intensive and Critical Care Nursing. 2016;36:58-65.

769. Sariaslan A, Sharp DJ, D'Onofrio BM, Larsson H, Fazel S. Long-Term Outcomes Associated with Traumatic Brain Injury in Childhood and Adolescence: A Nationwide Swedish Cohort Study of a Wide Range of Medical and Social Outcomes. Plos Medicine. 2016;13(8).

770. Sveen U, Roe C, Sigurdardottir S, Skandsen T, Andelic N, Manskow U, et al. Rehabilitation pathways and functional independence one year after severe traumatic brain injury. European Journal of Physical and Rehabilitation Medicine. 2016;52(5):650-61.

771. Thelin EP, Jeppsson E, Frostell A, Svensson M, Mondello S, Bellander B-M, et al. Utility of neuron-specific enolase in traumatic brain injury; relations to S100B levels, outcome, and extracranial injury severity. Critical Care. 2016;20.

772. Thelin EP, Zibung E, Riddez L, Nordenvall C. Assessing bicycle-related trauma using the biomarker S100B reveals a correlation with total injury severity. European Journal of Trauma and Emergency Surgery. 2016;42(5):617-25.

773. Thesleff T, Niskakangas T, Luoto TM, Ohman J, Ronkainen A. Fatal cervical spine injuries: a Finnish nationwide register-based epidemiologic study on data from 1987 to 2010. Spine Journal. 2016;16(8):918-26.

774. Trimmel H, Kreutziger J, Fitzka R, Szuts S, Derdak C, Koch E, et al. Use of the glidescope ranger video laryngoscope for emergency intubation in the prehospital setting: A randomized control trial. Critical Care Medicine. 2016;44(7):e470-e6.

775. Turner CDA, Lockey DJ, Rehn M. Pre-hospital management of mass casualty civilian shootings: a systematic literature review. Critical Care. 2016;20.

776. Valimaki M, Korkeila J, Kauppi K, Kaakinen JK, Holm S, Vahlo J, et al. Digital Gaming for Improving the Functioning of People With Traumatic Brain Injury: Protocol of a Feasibility Study. Jmir Research Protocols. 2016;5(1).

777. Weber CD, Horst K, Lefering R, Hofman M, Dienstknecht T, Pape HC. Major trauma in winter sports: an international trauma database analysis. European Journal of Trauma and Emergency Surgery. 2016;42(6):741-7.

778. Wiklund E, Koskinen SK, Linder F, Aslund PE, Eklof H. Whole body computed tomography for trauma patients in the Nordic countries 2014: survey shows significant differences and a need for common guidelines. Acta Radiologica. 2016;57(6):750-7.

779. Zibung E, Riddez L, Nordenvall C. Impaired quality of life after bicycle trauma. Injury. 2016;47(5):1078-82.

780. Ahl R, Lindgren R, Cao Y, Riddez L, Mohseni S. Risk factors for depression following traumatic injury: An epidemiological study from a scandinavian trauma center. Injury-International Journal of the Care of the Injured. 2017;48(5):1082-7.

781. Ahl R, Sjolin G, Mohseni S. Does early beta-blockade in isolated severe traumatic brain injury reduce the risk of post traumatic depression? Injury-International Journal of the Care of the Injured. 2017;48(1):101-5.

782. Andersson E, Rackauskaite D, Svanborg E, Csajbok L, Ost M, Nellgard B. A prospective outcome study observing patients with severe traumatic brain injury over 10-15 years. Acta Anaesthesiologica Scandinavica. 2017;61(5):502-12.

783. Bakke HK, Steinvik T, Angell J, Wisborg T. A nationwide survey of first aid training and encounters in Norway. Bmc Emergency Medicine. 2017;17.

784. Baksaas-Aasen K, Gall L, Eaglestone S, Rourke C, Juffermans NP, Goslings JC, et al. iTACTIC - implementing Treatment Algorithms for the Correction of Trauma-Induced Coagulopathy: study protocol for a multicentre, randomised controlled trial. Trials. 2017;18.

785. Berginstrom N, Nordstrom P, Schuit R, Nordstrom A. The Effects of (-)-OSU6162 on Chronic Fatigue in Patients With Traumatic Brain Injury: A Randomized Controlled Trial. J Head Trauma Rehabil. 2017;32(2):E46-E54.

786. Biering K, Lander F, Rasmussen K. Work injuries among migrant workers in Denmark. Occup Environ Med. 2017;74(4):235-42.

787. Blix SW, Melau J, Lund-Kordahl I. Performance of Norwegian civilian EMTs and army medics in penetrating trauma: a controlled simulation-based assessment. Acta Anaesthesiologica Scandinavica. 2017;61(7):848-53.

789. Bolenius K, Vestin C, Saveman B-I, Gyllencreutz L. Validating a questionnaire - prehospital preparedness for pediatric trauma patients. International Emergency Nursing. 2017;34:2-6.

790. Bonander C, Jernbro C. Does gender moderate the association between intellectual ability and accidental injuries? Evidence from the 1953 Stockholm Birth Cohort study. Accident Analysis and Prevention. 2017;106:109-14.

791. Botker MT, Terkelsen CJ, Sorensen JN, Jepsen SB, Johnsen SP, Christensen EF, et al. Long-Term Mortality of Emergency Medical Services Patients. Annals of Emergency Medicine. 2017;70(3):366-73.e3.

792. Brandrud AS, Bretthauer M, Brattebo G, Pedersen MJB, Hapnes K, Moller K, et al. Local emergency medical response after a terrorist attack in Norway: a qualitative study. Bmj Quality & Safety. 2017;26(10):806-16.

793. Cryer C, Miller TR, Lyons RA, Macpherson AK, Perez K, Petridou ET, et al. Towards valid 'serious non-fatal injury' indicators for international comparisons based on probability of admission estimates. Inj Prev. 2017;23(1):47-57.

794. Doohan I, Bjornstig U, Ostlund U, Saveman BI. Exploring Injury Panorama, Consequences, and Recovery among Bus Crash Survivors: A Mixed-Methods Research Study. Prehospital Disaster Med. 2017;32(2):165-74.

795. Ekstrom DS, Larsen RH, Lauritsen JM, Fargemann C. Children and adolescents admitted to a university-level trauma centre in Denmark 2002-2011. Danish medical journal. 2017;64(4).

796. Fornebo I, Simonsen KA, Bukholm IRK, Kongsgaard UE. Claims for compensation after injuries related to airway management: a nationwide study covering 15 years. Acta Anaesthesiologica Scandinavica. 2017;61(7):781-9.

797. Funder KS, Rasmussen LS, Hesselfeldt R, Siersma V, Lohse N, Sonne A, et al. Quality of life following trauma before and after implementation of a physician-staffed helicopter. Acta Anaesthesiologica Scandinavica. 2017;61(1):111-20.

798. Hackenberg EAM, Sallinen V, Koljonen V, Handolin L. Severe intimate partner violence affecting both young and elderly patients of both sexes. European Journal of Trauma and Emergency Surgery. 2017;43(3):319-27.

799. Henriksson O, Bjornstig U, Saveman BI, Lundgren PJ. Protection against cold - a survey of available equipment in Swedish pre-hospital services. Acta Anaesthesiologica Scandinavica. 2017;61(10):1354-60.

800. Hirasawa A, Robinson Y, Olerud C, Wakao N, Kamiya M, Takeuchi M, et al. The prevalence of diffuse idiopathic skeletal hyperostosis among acute trauma patients in sweden. European Spine Journal. 2017;26 (2 Supplement 1):S324-S5.

801. Johansson Frigyesi E, Andersson P, Frigyesi A. Boys have better short-term and long-term survival rates after intensive care admissions than girls. Acta Paediatrica, International Journal of Paediatrics. 2017;106(12):1973-8.

802. Johnsen AS, Sollid SJM, Vigerust T, Jystad M, Rehn M. Helicopter emergency medical services in major incident management: A national Norwegian cross-sectional survey. Plos One. 2017;12(2).

803. Jonsdottir GM, Lund SH, Snorradottir B, Karason S, Olafsson IH, Reynisson K, et al. A population-based study on epidemiology of intensive care unit treated traumatic brain injury in Iceland. Acta Anaesthesiologica Scandinavica. 2017;61(4):408-17.

804. Kornhall DK, Jorgensen JJ, Brommeland T, Hyldmo PK, Asbjornsen H, Dolven T, et al. The Norwegian guidelines for the prehospital management of adult trauma patients with potential spinal injury. Scand J Trauma Resusc Emerg Med. 2017;25(1):2.

805. L Wilson M, Tenovuo O, Mattila VM, Gisler M, Celedonia KL, Impinen A, et al. Pediatric TBI in Finland: An examination of hospital discharges (1998-2012). European journal of paediatric neurology : EJPN : official journal of the European Paediatric Neurology Society. 2017;21(2):374-81.

806. Larsen TM, Bendtsen MD, Sovso M, Lindskou TA, Hansen PA, Jensen FB, et al. Treat-and-release EMS patients in the North Denmark Region: Identification and vital signs. BMJ Open. 2017;7 (Supplement 3):A13.

807. Lebmeier M, Strait LB, Hegarty LV, Fielden DJ. Budget impact analysis of low-dose methoxyflurane for the treatment of moderate-to-severe trauma pain in Sweden. Value in Health. 2017;20 (9):A548.

808. Lexell JE, Jorgensen S, Ginis KAM. Leisure time physical activity among older adults with long-term spinal cord injury. PM and R. 2017;9 (9 Supplement 1):S134-S5.

809. Lu J, Roe C, Sigurdardottir S, Andelic N. Trajectory of functional independent measurements during five years following moderate and severe traumatic brain injury. Brain Injury. 2017;31 (6-7):936.

810. Lundstrom U, Wahman K, Seiger A, Gray DB, Isaksson G, Lilja M. Participation in activities and secondary health complications among persons aging with traumatic spinal cord injury. Spinal Cord. 2017;55(4):367-72.

811. Manskow US, Friborg O, Roe C, Braine M, Damsgard E, Anke A. Patterns of change and stability in caregiver burden and life satisfaction from 1 to 2 years after severe traumatic brain injury: A Norwegian longitudinal study. NeuroRehabilitation. 2017;40(2):211-22.

812. Nellgard P, Ejnell H, Olofsson K, Hallen K, Schien M, Papatziamos G, et al. National recommendations for tracheotomy and for tracheostomy care. Acta Anaesthesiologica Scandinavica. 2017;61 (8):1034-5.

813. Nilsson P, Stigson H, Ohlin M, Strandroth J. Modelling the effect on injuries and fatalities when changing mode of transport from car to bicycle. Accident Analysis and Prevention. 2017;100:30-6.

814. Noe BB, Stapelfeldt CM, Parner ET, Mikkelsen EM. Survival after traumatic spinal cord injury in Denmark: a hospital-based study among patients injured in 1990-2012. Spinal Cord. 2017;55(4):373-7.

815. Osteras O, Heltne J-K, Vikenes B-C, Assmus J, Brattebo G. Factors influencing on-scene time in a rural Norwegian helicopter emergency medical service: a retrospective observational study. Scandinavian Journal of Trauma Resuscitation & Emergency Medicine. 2017;25.

816. Osterlund AH, Lander F, Nielsen K, Kines P, Moller J, Lauritsen J. Transient risk factors of acute occupational injuries: a case-crossover study in two Danish emergency departments. Scand J Work Environ Health. 2017;43(3):217-25.

817. Palm S, Ronnback L, Johansson B. Long-Term Mental Fatigue after Traumatic Brain Injury and Impact on Employment Status. Journal of Rehabilitation Medicine. 2017;49(3):228-33.

818. Raj R, Kaprio J, Korja M, Mikkonen ED, Jousilahti P, Siironen J. Risk of hospitalization with neurodegenerative disease after moderate-to-severe traumatic brain injury in the working- age population: A retrospective cohort study using the Finnish national health registries. Plos Medicine. 2017;14(7).

819. Rasmussen MS, Andelic N, Nordenmark TH, Arango-Lasprilla JC, Soberg HL. Family as a resource for improved patient and family functioning after traumatic brain injury: A randomized controlled trial of a family intervention-protocol and pilot study. Brain Injury. 2017;31 (6-7):849-50.

820. Rinne PP, Laitinen MK, Huttunen T, Kannus P, Mattila VM. The incidence and trauma mechanisms of acetabular fractures: A nationwide study in Finland between 1997 and 2014. Injury. 2017;48(10):2157-61.

821. Robertsen A, Forde R, Skaga NO, Helseth E. Treatment-limiting decisions in patients with severe traumatic brain injury in a Norwegian regional trauma center. Scandinavian Journal of Trauma Resuscitation & Emergency Medicine. 2017;25.

822. Rubenson Wahlin R, Nelson DW, Bellander BM, Svensson M, Thelin E. Prehospital advanced airway management in traumatic brain injury and its relation to outcome. Journal of Neurotrauma. 2017;34 (13):A124.

823. Ryosa A, Tainio J, Itala A, Gullichsen E. Clinical profile, treatment details and survival of trauma patients treated at intensive care unit in a level ii trauma center. Journal of Acute Medicine. 2017;7(1):19-23.

824. Raatiniemi L, Liisanantti J, Tommila M, Moilanen S, Ohtonen P, Martikainen M, et al. Evaluating helicopter emergency medical missions: a reliability study of the HEMS benefit and NACA scores. Acta Anaesthesiologica Scandinavica. 2017;61(5):557-65.

825. Sandstrom L, Nilsson C, Juuso P, Engstrom A. The helicopter as a caring context: Experiences of people suffering trauma. International Emergency Nursing. 2017;32:34-8.

826. Seternes A, Rekstad LC, Mo S, Klepstad P, Halvorsen DL, Dahl T, et al. Open Abdomen Treated with Negative Pressure Wound Therapy: Indications, Management and Survival. World Journal of Surgery. 2017;41(1):152-61.

827. Sigurdardottir S, Andelic N, Roe C, Schanke AK. Cognitive recovery trajectories over the first five years following moderate-to-severe traumatic brain injury. Brain Injury. 2017;31 (6-7):854-5.

828. Soberg HL, Roe C, Brunborg C, von Steinbuechel N, Andelic N. The Norwegian version of the QOLIBRI - a study of metric properties based on a 12 month follow-up of persons with traumatic brain injury. Health and Quality of Life Outcomes. 2017;15.

829. Sonne A, Wulffeld S, Steinmetz J, Rasmussen LS, Hesselfeldt R. Prehospital interventions before and after implementation of a physician-staffed helicopter. Danish Medical Journal. 2017;64(10).

830. Steinthorsdottir KJ, Svenningsen P, Fabricius R, Svendsen LB, Hillingso J, Sillesen M. Self-evaluated competence in trauma reception. Danish Medical Journal. 2017;64(11).

831. Taipale H, Koponen M, Tanskanen A, Lavikainen P, Sund R, Tiihonen J, et al. Risk of head and traumatic brain injuries associated with antidepressant use among community-dwelling persons with Alzheimer's disease: A nationwide matched cohort study. Alzheimer's Research and Therapy. 2017;9 (1) (no pagination)(59).

832. Thelin EP, Nelson DW, Bellander BM. A review of the clinical utility of serum S100B protein levels in the assessment of traumatic brain injury. Acta Neurochirurgica. 2017;159(2):209-25.

833. Thelin EP, Nelson DW, Vehvilainen J, Nystrom H, Kivisaari R, Siironen J, et al. Evaluation of novel computerized tomography scoring systems in human traumatic brain injury: An observational, multicenter study. Plos Medicine. 2017;14(8).

834. Thesleff T, Niskakangas T, Luoto T, Iverson GL, Ohman J, Ronkainen A. Preventable diagnostic errors in fatal cervical spine injuries: A nationwide register-based study from 1987 to 2010. Spine Journal. 2017.

835. Tibaek M, Forchhammer HB, Dehlendorff C, Johnsen SP, Kammersgaard LP. Incidence and mortality of acquired brain injury in young Danish adults between 1994 and 2013: a nationwide study. Brain Injury. 2017;31(11):1455-62.

835. Tolstrup MB, Watt SK, Gogenur I. Reduced Rate of Dehiscence After Implementation of a Standardized Fascial Closure Technique in Patients Undergoing Emergency Laparotomy. Annals of Surgery. 2017;265(4):821-6.

836. Weber CD, Nguyen AR, Lefering R, Hofman M, Hildebrand F, Pape HC. Blunt injuries related to equestrian sports: results from an international prospective trauma database analysis. International Orthopaedics. 2017;41(10):2105-12.

837. Wettervik TS, Lenell S, Nyholm L, Howells T, Lewen A, Enblad P. Decompressive craniectomy in traumatic brain injury: usage and clinical outcome in a single centre. Acta Neurochirurgica. 2017:1-9.

838. Wisborg T, Ellensen EN, Svege I, Dehli T. Are severely injured trauma victims in Norway offered advanced pre-hospital care? National, retrospective, observational cohort. Acta Anaesthesiologica Scandinavica. 2017;61(7):841-7.

839. Wisborg T, Manskow US, Jeppesen E. Trauma outcome research - More is needed. Acta Anaesthesiologica Scandinavica. 2017;61(4):362-4.

840. Wulffeld S, Rasmussen LS, Hojlund Bech B, Steinmetz J. The effect of CT scanners in the trauma room - an observational study. Acta Anaesthesiologica Scandinavica. 2017;61(7):832-40.

841. Young VS, Eggesbo HB, Gaarder C, Naess PA, Enden T. Radiology response in the emergency department during a mass casualty incident: a retrospective study of the two terrorist attacks on 22 July 2011 in Norway. European Radiology. 2017;27(7):2828-34.

842. Airaksinen NK, Nurmi-Luthje IS, Kataja JM, Kroger HPJ, Luthje PMJ. Cycling injuries and alcohol. Injury. 2018.

843. Dehli T, Uleberg O, Wisborg T. Trauma team activation - common rules, common gain. Acta Anaesthesiologica Scandinavica. 2018;62(2):144-6.

844. Eriksen TR, Shumba L, Ekeberg O, Bogstrand ST. The association between hospital admission and substance use among trauma patients. Journal of Substance Use. 2018;23(1):79-85.

845. Graff HJ, Christensen U, Poulsen I, Egerod I. Patient perspectives on navigating the field of traumatic brain injury rehabilitation: a qualitative thematic analysis. Disability and rehabilitation. 2018;40(8):926-34.

846. Howells T, Smielewski P, Donnelly J, Czosnyka M, Hutchinson PJA, Menon DK, et al. Optimal Cerebral Perfusion Pressure in Centers With Different Treatment Protocols. Critical Care Medicine. 2018;46(3):e235-e41.

847. Ihalainen T, Luoto TM, Rinta-Kiikka I, Ronkainen A, Korpijaakko-Huuhka AM. Traumatic cervical spinal cord injury: recovery of penetration/aspiration and functional feeding outcome. Spinal Cord. 2018:1-8.

848. Koskela A, Liisanantti JH, Koskenkari J, Ohtonen P, Mantyvaara T, Ala-Kokko T. Alcohol and other substance abuse in trauma patients admitted to ICU in Northern Finland. Journal of Substance Use. 2018;23(1):14-9.

849. Nedergaard HK, Haberlandt T, Toft P, Jensen HI. Pressure ulcers in critically ill patients - Preventable by non-sedation? A substudy of the NONSEDA-trial. Intensive & critical care nursing. 2018;44:31-5.

850. Niemi-Nikkola V, Saijets N, Ylipoussu H, Kinnunen P, Pesala J, Makela P, et al. Traumatic Spinal Injuries in Northern Finland. Spine. 2018;43(1):E45-E51.

851. Raj R, Bendel S, Reinikainen M, Hoppu S, Luoto T, Ala-Kokko T, et al. Temporal Trends in Healthcare Costs and Outcome Following ICU Admission After Traumatic Brain Injury. Critical care medicine. 2018.

852. Roislien J, Sovik S, Eken T. Seasonality in trauma admissions - Are daylight and weather variables better predictors than general cyclic effects? PLoS ONE. 2018;13 (2) (no pagination)(e0192568).

853. Skaga NO, Eken T, Sovik S. Validating performance of TRISS, TARN and NORMIT survival prediction models in a Norwegian trauma population. Acta Anaesthesiologica Scandinavica. 2018;62(2):253-66.
